# Supplementary material for: Behavioural factors associated with fear of litigation as a driver for the increased use of caesarean sections: a scoping review
Source: BMJ Open. 2023 Apr 19;13(4):e070454. doi: 10.1136/bmjopen-2022-070454 (PMC10124311; doi:10.1136/bmjopen-2022-070454)
Supplement: Supplementary data [file bmjopen-2022-070454supp001.pdf]

Supplementary file 1

Search Strategy

PubMed

("OBSTETRICS/LEGISLATION AND JURISPRUDENCE"[MESH] OR "CESAREAN SECTION"[MESH] OR "CESAREAN SECTION"[TIAB] OR "CESAREAN SECTION"[TW] OR "CESAREAN SECTIONS"[TIAB] OR "CESAREAN SECTIONS"[TW] OR "CAESAREAN"[TW] OR "DELIVERY, ABDOMINAL" [TIAB] OR "ABDOMINAL DELIVERIES" [TIAB] OR "DELIVERIES, ABDOMINAL" [TIAB] OR "CAESAREAN SECTION" [TIAB] OR "CAESAREAN SECTIONS" [TIAB] OR "ABDOMINAL DELIVERY" [TIAB] OR "C-SECTION" [TIAB] OR "C SECTION" [TIAB] OR "C-SECTIONS" [TIAB] OR "POSTCESAREAN SECTION"[TIAB])

AND

(LEGIS OR "OBSTETRICS/LEGISLATION AND JURISPRUDENCE"[MESH] OR "JURISPRUDENCE"[MESH] OR "COURT DECISION*" [TIAB] OR "LAW" [TIAB] OR "LAW"[TW] OR "LAWS"[TW] OR "LEGAL*"[TW] OR "LAWY*" [TW] OR "LITIGATION"[TW] OR "LITIGATIONS"[TW] OR "LITIGAT*"[TW] OR "LEGAL CONSEQ*"[TIAB] OR "MEDICAL JURISPRUDENCE" [TW] OR "DEFENSIVE MEDICINE"[MESH] OR "DEFENSIVE PRACTICE*"[TIAB] OR "NEGOTIATING"[MESH] OR "NEGOTIAT*" [TIAB] OR "MEDIATION" [TIAB] OR "MEDIATING" [TIAB] OR "ARBITRATING" [TIAB] OR "ARBITRATION*" [TIAB] OR "CONFLICT RESOLUTION" [TIAB] OR "CONFLICT RESOLUTIONS" [TIAB] OR "RESOLUTION, CONFLICT" [TIAB] OR "MEDICO-LEGAL"[TIAB] OR "VIOLENCE"[TIAB] OR "CASE LAW"[TIAB] OR "LEGAL CASE*"[TIAB] OR "COURT CASE"[TIAB] OR "MALPRACTICE"[MESH] OR "MALPRACTICE"[TIAB] OR "NEGLIGENCE"[TIAB] OR "NEGLIGEN*"[TIAB] OR "MALFEASANCE"[TIAB] OR "MISCONDUCT"[TW] OR "PROFESSIONAL DISCIPLINE"[TW] OR "PATIENT ADVOCACY" [TW] OR "PATIENT RIGHTS"[TW] OR "LIABILITY"[TIAB])

AND

AND PUBYEAR > 2000

Scopus

(TITLE-ABS-KEY(("Caesarean section" OR "Cesarean Section" OR "Caesarean sections" OR "Cesarean Sections" OR "C-section" OR "C-sections" OR "Abdominal delivery" OR "Abdominal deliveries"))

Elaraby S, *et al. BMJ Open* 2023; 13:e070454. doi: 10.1136/bmjopen-2022-070454

AND

TITLE-ABS-KEY(("Jurisprudence" OR "Court Decision*" OR "Law" OR "law" OR "laws" OR "legal*" OR "lawy*" OR "litigation" OR "litigations" OR "litigat*" OR "legal conseq*" OR "Medical Jurisprudence" OR "Defensive Medicine" OR "Defensive Practice*" OR "Negotiating" OR "Negotiat*" OR "Mediation" OR "Mediating" OR "Arbitrating" OR "Arbitration" OR "Arbitrations" OR "Conflict Resolution" OR "Conflict Resolutions" OR "medico-legal" OR "Violence" OR "case law" OR "legal case*" OR "court case" OR "Malpractice" OR "malpractice" OR "Negligence" OR "negligen*" OR "malfeasance" OR "misconduct" OR "professional discipline" OR "patient advocacy" OR "patient rights")))

AND PUBYEAR > 2000

WHO Global Index Medicus

(“Caesarean section” OR “Cesarean Section” OR “Caesarean sections” OR “Cesarean Sections” OR “C-section” OR “C-sections” OR “Abdominal delivery” OR “Abdominal deliveries”)

AND

(“Jurisprudence” OR “Court Decision*” OR “Law” OR “law” OR “laws” OR “legal*” OR “lawy*” OR “litigation” OR “litigations” OR “litigat*” OR “legal conseq*” OR “Medical Jurisprudence” OR “Defensive Medicine” OR “Defensive Practice*” OR “Negotiating” OR “Negotiat*” OR “Mediation” OR “Mediating” OR “Arbitrating” OR “Arbitration” OR “Arbitrations” OR “Conflict Resolution” OR “Conflict Resolutions” OR “medico-legal” OR “Violence” OR “case law” OR “legal case*” OR “court case” OR “Malpractice” OR “malpractice” OR “Negligence” OR “negligen*” OR “malfeasance” OR “misconduct” OR “professional discipline” OR “patient advocacy” OR “patient rights”)

AND

PUBYEAR > 2000

Detailed inclusion and exclusion criteria

Inclusion criteria

- Focus on caesarean section (not broadly obstetrics).
- Include fear of litigation (broadly defined) in the findings
- Include analysis of empirical data
- Include peer-reviewed articles
- Descriptive or experimental design

- Can include both quantitative designs/data:
  - *E.g.* cross sectional, cohort*,* non-randomized controlled trials; program evaluations; interrupted time-series; pre-post designs; pilot studies; etc
  - *N > 6*
- Qualitative designs/data
  - In-depth interviews; focus groups; key-informant interviews
  - *N > 1*
- And mixed methods

- Include drivers of fear of litigation (i.e. goes beyond mere mention of its existence).
  - These can include behavioural determinants or drivers.
  - Can include stakeholders or other actors influencing fear of litigation.
- Publication language: English, Spanish, Portuguese, French, or German
- Published between 2001 and 2021

Exclusion criteria

- Case-studies or case-series
- Studies that report on a study or program where main outcomes are NOT related to unnecessary caesarean sections (broadly discuss obstetric malpractice).
- Reports or articles that do not describe original research (e.g. Abstracts, communications, book chapters, news articles, etc)
- Studies with no mention of fear of litigation or its drivers (beyond its presence based on previous literature)
- Pure legal analysis that does not discuss healthcare decision-making
- Review papers with no substantial analysis of fear of litigation (but screened references for more studies)
- Publication language other than English, Spanish, Portuguese, German or French.
- Published before 2001

Variables collected (data-extraction form)

The following variables were extracted in an excel form specifically designed for this scoping review:

- Author, citation
- Year of the study
- Topic/domain
- Country or countries
- Study design
- Data type
- Sample size
- Type of participant
- Participant characteristics

Elaraby S, *et al. BMJ Open* 2023; 13:e070454. doi: 10.1136/bmjopen-2022-070454

- Behavioural drivers
- For each behavioural driver, how the behavioural drivers were assessed
- For each behavioural driver, rational or justification for effect on fear of litigation
- For each behavioural driver, other actors in relation with the behavioural driver
- Key limitations
- Key strengths
- Quotes
- Other notes

Elaraby S, *et al. BMJ Open* 2023; 13:e070454. doi: 10.1136/bmjopen-2022-070454
