# Supplementary material for: Behavioural factors associated with fear of litigation as a driver for the increased use of caesarean sections: a scoping review
Source: BMJ Open. 2023 Apr 19;13(4):e070454. doi: 10.1136/bmjopen-2022-070454 (PMC10124311; doi:10.1136/bmjopen-2022-070454)
Supplement: Supplementary data [file bmjopen-2022-070454supp002.pdf]

Supplementary file 2: Study details including behavioural drivers reported by each study.

Author, year	Country	Method/data type	Participants and sample size	Behavioural drivers for CS
Akpinar, 2021 (1)	Turkey	Survey<br>Quantitative data	404 Obstetricians and Gynaecological administrators and practitioners	Ambiguity aversion<br>Beliefs around safety<br>Legal environment<br>Limited coverage of liability insurance
Ali, 2016 (2)	Sudan	Survey<br>Quantitative data	117 OBGYN (42.7% consultants, 34.2% registrars and 23.1% specialists)	Availability bias<br>Blame Culture
Aminu et al., 2014 (3)	Bangladesh	Mixed methods (retrospective review of case notes and Key informant interviews with service providers)	18 OBGYN and case notes of 530 women who had CS in 5 Public Hospitals	Patient Pressure<br>Relative risk bias and Beliefs around safety
Asher, 2013 (4)	Israel	Survey<br>Quantitative data	117 board certified physicians and residents in OBGYN departments at tertiary hospitals	Availability bias<br>Medical and professional environment<br>Media
Bagheri, 2013 (5)	Iran	Qualitative data	18 OBGYN	Ambiguity aversion<br>Legal environment
Berger, 2012 (6)	United States	Survey<br>Quantitative data	Nurses at 243 civilian California birth hospitals	Availability bias<br>Medical and professional environment<br>Malpractice insurance premiums
Bastos-Dias, 2004 (7)	Brazil	Qualitative data	OBGYNs practicing in one public maternity in Rio de Janeiro, caring for women with low education	Beliefs around safety<br>Ambiguity aversion<br>Social norms
Behague, 2002 (8)	Brazil	Mixed methods	80 mothers from a birth cohort of 5304 women who gave birth in 1993 in any of the hospitals in Pelotas, Brazil. As well as 19 clinicians (six	Patient Pressure<br>Media

Elaraby S, et al. BMJ Open 2023; 13:e070454. doi: 10.1136/bmjopen-2022-070454

			OBGYN, six paediatricians, four general practitioners, and three nurses)	
Bermúdez-Tamayo, 2017 (9)	Spain	Qualitative data	14 OBGYN and 14 nurse-midwives (NMW) who work for Spain's National Healthcare System	Legal environment<br>Commission bias<br>Loss/gain framing or loss aversion bias<br>Social norms<br>Beliefs around safety
Bettes, 2007 (10)	United States	Survey<br>Quantitative data	699 OBGYN	Patient pressure
Brown, 2007 (11)	United States	Secondary analysis<br>Quantitative data	Hospital discharges data and Insurance Closed Claim File data. Analysis was conducted using a random sample, with replacement of 20,000 births out of the total birth population of 260,292	Availability bias
Carrera, 2017 (12)	United States (Puerto Rico)	Survey<br>Quantitative data	62 OBGYN	Patient pressure<br>Legal System<br>Availability bias<br>Malpractice insurance/premiums
Chaillet, Dube (13)	Canada	Qualitative data	10 Focus group discussions (FGDs) and 6 in-depth interviews (IDIs)	Patient pressure<br>Ambiguity aversion<br>Medical and professional environment
Chen and Yang (14)	Taiwan	Panel analysis<br>Quantitative data	Random subsamples of population of outpatient and inpatient claim in Taiwan (1997-2005) out of 1 million random individual medical claim histories	Legal environment
Cheng, 2014 (15)	United States	Survey<br>Quantitative data	1486 clinicians (general OBGYN, Maternal-Foetal Medicine specialists, family medicine physicians and clinical nurse midwives), who practice obstetrics in the US and who were registered as active members of the American Medical Association (AMA).	Availability bias<br>Malpractice insurance/premiums<br>Legal environment

Colomar, 2014 (16)	Nicaragua	Qualitative data	4 FGDs, and 17 IDIs with OBGYN and medical decision makers	Patient Pressure<br>Ambiguity aversion<br>Loss/gain framing or loss aversion bias<br>Medical and professional environment
Cox, 2011 (17)	United States	Semi-structured interviews<br><br>Qualitative data	11 obstetricians, 12 midwives, and a hospital administrator (n = 24)	Legal environment<br>Ambiguity aversion<br>Blame Culture
Deutch, 2011 (18)	United States	Survey<br><br>Quantitative data	510 OBGYN (Society for Maternal–Foetal Medicine (SMFM) members)	Legal environment
Doraiswany, 2021 (19)	Bangladesh	IDIs and Labour observations<br><br>Qualitative data	16 physicians and 32 women who delivered by CS	Patient pressure<br>Risk aversion bias (loss gain framing)
Dranove, 2010 (20)	United States	Quantitative record analysis	Using patient-level hospital discharge data and matching physician license of presiding OBGYN to data of closed medical malpractice claims. Examined data of 1539 unique physicians who carried out 98% of the included birth records.	Availability bias<br>Medical and professional environment
Durrance, 2018(21)	United States	Quantitative data	Around 2 million hospital deliveries linked to physician malpractice claim histories (around 2300 doctors) between 1994 and 2010	Availability bias
Esposto, 2012 (22)	United States	Quantitative data	Panel data of CS rates in three years (1987, 1990 and 1993) in 50 states	Legal System (tort reform)
Fineschi, 2021 (23)	Italy	Quantitative data	168 OBGYN	Legal system<br>Patient pressure<br>Relative risk bias and beliefs around safety
Firoozi et al, 2020 (24)	Iran	IDIs and an FGD<br><br>Qualitative data	26 IDIs with maternity care providers (OBGYN and Midwives) and mothers with prior CS	Legal environment<br>Ambiguity aversion<br>Blame culture

Frakes, 2012 (25)	United States	Secondary quantitative data	Using data on physician behaviour from the 1979–2005 National Hospital Discharge Surveys (NHDS),	Availability bias (Experience with litigation)<br>Legal environment (tort reform)
Fuglenes, 2009 (26)	Norway	Survey<br><br>Quantitative data	716 OBGYN	Perceived risk of litigation (Fear Index)
Gimm, 2010 (27)	United States	Secondary data<br><br>Panel Analysis	Secondary data from the 1992–2000 Florida Hospital Inpatient Discharge File, the Florida Medical Professional Liability Insurance Claims File, and the American Medical Association's Master File on physician characteristics.	Availability bias (Experience with litigation)
Goldenberg, 2016 (28)	Paraguay	IDIs<br><br>Qualitative data	10 OBGYN	Commission bias<br>Loss/gain framing or loss aversion bias<br>Ambiguity aversion<br>Patient pressure
Grant, 2004 (29)	United States	Panel Analysis<br><br>Quantitative data	835 obstetricians who performed at least 10 births in each year and had all the information on physician characteristics	Availability bias
Habiba, 2006 (30)	Luxembourg, Netherlands and Sweden, France, Germany, Italy, Spain and UK	self-administered questionnaire<br><br>Quantitative data	One hundred and five units Neonatal Intensive Care Unit (NICU) associated maternity units, and 1530 obstetricians participated in the study in eight European countries.	Patient pressure
Hellerstein, 2016 (31)	China	Survey and qualitative observations<br><br>Mixed methods data	Descriptive structured survey of obstetric services with standardized interview with obstetricians and qualitative observation of obstetric services in six different Shanghai obstetric facilities	Patient (and family) pressures<br>Ambiguity aversion<br>Social norms regarding birth

.				
Indraccolo, 2015 (32)	Italy	Survey<br>Quantitative data	36 OBGYNs, 42 midwives, 22 lawyers and 25 patients	Patient pressure
Ionsecu, 2018 (33)	Romania	Survey<br>Quantitative data	73 OBGYNs	Patient pressure<br>Relative risk bias and beliefs around safety<br>Legal environment
Kamal, 2005 (34)	United Kingdom	IDIs<br>Qualitative data	Trainee and consultant obstetricians, and community and hospital-based midwives involved in the care of women in pregnancy and labour in Leicestershire,	Ambiguity aversion<br>Blame Culture
Karlstro¨m, 2009 (35)	Sweden	FGDs<br>Qualitative data	5 FGDs where 16 midwives and 9 obstetricians participated.	Ambiguity aversion<br>Patient pressure
Koigi-Kamau, 2005 (36)	Kenya	Survey<br>Quantitative data	64 obstetricians with individual private practices (i.e., independent of institutional policies) from the first major towns in Kenya – namely Nairobi, Mombasa, Kisumu, Nakuru and Eldoret.	Ambiguity aversion
Kuan (37)	Taiwan	Multiple Qualitative methods	Interviews with eight obstetricians, two nurses, a doula, a midwife, and 34 pregnant women along with document review, and ethnographic observations	Patient pressure<br>Ambiguity aversion
Kucuk (38)	Turkey	Survey<br>Quantitative data	100 obstetricians who responded to the survey.	Legal environment<br>Ambiguity aversion
Kucuk (39)	Turkey	Survey<br>Quantitative data	108 practicing OBGYNs	Availability bias<br>Malpractice insurance/premiums<br>Legal environment

Kwee, Cohlen (40)	Netherlands	Survey<br>Quantitative data	583 OBGYN (80% consultants and 20% were senior registrar	Patient pressure
Lundgren, 2016 (41)	Ireland, Italy, and Germany	FGDs<br>Qualitative methods	FGDs were held in Ireland, Italy and Germany. In total 71 clinicians participated in nine FGDs.	Social Norms regarding birth<br>Blame culture<br>Patient pressure
Lundgren, 2015 (42)	Finland, Sweden, and the Netherlands	IDIs (face to face and over the telephone)<br>FGFs<br>Qualitative methods	26 midwives and 18 obstetricians.	Legal System<br>Blame culture (absence of)<br>Media
Makhija et al., 2019 (43)	India	Mixed methods (birth data and IDIs)	20 consultant OBGYNs	Patient pressure
Munro, 2017 (44)	Canada	Qualitative methods	35 Family physicians, midwives, obstetricians, nurses, anaesthetists, and health service decision makers recruited from 3 rural and 2 urban Canadian communities	Availability bias<br>Ambiguity aversion
Mushiniski, 2021 (45)	United States	Quantitative data		Legal environment
Panda, Daly (46)	Sweden	FGDs<br>Qualitative methods	4 FGDs with OBGYNs and Midwives involved in birth decision making	Legal environment
Peel, Bhartia (47)	India	IDIs<br>Qualitative methods	14 OBGYN and providers involved in maternity care	Relative Risk bias and beliefs around safety<br>Commission bias
Perrotta, 2021 (48)	Argentina	Key informant interviews (KII) and Surveys<br>Mixed methods	680 surveys and 26 KIIs of heads of hospital departments, OBGYN, midwives and residents	Legal system<br>Relative Risk bias and beliefs around safety<br>Malpractice insurance/premiums and coverage

Rudey, 2021 (49)	Brazil	Survey<br>Quantitative data	403 General obstetricians who are members of premier Brazilian professional associations of gynaecology and obstetrics	Commission bias<br>Loss/gain framing or loss aversion bias<br>Ambiguity aversion<br>Availability bias<br>Legal environment<br>Media
Samadi, 2013 (50)	Iran	Survey<br>Quantitative data	75 OBGYN	Availability bias (Experience with litigation)<br>Legal environment
Savage, 2007 (51)	United Kingdom	Mixed methods	151 OBGYN and clinical directors in OBGYN listed by the royal college of OBGTN in British Isles	Experience with litigation (financial and time drain)<br>Legal environnement<br>Patient pressure<br>Media
Wells, 2010 (52)	United States	Survey<br>Quantitative data	458 practicing obstetricians	Availability bias (Experience with litigation)
Yang, 2009 (53)	United States	Quantitative data	52 million recorded births state-level longitudinal mixed-effects regression models to examine data from the Natality Detail File on births in the United States (1991–2003)	Malpractice insurance/premiums<br>Legal environment
Yazdizadeh, 2011 (54)	Iran	IDIs and document analysis<br>Qualitative data	26 providers from various public and private hospitals responding to the ministry's C-section reduction interventions. Participants included hospital director, obstetricians and midwives.	Availability bias (Experience with litigation)<br>Legal environment (judges' capability, unrealistic expectation from medical professionals, and large settlements)<br>Patient pressure
Zhao, 2021(55)	China	Survey<br>Quantitative data	606/1412 anesthesiologists , 495 obstetricians, 219 midwives and labor & delivery nurses, 1412 hospital administrators and 59 specialties unspecified.	Legal environment<br>Patient pressure<br>Relative risk bias and beliefs around safety

Zwecker, 2011 (56)	United States	Quantitative record data	890,266 records of women who delivered and malpractice premium data in 37 states in 2006	Malpractice insurance/premiums

References

1. Akpinar F, Kilic F, Ozturk N, Coskun B, Aksar M, Erkaya S. The effect of medical and social conditions on the mode of delivery: a prospective questionnaire-based study applied to 404 Turkish obstetricians. Archives of gynecology and obstetrics. 2021;303(5):1167-74.
2. Ali AA, Hummeida ME, Elhassan YA, Nabag WO, Ahmed MA, Adam GK. Concept of defensive medicine and litigation among Sudanese doctors working in obstetrics and gynecology. BMC Med Ethics. 2016;17:12.
3. Aminu M, Utz B, Halim A, van den Broek N. Reasons for performing a caesarean section in public hospitals in rural Bangladesh. BMC Pregnancy Childbirth. 2014;14(1):130.
4. Asher E, Dvir S, Seidman DS, Greenberg-Dotan S, Kedem A, Sheizaf B, et al. Defensive medicine among obstetricians and gynecologists in tertiary hospitals. PloS one. 2013;8(3):e57108.
5. Bagheri A, Masoudi Alavi N, Abbaszadeh F. Iranian obstetricians' views about the factors that influence pregnant women's choice of delivery method: a qualitative study. Women Birth. 2013;26(1):e45-9.
6. Barger MK, Dunn JT, Bearman S, DeLain M, Gates E. A survey of access to trial of labor in California hospitals in 2012. BMC Pregnancy Childbirth. 2013;13:83.
7. Dias MA, Deslandes SF. [Cesarean sections: risk perception and indication by attending obstetricians in a public maternity hospital in Rio de Janeiro]. Cadernos de saude publica. 2004;20(1):109-16.
8. Behague DP, Victora CG, Barros FC. Consumer demand for caesarean sections in Brazil: informed decision making, patient choice, or social inequality? A population based birth cohort study linking ethnographic and epidemiological methods. BMJ (Clinical research ed). 2002;324(7343):942-5.
9. Bermudez-Tamayo C, Fernandez Ruiz E, Pastor Moreno G, Maroto-Navarro G, Garcia-Mochon L, Perez-Ramos FJ, et al. Barriers and enablers in the implementation of a program to reduce cesarean deliveries. Reprod Health. 2017;14(1):106.
10. Bettes BA, Coleman VH, Zinberg S, Spong CY, Portnoy B, DeVoto E, et al. Cesarean delivery on maternal request: obstetrician-gynecologists' knowledge, perception, and practice patterns. Obstet Gynecol. 2007;109(1):57-66.
11. Brown HS, 3rd. Lawsuit activity, defensive medicine, and small area variation: the case of Cesarean sections revisited. Health economics, policy, and law. 2007;2(Pt 3):285-96.
12. Carrera AM, Sternke EA, Rivera-Viñas JI. A pilot study of the perceptions of actively practicing obstetricians in Puerto Rico: factors that influence decision making in cesarean delivery. Puerto Rico Health Sciences Journal. 2017;36(1).
13. Chaillet N, Dube E, Dugas M, Francoeur D, Dube J, Gagnon S, et al. Identifying barriers and facilitators towards implementing guidelines to reduce caesarean section rates in Quebec. Bulletin of the World Health Organization. 2007;85(10):791-7.
14. Chen BK, Yang C-Y. Increased Perception of Malpractice Liability and the Practice of Defensive Medicine. Journal of Empirical Legal Studies. 2014;11(3):446-76.
15. Cheng YW, Snowden JM, Handler SJ, Tager IB, Hubbard AE, Caughey AB. Litigation in obstetrics: does defensive medicine contribute to increases in cesarean delivery? The journal of maternal-fetal & neonatal medicine : the official journal of the European Association of Perinatal Medicine, the Federation of Asia and Oceania Perinatal Societies, the International Society of Perinatal Obstet. 2014;27(16):1668-75.
16. Colomar M, Cafferata ML, Aleman A, Castellano G, Elorrio EG, Althabe F, et al. Mode of childbirth in low-risk pregnancies: Nicaraguan physicians' viewpoints. Maternal and child health journal. 2014;18(10):2382-92.
17. Cox KJ. Providers' perspectives on the vaginal birth after cesarean guidelines in Florida, United States: a qualitative study. BMC Pregnancy Childbirth. 2011;11:72.

18. Deutsch AB, Duncan K, Rajaram L, Salihu HM, Spellacy WN, Belogolovkin V. Cesarean or vaginal delivery for the breech fetus at the threshold of viability: results from a maternal-fetal medicine specialists survey. The journal of maternal-fetal & neonatal medicine : the official journal of the European Association of Perinatal Medicine, the Federation of Asia and Oceania Perinatal Societies, the International Society of Perinatal Obstet. 2011;24(3):475-9.
19. Doraiswamy S, Billah SM, Karim F, Siraj MS, Buckingham A, Kingdon C. Physician–patient communication in decision-making about Caesarean sections in eight district hospitals in Bangladesh: a mixed-method study. Reproductive health. 2021;18(1):1-14.
20. Dranove D, Watanabe Y. Influence and Deterrence: How Obstetricians Respond to Litigation against Themselves and Their Colleagues. American Law and Economics Review. 2010;12(1):69-94.
21. Durrance CP, Hankins S. Medical Malpractice Liability Exposure and OB/GYN Physician Delivery Decisions. Health services research. 2018;53(4):2633-50.
22. Esposto AG. Tort reform and caesarean deliveries. Applied Economics Letters. 2012;19(12):1171-4.
23. Fineschi V, Arcangeli M, Di Fazio N, Del Fante Z, Fineschi B, Santoro P, et al. Defensive Medicine in the Management of Cesarean Delivery: A Survey among Italian Physicians. Healthcare (Basel, Switzerland). 2021;9(9).
24. Firoozi M, Tara F, Ahanchian MR, Latifnejad Roudsari R. Health Care System Barriers to Vaginal Birth after Cesarean Section: A Qualitative Study. Iranian journal of nursing and midwifery research. 2020;25(3):202-11.
25. Frakes M. Defensive medicine and obstetric practices. Journal of Empirical Legal Studies. 2012;9(3):457-81.
26. Fuglenes D, Oian P, Kristiansen IS. Obstetricians' choice of cesarean delivery in ambiguous cases: is it influenced by risk attitude or fear of complaints and litigation? Am J Obstet Gynecol. 2009;200(1):48 e1-8.
27. Gimm GW. The impact of malpractice liability claims on obstetrical practice patterns. Health services research. 2010;45(1):195-211.
28. Goldenberg T, Foster J, Andes KL. Driving birth: Cesarean sections and the medicalization of birth in Gran Asunción, Paraguay. Revista Salud Pública del Paraguay. 2016;6(1):8-15.
29. Grant D, McInnes MM. Malpractice experience and the incidence of cesarean delivery: a physician-level longitudinal analysis. Inquiry : a journal of medical care organization, provision and financing. 2004;41(2):170-88.
30. Habiba M, Kaminski M, Da Fre M, Marsal K, Bleker O, Librero J, et al. Caesarean section on request: a comparison of obstetricians' attitudes in eight European countries. BJOG : an international journal of obstetrics and gynaecology. 2006;113(6):647-56.
31. Hellerstein S, Feldman S, Duan T. Survey of Obstetric Care and Cesarean Delivery Rates in Shanghai, China. Birth (Berkeley, Calif). 2016;43(3):193-9.
32. Indraccolo U, Scutiero G, Matteo M, Indraccolo SR, Greco P. Cesarean section on maternal request: should it be formally prohibited in Italy? Annali dell'Istituto superiore di sanita. 2015;51(2):162-6.
33. Ionescu CA, Dimitriu M, Poenaru E, Banacu M, Furau GO, Navolan D, et al. Defensive caesarean section: A reality and a recommended health care improvement for Romanian obstetrics. Journal of evaluation in clinical practice. 2019;25(1):111-6.
34. Kamal P, Dixon-Woods M, Kurinczuk JJ, Oppenheimer C, Squire P, Waugh J. Factors influencing repeat caesarean section: qualitative exploratory study of obstetricians' and midwives' accounts. BJOG : an international journal of obstetrics and gynaecology. 2005;112(8):1054-60.
35. Karlstrom A, Engstrom-Olofsson R, Nystedt A, Thomas J, Hildingsson I. Swedish caregivers' attitudes towards caesarean section on maternal request. Women Birth. 2009;22(2):57-63.
36. Koigi-Kamau R, Leting PK, Kiarie JN. Perceptions and practices of vaginal birth after Caesarean section among privately practicing obstetricians in Kenya. East African medical journal. 2005;82(12):631-6.

37. Kuan CI. "Suffering twice": the gender politics of cesarean sections in Taiwan. Med Anthropol Q. 2014;28(3):399-418.
38. Kucuk M. Obstetrician perceptions of the causes of high cesarean delivery rates in Turkey. International journal of gynaecology and obstetrics: the official organ of the International Federation of Gynaecology and Obstetrics. 2017;138(1):100-6.
39. Kucuk M. Defensive medicine among obstetricians and gynaecologists in Turkey. Journal of obstetrics and gynaecology : the journal of the Institute of Obstetrics and Gynaecology. 2018;38(2):200-5.
40. Kwee A, Cohlen BJ, Kanhai HH, Bruinse HW, Visser GH. Caesarean section on request: a survey in The Netherlands. European journal of obstetrics, gynecology, and reproductive biology. 2004;113(2):186-90.
41. Lundgren I, Healy P, Carroll M, Begley C, Matterne A, Gross MM, et al. Clinicians' views of factors of importance for improving the rate of VBAC (vaginal birth after caesarean section): a study from countries with low VBAC rates. BMC Pregnancy Childbirth. 2016;16(1):350.
42. Lundgren I, van Limbeek E, Vehvilainen-Julkunen K, Nilsson C. Clinicians' views of factors of importance for improving the rate of VBAC (vaginal birth after caesarean section): a qualitative study from countries with high VBAC rates. BMC Pregnancy Childbirth. 2015;15(1):196.
43. Makhija B, Verma D, Mustafa A. A root cause analysis of increasing caesarean section rates in a tertiary care private hospital in North India. International Journal of Reproduction, Contraception, Obstetrics and Gynecology. 2019;8(11):4531.
44. Munro S, Kornelsen J, Corbett K, Wilcox E, Bansback N, Janssen P. Do Women Have a Choice? Care Providers' and Decision Makers' Perspectives on Barriers to Access of Health Services for Birth after a Previous Cesarean. Birth (Berkeley, Calif). 2017;44(2):153-60.
45. Mushinski D, Zahran S, Frazier A. Physician behaviour, malpractice risk and defensive medicine: an investigation of cesarean deliveries. Health economics, policy, and law. 2021:1-19.
46. Panda S, Daly D, Begley C, Karlström A, Larsson B, Bäck L, et al. Factors influencing decision-making for caesarean section in Sweden – a qualitative study. BMC Pregnancy and Childbirth. 2018;18(1):377.
47. Peel A, Bhartia A, Spicer N, Gautham M. 'If I do 10–15 normal deliveries in a month I hardly ever sleep at home.' A qualitative study of health providers' reasons for high rates of caesarean deliveries in private sector maternity care in Delhi, India. BMC Pregnancy and Childbirth. 2018;18(1):470.
48. Perrotta C, Romero M, Sguassero Y, Straw C, Gialdini C, Righetti N, et al. Caesarean birth in public maternities in Argentina: a formative research study on the views of obstetricians, midwives and trainees. BMJ open. 2022;12(1):e053419.
49. Rudey EL, Leal MDC, Rego G. Defensive medicine and cesarean sections in Brazil. Medicine. 2021;100(1):e24176.
50. Samadi S, Gholizadeh N, Shoar N, Shoar S. Attitudes of Obstetricians toward Cesarean Delivery in Challenging Cases. J Obstet Gynaecol India. 2013;63(5):301-5.
51. Savage W, Francome C. British consultants' attitudes to caesareans. Journal of obstetrics and gynaecology : the journal of the Institute of Obstetrics and Gynaecology. 2007;27(4):354-9.
52. Wells CE. Vaginal birth after cesarean delivery: views from the private practitioner. Semin Perinatol. 2010;34(5):345-50.
53. Yang YT, Mello MM, Subramanian SV, Studdert DM. Relationship between malpractice litigation pressure and rates of cesarean section and vaginal birth after cesarean section. Med Care. 2009;47(2):234-42.
54. Yazdizadeh B, Nedjat S, Mohammad K, Rashidian A, Changizi N, Majdzadeh R. Cesarean section rate in Iran, multidimensional approaches for behavioral change of providers: a qualitative study. BMC Health Serv Res. 2011;11(1):159.

Elaraby S, *et al. BMJ Open* 2023; 13:e070454. doi: 10.1136/bmjopen-2022-070454

55. Zhao P, Cai Z, Huang A, Liu C, Li H, Yang S, et al. Why is the labor epidural rate low and cesarean delivery rate high? A survey of Chinese perinatal care providers. PloS one. 2021;16(5):e0251345.
56. Zwecker P, Azoulay L, Abenhaim HA. Effect of fear of litigation on obstetric care: a nationwide analysis on obstetric practice. American journal of perinatology. 2011;28(4):277-84.
