# Supplementary material for: Behavioural factors associated with fear of litigation as a driver for the increased use of caesarean sections: a scoping review
Source: BMJ Open. 2023 Apr 19;13(4):e070454. doi: 10.1136/bmjopen-2022-070454 (PMC10124311; doi:10.1136/bmjopen-2022-070454)
Supplement: Author's manuscript [file bmjopen-2022-070454.draft_revisions.pdf]

BMJ Open is committed to open peer review. As part of this commitment we make the peer review history of every article we publish publicly available.

When an article is published we post the peer reviewers' comments and the authors' responses online. We also post the versions of the paper that were used during peer review. These are the versions that the peer review comments apply to.

The versions of the paper that follow are the versions that were submitted during the peer review process. They are not the versions of record or the final published versions. They should not be cited or distributed as the published version of this manuscript.

BMJ Open is an open access journal and the full, final, typeset and author-corrected version of record of the manuscript is available on our site with no access controls, subscription charges or pay-per-view fees (http://bmjopen.bmj.com).

If you have any questions on BMJ Open's open peer review process please email info.bmjopen@bmj.com

BMJ Open

Behavioural factors associated with fear of litigation as a driver for the increased use of caesarean sections: A scoping review

Journal:	*BMJ Open*
Manuscript ID	bmjopen-2022-070454
Article Type:	Original research
Date Submitted by the Author:	25-Nov-2022

Complete List of Authors:	Elaraby, Sarah; Alexandria University Faculty of Medicine, Community Medicine Department; World Health Organization, Behavioural Insights Unit<br>Altieri, Elena; World Health Organization, Behavioural Insights Unit<br>Downe, Soo; University of Central Lancashire<br>Erdman, Joanna; Dalhousie University<br>Mannava, Sunny; University of Hyderabad<br>Moncrieff, Gill; University of Central Lancashire<br>BR, Shamanna; University of Hyderabad, School of Medical Sciences<br>Torloni, Maria Regina; Sao Paulo Federal University, Department of Medicine<br>Betran, Ana Pilar; World Health Organization

Keywords:	Maternal medicine < OBSTETRICS, PUBLIC HEALTH, QUALITATIVE RESEARCH

SCHOLARONE™
Manuscripts

I, the Submitting Author has the right to grant and does grant on behalf of all authors of the Work (as defined in the below author licence), an exclusive licence and/or a non-exclusive licence for contributions from authors who are: i) UK Crown employees; ii) where BMJ has agreed a CC-BY licence shall apply, and/or iii) in accordance with the terms applicable for US Federal Government officers or employees acting as part of their official duties; on a worldwide, perpetual, irrevocable, royalty-free basis to BMJ Publishing Group Ltd ("BMJ") its licensees and where the relevant Journal is co-owned by BMJ to the co-owners of the Journal, to publish the Work in this journal and any other BMJ products and to exploit all rights, as set out in our licence.

The Submitting Author accepts and understands that any supply made under these terms is made by BMJ to the Submitting Author unless you are acting as an employee on behalf of your employer or a postgraduate student of an affiliated institution which is paying any applicable article publishing charge ("APC") for Open Access articles. Where the Submitting Author wishes to make the Work available on an Open Access basis (and intends to pay the relevant APC), the terms of reuse of such Open Access shall be governed by a Creative Commons licence – details of these licences and which Creative Commons licence will apply to this Work are set out in our licence referred to above.

Other than as permitted in any relevant BMJ Author's Self Archiving Policies, I confirm this Work has not been accepted for publication elsewhere, is not being considered for publication elsewhere and does not duplicate material already published. I confirm all authors consent to publication of this Work and authorise the granting of this licence.

For peer review only

Behavioural factors associated with fear of litigation as a driver for the increased use of caesarean sections: A scoping review

Authors

Sarah Elaraby[1, 2]

Elena Altieri[2]

Soo Downe[3]

Joanna Erdman[4]

Sunny Mannava[5]

Gill Moncrieff [3]

BR Shamanna[5]

Maria Regina Torloni[6]

Ana Pilar Betrán[7*]

* Corresponding author: Ana Pilar Betrán; betrana@who.int

Afiliations

1. Community Medicine Department, Alexandria Faculty of Medicine, Egypt
2. Behavioural Insights Unit, World Health Organization, Switzerland
3. University of Central Lancashire, United Kingdom
4. Dalhousie University, Canada
5. University of Hyderabad, India
6. Evidence Based Healthcare Post-Graduate Program, São Paulo Federal University Brazil
7. UNDP/UNFPA/UNICEF/World Bank Special Program of Research, Development and Research Training in Human Reproduction (HRP), Department of Sexual and Reproductive Health and Research, World Health Organization, Switzerland

For peer review only - http://bmjopen.bmj.com/site/about/guidelines.xhtml

Abstract

Objective: To explore the behavioural drivers of fear of litigation (FoL) among healthcare providers influencing CS rates.

Design: Scoping review.

Data sources: We searched MEDLINE, Scopus, and WHO Global Index (January 1, 2001-March 9th, 2022).

Data extraction and synthesis: Data were extracted using a custom form and we conducted content analysis using textual coding for relevant themes. We used the WHO principles for the adoption of a behavioural science perspective in public health developed by the WHO Technical Advisory Group for Behavioural Sciences and Insights to organize and analyse the findings. We used a narrative approach to summarize the findings.

Results: We screened 2968 citations, and 56 were included. Reviewed articles did not use a standard measure of influence of FoL on provider's behaviour. None of the studies used a clear theoretical framework to discuss the behavioural drivers of FoL. We identified 12 drivers under the three domains of the WHO principles: 1) Cognitive drivers: availability bias, ambiguity aversion, relative risk bias, commission bias, and loss aversion bias; 2) social and cultural drivers: patient pressure, social norms, and blame culture; and 3) environmental drivers: legal, insurance, medical and professional, and media. Cognitive biases were the most discussed drivers of FoL, followed by legal environment, and patient pressure.

Conclusions: Despite lack of consensus on a definition or measurement, we found that FoL as a driver for rising CS rates results from a complex interaction between cognitive, social, and environmental drivers. FoL may be generated less by the actual risk of litigation and more by how the providers perceive this risk and its potential consequences of the process itself independently of the actual legal outcome. Many of our findings were transferable

For peer review only - http://bmjopen.bmj.com/site/about/guidelines.xhtml

across geographical and practice settings. Behavioural interventions that consider these drivers are crucial to address FoL as part of strategies to reduce CS.

Keywords: Caesarean sections, litigation, behavioural drivers, scoping review

Summary: Strengths and limitations

- Fear of litigation (FoL) is a well-documented cause for increased rate of caesarean sections (CS) globally. To our knowledge, this is the first review to systematically explore global accounts for FoL and its behavioural drivers.
- This scoping review employs a behavioural approach and the WHO behavioural principles to identify and classify the key cognitive, social and environmental drivers of FoL among health care providers offering clear pathways for the design of interventions and health policies to address these fears and decrease rates of unnecessary CS.
- We may have overlooked evidence from grey-literature and other informal publications.
- While our interest was the understanding the drivers of medically unnecessary CS, the reviewed literature often did not differentiate between necessary and unnecessary CS. Hence, we have approached our study of drivers of FoL in relation to “increasing use of CS” rather than “increasing rates of unnecessary CS”.

For peer review only

For peer review only - http://bmjopen.bmj.com/site/about/guidelines.xhtml

Introduction

Over the past decades, rates of caesarean section (CS) have been rising steadily worldwide, in many cases without medical indication (1). The medically unnecessary use of CS impacts the quality of care and is a burden on often stretched healthcare systems (2), in addition to exposing women and offspring to the short- and long-term risks associated with this surgery (3, 4).

Medical litigation can be defined as a legal dispute that involved carrying out a lawsuit or civil action against healthcare providers or a medical entity (5). We use litigation here as an umbrella term to cover a variety of lawsuits including medical malpractice, negligence, and the associated legal liability, risk and settlements.

Fear of litigation (FoL) has been commonly recognized as an influential factor in medical decision making (6). Obstetrics is one of the leading medical specialities in terms of litigation (7). FoL and defensive medicine practices are commonly cited reasons for performing CS (8-10), sometimes without medical indication (8, 11, 12). Beyond mode of birth (MoB) decision-making, FoL has profound impact maternity care, with studies across the world documenting high rates of defensive medicine (13-18), a decline in the desire to practice obstetrics or early retirement of practicing obstetricians due to litigation concerns (11, 19).

Despite the frequent emphasis on FoL as a driver of increased CS, there is no agreed definition or a standardized tool to measure it, which reduces the ability to assess its role as a driver of CS rates. For the purpose of this review, we rely on explicit mentions of FoL and its consequences in relation to MoB decision-making.

Behavioural drivers of fear of litigation

Evidence from behavioural science indicates that our decisions and behaviours are often not deliberate and rational, but rather automatic and profoundly influenced by our environment

For peer review only

For peer review only - http://bmjopen.bmj.com/site/about/guidelines.xhtml

(8, 20). Understanding human behaviour is essential to improve the design and implementation of interventions aiming to influence decision making of providers. To improve understanding of FoL in relation to MoB, and map existing evidence that could inform interventions to optimize the use of CS, we conducted a scoping review asking the following questions: a) how is FoL defined by providers in relation to MoB? b) What are the various behavioural drivers influencing FoL and affecting decision making for MoB?

We used the principles for the adoption of a behavioural science perspective in public health developed by WHO as a guideline to organize and analyse the findings of the review (21). Based on these behavioural principles, we mapped the different types of influences on behaviour, namely cognitive and psychological factors, social and cultural factors, and environment factors driving FoL among providers. The findings of this review further informed this initial mapping.

Methods

The scoping review is reported in adherence to Preferred Reporting Items for Systematic Reviews and Meta-Analyses for Scoping reviews (PRISMA-ScR) (22) (23).

Data sources and searches

We searched three electronic databases (MEDLINE via PubMed, Scopus, and WHO Global Index) for studies published from January 1, 2001, to March 9th, 2022. The search strategy used two main concepts (and their synonyms): "caesarean section" and "litigation" (detailed search strategy in the Supplementary file 1). We also screened the reference lists of reviews and included articles for potentially relevant studies not captured by the electronic search.

Selection criteria

For peer review only

For peer review only - http://bmjopen.bmj.com/site/about/guidelines.xhtml

The review included primary research assessing drivers of FoL experienced by providers in relation to MoB or decision-making for CS. We included studies published after 2000 with qualitative, quantitative, or mixed-method designs. Studies from any country or region were eligible for inclusion. Studies were included if they were published in English, Spanish, Portuguese or French. We excluded case reports or case-series (N<6), reports not describing original research (e.g. commentaries, communications, news articles, reviews, etc.), or if they only covered pure legal analysis (i.e. not reporting original data regarding decision-making). Reviews were excluded but their reference list screened for eligible studies. We included articles covering maternity care by providers in any cadre and involved in any health setting or sector.

Study identification and data extraction

Citations retrieved from electronic databases were uploaded into Covidence (Veritas Health Innovation, Melbourne, Australia) and duplicates were excluded. Titles and abstracts were screened in duplicate, and full texts of potentially relevant studies were obtained and assessed by two reviewers independently (SE and APB). Discrepancies were discussed until consensus was reached.

A data-extraction form was specifically designed for this review to capture key aspects of the studies (setting, design, participants, behavioural drivers, and factors affecting FoL; detailed variables extracted in the Supplementary file). We used content analysis to identify emerging themes in the articles, assisted by Atlas.ti 9®, for further analysis of FoL, its drivers and how they influence decision making regarding MoB (24, 25). We developed a qualitative codebook informed by previous relevant reviews (8, 20), and the behavioural principles described before (26, 27). The codebook was iteratively modified as themes emerged inductively during the process of extraction of the included articles in this scoping review.

For peer review only - http://bmjopen.bmj.com/site/about/guidelines.xhtml

Data synthesis

Using the qualitative coding process, along with the extraction sheet, it was possible to identify and compare different behavioural drivers of FoL within and across studies (24, 25). This started with applying the codebook and extraction sheet to a few studies, and then going through an iterative process, with continuous revision of codes and overarching themes as more studies were analysed. Discussions among co-authors informed the classification of drivers according to the WHO principles and the definition of how they interact.

In this manuscript, "litigation" and associated terms are used in a non-specific or technical way reflecting their use in the studies reviewed which is often from the perspective of non-legally trained person, namely providers. It also serves as a construct that covers the risk of legal process, and the experience of litigation itself, rather than specific litigation outcomes.

Patient and public involvement

Patients and/or the public were not involved in the design, or conduct, or reporting, or dissemination plans of this scoping review.

Results

A total of 2968 citation were identified (2944 from electronic databases and 24 from other sources). We selected 440 studies for full-text evaluation and included 56 in this review (Fig 1).

Main characteristics of included studies

Table 1 shows the main characteristics of the 56 included studies. Thirty-four (60.7%) were from high-income countries (HIC) while 22 (39.3%) were from low- and middle-income countries (LMIC). Most (33, 58.9%) had quantitative designs and were published after 2010.

For peer review only - http://bmjopen.bmj.com/site/about/guidelines.xhtml

All but three included data from a single country; 47 (83.9%) included the perspective of doctors, 12 (21.4%) of midwives, nurses or doulas, 5 (8.9%) of service users and 1 (1.8%) of lawyers. Study details including behavioural drivers reported for each of the 56 studies are presented in Supplementary file 2.

Defining and measuring fear of litigation

There was marked heterogeneity in defining or describing FoL in the context of MoB decision-making. Other terms used to imply FoL included liability concerns (18, 28-30), medico-legal fears (9, 12, 31, 32), and defensive medicine (10, 13, 14, 17, 29, 33-36). The only tool we found was a risk attitude and fear index developed by Fuglenes, to measure the impact of perceived risk of complaints and malpractice litigation on providers' choice of MoB (12).

Ten studies explored FoL impact on MoB decision-making beyond providers self-report. The proxy measures used to explore FoL included changes to malpractice insurance premiums (35-39), previous experience with litigation (15, 18, 40, 41), and changes to liability laws (18, 42).

Drivers of fear of litigation

Using the WHO principles, we organized the drivers under three domains: 1) Cognitive or intrapersonal drivers; 2) Socio-cultural drivers; and 3) Environmental drivers (Fig 2). For each of the three domains, Table 2 shows the key drivers for FoL identified in this review, their definitions, and frequency and countries from which the findings drive. Findings for each domain are summarized below.

1. Cognitive drivers

For peer review only - http://bmjopen.bmj.com/site/about/guidelines.xhtml

1. 1 Availability bias (in relation to experience with litigation)

The impact of malpractice claims was discussed in 18 studies. In these accounts, personal experience of litigation was not essential for inciting FoL, rather ‘hearing’ about or knowing someone who was sued for malpractice could be enough (14, 32, 40). Malpractice suits can be highly publicized events in the media and within the healthcare system (14, 31) even though their impact is not always tied to adverse judicial outcomes for the provider. The mere thought of the experience of being sued due to failure or delay in performing CS, or the hypothesised impact on providers’ time and reputation alone influenced decision-making towards a CS (43, 44).

Some authors have quantified the impact of malpractice claims on defensive medicine practices among providers beyond personal experience showing mixed results. Brown et al found that one standard deviation increase in lawsuits was associated with a 1.073 increase in risk of performing CS in United States (15). Also in the United States, Cheng et al reported that clinicians who were sued were more likely to recommend CS than those who had not gone through this experience (17.2 % vs 11.3% respectively, P= 0.008) (35) while Dranove and Watanabe found a modest impact of litigation experience on CS rates of sued obstetricians and their immediate hospital colleagues (40).

1.2 Ambiguity aversion: CS “just in case”, avoid “worst case scenario”

Ambiguity, in the form of uncertainty of length and outcome of vaginal birth (VB), was a justification for preferring CS to avoid litigation consequences in 16 studies (9, 10, 28, 30, 44-55). This tendency to avoid ambiguity is what leads professionals to perform CS, especially in the context of caesarean delivery on maternal request (CDMR) (53), or vaginal birth after CS (VBAC), or risk of uterine rupture (30, 52).

This ambiguity also may lead providers to shift the responsibility of decision making to the women and their families, rather than giving their professional opinion, as a means of

For peer review only - http://bmjopen.bmj.com/site/about/guidelines.xhtml

avoiding litigation in case of complications; a CS becomes a sort of insurance policy (53). Some authors note that this behaviour is reinforced by the fact that women and their families are increasingly perceiving adverse events in maternity care as unacceptable (9, 45, 52).

1.3 Relative risk bias and beliefs around safety

Seven studies explicitly discussed providers' beliefs around the relative safety of CS (34, 39, 45, 47, 56-58). The inclination to perform CS to avoid litigation risk can be motivated by providers' belief that CS is a safer option than VB, and that CS guarantees the best outcome for mother and child (59). This belief can supersede training, guidelines, or statistical evidence demonstrating the opposite (60).

In LMIC, this belief is further reaffirmed by the reduction in maternal morbidity and mortality due to the introduction –and framing– of CS as a lifesaving intervention in these countries (56). Guidelines and research outlining the benefits of VB could be dismissed by providers in LMIC as perceived not reflecting the realities of their experiences (46).

Even when providers acknowledge the comparative disadvantages of CS to mother and child in the long-term, they may not share this information with their clients (46). As a consequence, the perceived short-term disadvantages of VB are exaggerated or overstated, making VB the less desirable option for providers in light of the risk of litigation, even when VB is objectively safer for women and babies (28).

1.4 Commission bias

Six studies identified drivers that described a tendency to perform CS even if medically unnecessary, just to "do something" rather than wait for uncertain labour events or outcomes (10, 31, 34, 46, 50, 60). With rising rates of CS and lower tolerance for negative maternity care outcomes, providers find waiting for VB challenging, especially if labour is not progressing as expected (46).

For peer review only - http://bmjopen.bmj.com/site/about/guidelines.xhtml

Such findings can be categorized as commission bias in maternity care despite evidence that performing CS without medical indication does not reduce risk of lawsuits (61). It contrasts with “omission bias” as described by Blumenthal et al. in other medical specialties (20), where harmful commissions are usually judged harsher than omissions. In case of complications, maternity care providers perceived that they are more likely to be sued if they failed to perform a CS or delayed it, but unlikely to be sued for performing an unnecessary CS. A study surveying 403 obstetricians in Brazil reported that 67.5% believed VB poses a higher risk of litigation in case of complications, while only 0.5% found that CS had a higher litigation risk (10). A Turkish study found that the perception of VB as a litigation risk was reinforced by wide media coverage of legal cases focusing on delay or failure to perform a CS (31).

1.5 Loss/gain framing or loss aversion bias

Four studies discussed the substantial direct and indirect costs associated with litigation, regardless of the litigation outcome, which underpinned the loss aversion bias pervasiveness in maternity care. Losing a case might result in losing medical licensure, and/or paying large settlements (10, 31, 43). Moreover, regardless of the legal outcome, providers might have to pay higher premiums for malpractice insurance (36), face negative impacts on their reputation (38, 40), and suffer the time and psychological burden of the litigation process itself (35, 43).

2. Social and cultural drivers

2.1 Patient pressure

Nineteen studies explicitly examined FoL as a driver for providers to agree to a CS based on maternal request and/or pressure by mothers and families (28, 43, 48, 50, 53, 54, 56-59, 62-70).

For peer review only - http://bmjopen.bmj.com/site/about/guidelines.xhtml

Providers' attitudes towards CDMR varied between some providers believing women have a right to choose how they give birth, and others asserting that medical necessity supersedes service users' preferences (57, 68). A study conducted in Italy aimed to explore the perceptions of provider legal liability in case of CDMR among obstetricians, midwives, lawyers and patients (66). On average, patients and lawyers rated concession to CDMR as highly appropriate, in contrast to providers who found it inappropriate. Patients and lawyers also rated judicial responsibility of the providers for complications after a CDMR lower than after a VB, suggesting that provider's compliance with maternal choice can be the deciding factor in litigation process. Providers reported that respecting maternal wish for CS is more likely to help avoid litigation in case of complications.

Women's socioeconomic status and education also impacted the providers' perception of their likelihood to be sued in case of complications. Brazilian providers described how more educated women have a higher expectation of care and intolerance to complications during birth, both of which lead to increased risk of litigation if their demands for a CS are not met (62).

2.2 Social Norms: CS as the new 'normal birth'

Eight studies reported providers' concerns that CS is becoming a social norm among women and relatives, since it is widely regarded as safer and 'more modern' than VB (10, 28, 34, 43, 47, 51, 62, 69). In two studies in China and Brazil, providers claimed that CS is expected by service users and families, posing substantial pressure on providers (10, 51). It has been observed that, as countries become more economically stable, families have less children and acceptance or tolerance of adverse events during labour is reduced (14, 43, 51). In these contexts where CS may be seen as the norm, the perception of VB by providers as riskier increases its perceived litigation risk and decreases a provider's ability to defend their performance following a complication (28, 62).

For peer review only - http://bmjopen.bmj.com/site/about/guidelines.xhtml

2.3 Blame Culture

Six studies explicitly addressed the effect of blame culture on FoL in relation to CS choice (13, 28, 46, 49, 65, 69), in addition to four studies implicitly discussing effects of blame culture (30, 52, 53, 71). This culture can result in hesitancy to even suggest VB as an option for fear of blame in case of complications (53).

In countries where providers expressed less fear of being blamed, they also reported less inclination to perform a CS. In a study on CS decision-making in Sweden, midwives expressed that FoL is not a concern because the medical and legal environment do not foster placing blame, which improves transparency and empowers midwife-led care. Similarly, a study in multiple European countries reported that participants attributed the low rates of VBAC in their countries (Ireland and Italy) to blame culture, in comparison to higher rates in Sweden (69). A qualitative study of three countries with high VBAC rates (Finland, Sweden, and the Netherlands) showed that providers in the Netherlands were concerned with their decline in VBAC rates due to increased risk of litigation and blame in case of complications (71).

For peer review only

3. Environmental drivers

The third principle encompasses the external factors that enable or block a behaviour, including the legal environment, organizational culture, medical bodies policies, guidance, media, and other larger environmental or structural factors that might influence the perceptions of the providers and their decisions (Table 2).

3.1 The legal environment

The legal environment and how it influences maternity care was the focus of 24 studies (9, 10, 17, 18, 29-32, 34, 35, 37, 39, 42, 43, 45, 46, 49, 51, 57, 59, 72-75). While laws, legal

For peer review only - http://bmjopen.bmj.com/site/about/guidelines.xhtml

norms and procedures differ by context, we identified common themes across different studies.

The first theme depicted the perceived randomness of legal processes due to the absence of specialized courts, or of a clear legal framework for medical complaints (10, 43), or in some cases even a malpractice law (10, 30, 31). These gaps resulted in a perception of lack of fair and specialized legal processes and reinforced providers' perceptions of their vulnerability in case of adverse event, contributing to FoL.

In addition, reliance on expert witnesses in court to testify regarding fault or negligence is a driver for fear of litigation because of the perceived lack of rigour and criteria for who could be considered an expert to assess medical negligence or malpractice or the degree to which they understand the variability in medical opinions (12). In a study in Iran, providers expressed discontent with the lack of training or knowledge of expert witnesses whom they perceived as chosen based on personal relationships, and as having no interest in examining the causes of adverse events, but who rather relied on emotional criteria (43).

The second theme illustrates the lack of clear demarcation within legal systems between malpractice and unavoidable adverse events (45). Providers in some of the included studies perceived that judges, jurors, and expert witnesses assign fault based on empathy with the families' suffering a loss or disability following birth, rather than looking at whether or not this negative outcome was a result of negligence (30). This perception could be reinforced in contexts where limited social or financial support to families and children with disabilities existed, and hence the legal environment seeks to place blame as a way to help the family with the resulting settlement (8).

Once again, this legal environment results in providers considering CS a safer option. If sued after a VB, providers believe they will need to justify not having conducted a CS and whether

For peer review only - http://bmjopen.bmj.com/site/about/guidelines.xhtml

a CS could have prevented the negative outcome (46, 66). On the other hand, performing a CS when not medically necessary is not recognized as malpractice (12, 46, 66). In addition, depending on the legal context, providers can be at risk of liability for fault even years after the birth. This adds to providers' long-term perceived risk of not performing a CS (43).

As more cases are settled in favour of families based on adverse outcomes, and more service users are aware of their right to sue if the birth does not go according to their expectations, the legal and financial incentives for litigation are mounting (35). In five studies providers claimed that the settlements themselves have become an incentive for more litigation, and for providers to avoid VB for FoL (10, 34, 38, 43, 51).

3.2 Malpractice insurance premiums

Malpractice insurance premiums and their relationship to FoL was discussed in nine studies (31, 35-37, 39, 45, 48, 75, 76). A study in the US, found that the likelihood for having a CS increased, and the likelihood for a VD or an instrumental VB decreased, in states where insurance premiums for malpractice exceeded 100,000 USD compared to states where insurance premiums were below 50,000 USD. Inversely, another study found that higher malpractice insurance premiums were not positively associated with recommending CS, but rather that providers who did not know how much they paid for malpractice insurance premiums were less likely to recommend CS, suggesting a possible lesser preoccupation with malpractice risk (35).

3.3 Medical and professional environment

Seven studies discussed medical and professional environments in which providers reported lack of or little protection afforded to them by medical bodies and authorities (14, 28, 30, 34, 40, 49, 76). Medical providers report feeling discouraged or 'demoralized' for having to face these risks without the backing of their colleagues and scientific authorities for their clinical decisions in societies that are becoming increasingly litigious (34).

For peer review only - http://bmjopen.bmj.com/site/about/guidelines.xhtml

3.4 Mass and social media

Media was mentioned as an influential actor in motivating FoL associated with MoB in five studies (10, 14, 31, 59, 62). The sensationalizing of malpractice claims incited fear and frustration among providers. A study in Turkey found that all included providers attested to practicing defensive medicine and being affected by recent high profile malpractice cases in media which resulted in large settlements (31). This media effect, whether traditional or social media, went beyond fearing the risk of litigation, to having a serious impact on providers' mental health, reputation and their approach to their profession (10).

Discussion

This scoping review gathered the evidence available on drivers of FoL when deciding for MoB from the perspective of behavioural theory. The findings highlight the complexity of FoL as a construct that contributes to rising rates of CS across the world. Our use of behavioural principles as a framework for examining drivers FoL at different levels, aided in understanding how they can interact to influence providers' behaviours and choices (Fig 2). This behavioural lens also lent itself to identifying cognitive influences and biases that, while not always explicitly mentioned in the literature, were interpreted through providers' accounts. Identifying such bias could inform the design of behavioural interventions aimed at influencing the driver of FOL.

This review exposed the underlying factors –and the domains they pertain to– which build FoL motivating providers' decision to perform a CS beyond medical need. While our focus was litigation, it is hard to extricate these fears from perceptions of relative safety of CS, fears of disrepute, and consequences of the process of litigation itself rather than its outcome. This more nuanced understanding of fear as a driver for CS is crucial to address unnecessary surgical births globally.

For peer review only - http://bmjopen.bmj.com/site/about/guidelines.xhtml

The review exposed the need for a consensus around measuring FoL in maternity care, given the diversity of proxies used such as malpractice premiums or experience of litigation. Most studies relied on providers self-reported subjective definition, understanding and perception of FoL, without a standardized scale or comparison of actual litigation risk and outcomes. Relying on self-report poses a challenge to reliably quantify and compare the magnitude or burden of FoL, particularly given the multitude of other factors driving the increasing CS rates and which may be beyond the providers' control (77, 78). Using more complex measures such as a 'fear index' as composite measures of FoL as proposed by Fuglenes et al. (12) could provide some value if used in conjunction with other objective measures of litigation risk such as malpractice claims.

Healthcare providers have to rapidly integrate and interpret a complex range of social, psychological, clinical and emotional data relating to both the person they are caring for and the wider context they are working in, in continuously changing situations where often little time is available to make decisions. In maternity care, this is intensified by the fact that the care provider is undertaking this task for both mother and foetus during both pregnancy, labour, and birth, and balancing the sometimes-differing needs of both. Despite existing guidelines, fast decisions require intuitive thinking processes, that jump to the most likely interpretation of and solution to a problem based on patterns of expert knowledge rather than following sequential rules of rationality (79). This review suggests that, in terms of decision for MoB, emotional responses and primarily fear, can be driven by cognitive biases including rational and irrational beliefs regarding safety and risk. These beliefs can supersede training, guidelines or statistical evidence and are deeply entrenched in socio-cultural structures (8, 20). The influence of sociocultural and environmental factors encompasses multiple stakeholders including women, families, health professionals, healthcare organizations and

For peer review only - http://bmjopen.bmj.com/site/about/guidelines.xhtml

legal systems. Hence multi-level approaches beyond knowledge-based intervention and training targeting provides are required to address FoL (80).

This scoping review unveiled the diversity of drivers behind fear as experienced by providers, the domains to which these drivers belong as, the actual risk of litigation and how it manifests in medical practice. Further contextual studies are warranted for in-depth understanding of local dynamics. However, the range of contexts included in the review suggest that underlying concepts revealed in the data synthesis are potentially transferable between quite different contexts.

Our review also highlights the close relationship and interaction between FoL and shifts in norms, particularly through the role and power of media either mainstream or social. Our findings suggest that trial-by-media may be regarded as having a very real immediate influence while the consequences of any litigation process may take years and are ambiguous. The increasing tendency for sensationalist media coverage, and 'click-bait' reinforces this trend (81). Media may also feed into the public perception that CS is the safest and most modern option, and that women should have a choice of elective CS, while downplaying the need to inform people about the risks associated with this choice (as 'fear mongering'). Media pressures may also act to divorce the sense of entitlement to a right to choose from the personal responsibility to accept the consequences for such choices (82). This means that any adverse outcome, even if resulting from personal choices, is deemed to be the fault of someone else, and thus ripe for a litigation case. Further research into how these fears and factors operate and reinforce each other is needed.

Our focus on behavioural drivers for providers means we did not include perspectives of service users and their families, which may be quite different. Although accounts of providers are necessary to understand drivers of FoL, they do not represent an objective measure of the

For peer review only

For peer review only - http://bmjopen.bmj.com/site/about/guidelines.xhtml

strength or frequency of the phenomena described. For example, while 20 of the included studies discuss CDMR as an important driver of increasing CS rates, global rates of CDMR show great variability across regions (0.2%-42%), with upper middle income countries having 11 times the rate of CDMR compared to high income countries (83).

Strengths and limitations

To our knowledge, this is the first review to systematically explore global accounts of FoL and its behavioural drivers in detail providing a broad overview of the evidence on this multidisciplinary topic and identifying research gaps. The use of the WHO behavioural principles enabled us to classify behavioural drivers within specific domains offering clearer pathways for the design of interventions and policies.

Our review has some limitations. We might have overlooked evidence from grey-literature and other informal publications. However, we found and presented data from a range of high-, middle-, and low-income countries. Our classification of cognitive influences on FoL relied on commonly used behavioural constructs and terminologies in medical decision-making literature (8, 20). These constructs may have not been explicitly used by the authors of the studies included to classify their findings, or by participants to describe their own rationale for MoB decision-making. In order to avoid forcing false categories upon the data, we were very conservative in how we classified findings and explicitly sought data that disconfirmed prior assumptions. This was facilitated by the fact that a few distinct themes resonated in the articles across settings. On the other hand, local socio-cultural and environmental influences were often explicitly mentioned in the reviewed articles, and in some cases, were the focus of the study. Although our synthesis showed consistent themes across settings, any initiatives or strategies to address FoL need to be tailored to the specific local context to address the particularities of legislative and health systems and consider how behaviour change

For peer review only

For peer review only - http://bmjopen.bmj.com/site/about/guidelines.xhtml

interventions can be operationalised locally. Lastly, while we were interested in understanding the drivers of medically unnecessary CS, the reviewed literature often did not differentiate between necessary and unnecessary CS, or document the rates of unnecessary CS. Hence, we have approached our study of drivers of FoL in relation to "increasing use of CS" rather than "increasing rates of unnecessary CS".

Conclusion

FoL as a driver for rising CS rates is the result of a complex interaction between cognitive, social, and environment factors. FoL may be generated less by the actual risk of litigation and more by how the providers perceive this risk and its potential consequences of the process itself independently of the actual legal outcome. Though some of the FoL environmental drivers might vary across populations and settings, more can be transferable across different contexts. These include cognitive bias that favour CS choice, social pressure from service users, and prevailing norms such growing intolerance to complications and uncertainty, legal and medical practice environments, and experience with litigation. Behavioural interventions addressing these drivers are likely to be crucial to address FoL as part of strategies aimed at reducing CS rates. A critical first step towards evaluating tools for effective behavioural change in this area would be agreement on a coherent definition and method of measurement of FoL in relation to MoB decision-making.

For peer review only - http://bmjopen.bmj.com/site/about/guidelines.xhtml

Table 1. Main characteristics of 56 studies included in the scoping review.

Characteristic	N of studies (n=56)	References
Economic category (World Bank classification)		
Low-, lower-, or middle-income countries	22	(9, 10, 13, 28, 29, 31, 32, 39, 43, 45-47, 49-51, 55, 56, 58, 60, 62, 64, 70)
High income	34	(12, 14, 15, 17, 18, 30, 34-38, 40-42, 44, 48, 52-54, 57, 59, 63, 65, 66, 68, 69, 71-76, 84, 85)
Study design*		
Quantitative	33	(9, 10, 12-15, 17, 18, 29-32, 35-38, 40-42, 45, 55, 57, 58, 63, 65, 66, 68, 72, 73, 75, 76, 84, 85)
Qualitative	17	(28, 34, 43, 44, 46-50, 52-54, 60, 64, 69, 71, 74)
Mixed methods	6	(51, 56, 59, 62, 70)
Year of publication		
2001-2010	17	(12, 15, 37, 38, 40, 47, 48, 52, 53, 55, 59, 62, 63, 68, 84-86)
2011 to 2022	39	(9, 10, 13, 14, 17, 18, 28-32, 34-36, 41-46, 49-51, 54, 56, 57, 60, 64, 66, 69-76)
Number of countries		
1	53	(9, 10, 12-15, 18, 28-32, 34-38, 40-57, 59, 60, 62-64, 66, 68, 70, 72-76, 84, 85)
>1	3	(65, 69, 71)
Perspectives included *		
Doctors	47	(9, 10, 12-15, 17, 28, 30-32, 34, 35, 39, 43-60, 62-66, 68-76, 84, 85)
Midwives, nurses and doulas	12	(30, 34, 39, 44, 49, 52, 54, 58, 62, 66, 74, 76)
Service users (pregnant women and their families)	5	(44, 49, 54, 62, 66)
Administrators and health service decision makers	2	(28, 44)
Lawyers	1	(66)
Other (including records)	9	(18, 29, 36-38, 40-42, 44, 70)

* Articles can include multiple perspectives

For peer review only

Table 2: Behavioural drivers of fear of litigation (FoL) under the three domains of the WHO principles, definitions, and frequency and countries from which the findings derive.

Domain/ Construct	Definition	Frequency and citations	Countries
**Cognitive or intrapersonal drivers** (Heuristics and cognitive biases): Heuristics as a decision-making strategy, it relies on a few relevant predictors, ignoring others (87). These shortcuts are helpful in some cases but may lead to cognitive biases i.e. a systematic error in judgement when processing information regarding the world around us that eases the cognitive burden of judgement and decision making (20, 88, 89).			
**1.1 Availability bias (in relation to experience with litigation)**	"To make judgments of likelihood or frequency based on ease of recall rather than on actual probabilities"(20) and pertains to both direct experience, or vicarious through others	18 (10, 12-15, 18, 31, 32, 35, 38, 40, 41, 43, 44, 59, 76, 84, 85)	Brazil, Iran, Israel, Norway, Sudan, Turkey, UK, and US
**1.2 Ambiguity aversion: CS "just in case", avoid "worst case scenario"**	The tendency to prefer known or certain probabilities over uncertainty regardless of the potential benefits (20).	16 (9, 10, 28, 30, 44-55)	Brazil, Canada, China, Iran, Kenya, Nicaragua, Paraguay, Sweden, Taiwan, Turkey, UK, US,
**1.3 Relative risk bias and beliefs around safety**	"Stronger inclination to [a birth mode] when presented with the relative risk than when presented with the same [information] described in terms of the absolute risk" (90)	7 (17, 34, 39, 45, 47, 56, 57)	Argentina, Bangladesh, Brazil, Italy, Romania, Spain, and Turkey
**1.4 Commission bias**	When presented with an unclear decision, medical providers could have a higher tendency towards action versus inaction (91).	6 (10, 31, 34, 46, 50, 60)	Brazil, India, Iran, Paraguay, Spain, and Turkey
**1.5 Loss/gain framing or loss aversion bias**	Losses are often perceived as looming larger than corresponding gains (92)	4 (10, 28, 34, 50)	Brazil, Nicaragua, Paraguay, Spain
**2.Social and cultural drivers**			
**2.1 Patient pressure**	Pregnant women, or their families exerting pressure on providers to perform Caesarean delivery on Maternal Request (CDMR), for nonmedical reasons	19 (28, 43, 48, 50, 53, 54, 56-59, 62-70).	Bangladesh, Brazil, Canada, France, Germany, India, Iran, Italy, Luxembourg, Netherlands, Nicaragua, Paraguay, Romania, Spain, Sweden, Taiwan, UK, US,
**2.2 Social norms: CS as the new 'normal birth'**	Implicit or explicit rules used by a group and which determine values, beliefs, attitudes and behaviours (93). These norms can be descriptive, i.e. what people observe as the typical overt behaviour of others, or injunctive norms, i.e. inference of what is expected of an individual by others(94).	8 (10, 28, 34, 43, 47, 51, 62, 69)	Brazil, China, Germany, Iran, Ireland, Italy, Nicaragua, Spain

For peer review only - http://bmjopen.bmj.com/site/about/guidelines.xhtml

**2.3 Blame culture**	A culture that does not tolerate error can be a driver to medical excesses, and negative impacts on healthcare quality and patient safety (95, 96).	10 (13, 28, 30, 46, 49, 52, 53, 65, 69, 71)	Iran, Ireland, Italy, Netherland, Nicaragua. Sudan, UK, US<br><br>Sweden (mention of absence of blame culture)
**3. Environmental drivers:** Any factor external to the individual that could impact the behaviour. This could include physical environment, or social environment (e.g. medical administrators, peers, patients and their families, etc)			
**3.1 Legal environment**	The legal framework within which providers practice and are faced with liability. This could include tort laws, court systems, admissible evidence, and expert witnesses. This also covers legal mediation, negotiation and settlements outside the court systems	24 (9, 10, 17, 18, 29-32, 34, 35, 37, 39, 42, 43, 45, 46, 49, 51, 57, 59, 72-75)	Brazil, China, Iran, Italy, Romania, Spain, Sweden, Turkey, UK, US,
**3.2 Malpractice insurance premiums**	The existing insurance mechanisms against malpractice including degree of coverage and premiums	9 (31, 35-37, 39, 45, 48, 75, 76)	Argentina, Canada, Turkey, US,
**3.3 Medical and professional environment**	The medical system which organizes obstetric practices through medical guidelines, obstetric organizations, or health facility administration	7 (14, 28, 30, 34, 40, 49, 76)	Iran, Israel, Nicaragua, Spain, US,
**3.4 Mass and social media**	This including social and mass media	5 (10, 14, 31, 59, 62)	Brazil, Israel, Turkey, US

Acknowledgements

With thanks to Jane Gibbon, Sarah Cordey and Louise Hunt.

Contributorship statement

All authors included on a paper fulfil the criteria of authorship. SE, EA, SD and APB conceived and plan the review. SE and APB screened the studies. SE conducted data extraction and analyses, SE wrote the first draft of the manuscript with support from EA and

For peer review only - http://bmjopen.bmj.com/site/about/guidelines.xhtml

APB. SD, JE, SM, BRS, MRT made substantial contributions to interpretation, and critically reviewed the manuscript for important intellectual content.

Competing interest

The authors have declared that no competing interests exist.

Funding

This scoping review was funded by the Behavioural Insights Unit at World Health Organization, the UNDP-UNFPA-UNICEF-WHO-World Bank Special Programme of Research, Development and Research Training in Human Reproduction (HRP), a cosponsored program executed by the World Health Organization (WHO) in the Department of Sexual and Reproductive Health and Research (SRH) and the Gates Grand Challenges 2020: Improving Access to and Use of Safe and Appropriate Cesarean Section (INV-023309). The review was conceived to inform the project Re-JUDGE (https://gcgh.grandchallenges.org/grant/re-judge-reducing-rates-non-medically-indicated-cesarean-sections-through-open-access-multi).

Data sharing statement

All data relevant to the study is included in the article or is available in the supplementary files.

Ethics approval statement

This is a scoping review. No ethics approval was needed.

For peer review only - http://bmjopen.bmj.com/site/about/guidelines.xhtml

References

1. Betran AP, Temmerman M, Kingdon C, Mohiddin A, Opiyo N, Torloni MR, et al. Interventions to reduce unnecessary caesarean sections in healthy women and babies. Lancet. 2018;392(10155):1358-68.
2. Gibbons L, Belizan JM, Lauer JA, Betran AP, Merialdi M, Althabe F. Inequities in the use of cesarean section deliveries in the world. American journal of obstetrics and gynecology. 2012;206(4):331. e1-. e19.
3. Steer PJ, Modi N. Elective caesarean sections--risks to the infant. Lancet. 2009;374(9691):675-6.
4. Victora CG, Barros FC. Beware: unnecessary caesarean sections may be hazardous. Lancet. 2006;367(9525):1796-7.
5. Thirumoorthy T. Understanding Medical Negligence and Litigation–Basic for the Medical Professional. BMA News. 2011:12-3.
6. Grant K, White J, Martin J, Haines T. The costs of risk and fear: a qualitative study of risk conceptualisations in allied health resource allocation decision-making. Health, Risk & Society. 2019;21(7-8):373-89.
7. Domingues AP, Moura P, Vieira DN. Obstetric litigation: the importance of the quality of clinical files and its influence on expertise conclusions. Journal of obstetrics and gynaecology : the journal of the Institute of Obstetrics and Gynaecology. 2015;35(2):146-9.
8. Minkoff H. Fear of litigation and cesarean section rates. Semin Perinatol. 2012;36(5):390-4.
9. Kucuk M. Obstetrician perceptions of the causes of high cesarean delivery rates in Turkey. International journal of gynaecology and obstetrics: the official organ of the International Federation of Gynaecology and Obstetrics. 2017;138(1):100-6.
10. Rudey EL, Leal MDC, Rego G. Defensive medicine and cesarean sections in Brazil. Medicine. 2021;100(1):e24176.
11. Adinma J. Litigations and the Obstetrician in Clinical Practice. Ann Med Health Sci Res. 2016;6(2):74-9.
12. Fuglenes D, Oian P, Kristiansen IS. Obstetricians' choice of cesarean delivery in ambiguous cases: is it influenced by risk attitude or fear of complaints and litigation? Am J Obstet Gynecol. 2009;200(1):48 e1-8.
13. Ali AA, Hummeida ME, Elhassan YA, Nabag WO, Ahmed MA, Adam GK. Concept of defensive medicine and litigation among Sudanese doctors working in obstetrics and gynecology. BMC Med Ethics. 2016;17:12.
14. Asher E, Dvir S, Seidman DS, Greenberg-Dotan S, Kedem A, Sheizaf B, et al. Defensive medicine among obstetricians and gynecologists in tertiary hospitals. PloS one. 2013;8(3):e57108.
15. Brown HS, 3rd. Lawsuit activity, defensive medicine, and small area variation: the case of Cesarean sections revisited. Health economics, policy, and law. 2007;2(Pt 3):285-96.
16. Clark K. Litigation: a threat to obstetric practice? Journal of law and medicine. 2002;9(3):303-22.
17. Fineschi V, Arcangeli M, Di Fazio N, Del Fante Z, Fineschi B, Santoro P, et al. Defensive Medicine in the Management of Cesarean Delivery: A Survey among Italian Physicians. Healthcare (Basel). 2021;9(9).
18. Frakes M. Defensive medicine and obstetric practices. Journal of Empirical Legal Studies. 2012;9(3):457-81.

For peer review only - http://bmjopen.bmj.com/site/about/guidelines.xhtml

19. AlDakhil LO. Obstetric and gynecologic malpractice claims in Saudi Arabia: Incidence and cause. Journal of forensic and legal medicine. 2016;40:8-11.
20. Blumenthal-Barby JS, Krieger H. Cognitive biases and heuristics in medical decision making: a critical review using a systematic search strategy. Medical Decision Making. 2015;35(4):539-57.
21. Technical Note from the WHO Technical Advisory Group (TAG) on behavioural insights and science for health. Principles and steps for applying a behavioural perspective to public health: World Health Organization; 2021 [Available from: https://cdn.who.int/media/docs/default-source/documents/bi-tag-technical-note1_principles-and-steps.pdf?sfvrsn=efdefb39_5&download=true.
22. Peters MD, Godfrey CM, Khalil H, McInerney P, Parker D, Soares CB. Guidance for conducting systematic scoping reviews. JBI Evidence Implementation. 2015;13(3):141-6.
23. Tricco AC, Lillie E, Zarin W, O'Brien KK, Colquhoun H, Levac D, et al. PRISMA extension for scoping reviews (PRISMA-ScR): checklist and explanation. Annals of internal medicine. 2018;169(7):467-73.
24. Erlingsson C, Brysiewicz P. A hands-on guide to doing content analysis. Afr J Emerg Med. 2017;7(3):93-9.
25. Graneheim UH, Lindgren BM, Lundman B. Methodological challenges in qualitative content analysis: A discussion paper. Nurse Educ Today. 2017;56:29-34.
26. Bandura A. The evolution of social cognitive theory. Great minds in management. 2005:9-35.
27. Schunk DH, DiBenedetto MK. Motivation and social cognitive theory. Contemporary Educational Psychology. 2020;60:101832.
28. Colomar M, Cafferata ML, Aleman A, Castellano G, Elorrio EG, Althabe F, et al. Mode of childbirth in low-risk pregnancies: Nicaraguan physicians' viewpoints. Maternal and child health journal. 2014;18(10):2382-92.
29. Chen BK, Yang C-Y. Increased Perception of Malpractice Liability and the Practice of Defensive Medicine. Journal of Empirical Legal Studies. 2014;11(3):446-76.
30. Cox KJ. Providers' perspectives on the vaginal birth after cesarean guidelines in Florida, United States: a qualitative study. BMC Pregnancy Childbirth. 2011;11:72.
31. Kucuk M. Defensive medicine among obstetricians and gynaecologists in Turkey. Journal of obstetrics and gynaecology : the journal of the Institute of Obstetrics and Gynaecology. 2018;38(2):200-5.
32. Samadi S, Gholizadeh N, Shoar N, Shoar S. Attitudes of Obstetricians toward Cesarean Delivery in Challenging Cases. J Obstet Gynaecol India. 2013;63(5):301-5.
33. Ionescu C. Medical malpractice cases spark debate in Romania. Lancet. 2008;371(9611):462-3.
34. Bermudez-Tamayo C, Fernandez Ruiz E, Pastor Moreno G, Maroto-Navarro G, Garcia-Mochon L, Perez-Ramos FJ, et al. Barriers and enablers in the implementation of a program to reduce cesarean deliveries. Reprod Health. 2017;14(1):106.
35. Cheng YW, Snowden JM, Handler SJ, Tager IB, Hubbard AE, Caughey AB. Litigation in obstetrics: does defensive medicine contribute to increases in cesarean delivery? The journal of maternal-fetal & neonatal medicine : the official journal of the European Association of Perinatal Medicine, the Federation of Asia and Oceania Perinatal Societies, the International Society of Perinatal Obstet. 2014;27(16):1668-75.
36. Zwecker P, Azoulay L, Abenhaim HA. Effect of fear of litigation on obstetric care: a nationwide analysis on obstetric practice. American journal of perinatology. 2011;28(4):277-84.

For peer review only - http://bmjopen.bmj.com/site/about/guidelines.xhtml

37. Yang YT, Mello MM, Subramanian SV, Studdert DM. Relationship between malpractice litigation pressure and rates of cesarean section and vaginal birth after cesarean section. Med Care. 2009;47(2):234-42.
38. Gimm GW. The impact of malpractice liability claims on obstetrical practice patterns. Health services research. 2010;45(1):195-211.
39. Perrotta C, Romero M, Sguassero Y, Straw C, Gialdini C, Righetti N, et al. Caesarean birth in public maternities in Argentina: a formative research study on the views of obstetricians, midwives and trainees. BMJ open. 2022;12(1):e053419.
40. Dranove D, Watanabe Y. Influence and Deterrence: How Obstetricians Respond to Litigation against Themselves and Their Colleagues. American Law and Economics Review. 2010;12(1):69-94.
41. Durrance CP, Hankins S. Medical Malpractice Liability Exposure and OB/GYN Physician Delivery Decisions. Health services research. 2018;53(4):2633-50.
42. Esposto AG. Tort reform and caesarean deliveries. Applied Economics Letters. 2012;19(12):1171-4.
43. Yazdizadeh B, Nedjat S, Mohammad K, Rashidian A, Changizi N, Majdzadeh R. Cesarean section rate in Iran, multidimensional approaches for behavioral change of providers: a qualitative study. BMC Health Serv Res. 2011;11(1):159.
44. Munro S, Kornelsen J, Corbett K, Wilcox E, Bansback N, Janssen P. Do Women Have a Choice? Care Providers' and Decision Makers' Perspectives on Barriers to Access of Health Services for Birth after a Previous Cesarean. Birth (Berkeley, Calif). 2017;44(2):153-60.
45. Akpinar F, Kilic F, Ozturk N, Coskun B, Aksar M, Erkaya S. The effect of medical and social conditions on the mode of delivery: a prospective questionnaire-based study applied to 404 Turkish obstetricians. Archives of gynecology and obstetrics. 2021;303(5):1167-74.
46. Bagheri A, Masoudi Alavi N, Abbaszadeh F. Iranian obstetricians' views about the factors that influence pregnant women's choice of delivery method: a qualitative study. Women Birth. 2013;26(1):e45-9.
47. Dias MA, Deslandes SF. [Cesarean sections: risk perception and indication by attending obstetricians in a public maternity hospital in Rio de Janeiro]. Cadernos de saude publica. 2004;20(1):109-16.
48. Chaillet N, Dube E, Dugas M, Francoeur D, Dube J, Gagnon S, et al. Identifying barriers and facilitators towards implementing guidelines to reduce caesarean section rates in Quebec. Bulletin of the World Health Organization. 2007;85(10):791-7.
49. Firoozi M, Tara F, Ahanchian MR, Latifnejad Roudsari R. Health Care System Barriers to Vaginal Birth after Cesarean Section: A Qualitative Study. Iranian journal of nursing and midwifery research. 2020;25(3):202-11.
50. Goldenberg T, Foster J, Andes KL. Driving birth: Cesarean sections and the medicalization of birth in Gran Asunción, Paraguay. Revista Salud Pública del Paraguay. 2016;6(1):8-15.
51. Hellerstein S, Feldman S, Duan T. Survey of Obstetric Care and Cesarean Delivery Rates in Shanghai, China. Birth (Berkeley, Calif). 2016;43(3):193-9.
52. Kamal P, Dixon-Woods M, Kurinczuk JJ, Oppenheimer C, Squire P, Waugh J. Factors influencing repeat caesarean section: qualitative exploratory study of obstetricians' and midwives' accounts. BJOG : an international journal of obstetrics and gynaecology. 2005;112(8):1054-60.
53. Karlstrom A, Engstrom-Olofsson R, Nystedt A, Thomas J, Hildingsson I. Swedish caregivers' attitudes towards caesarean section on maternal request. Women Birth. 2009;22(2):57-63.

For peer review only - http://bmjopen.bmj.com/site/about/guidelines.xhtml

54. Kuan CI. "Suffering twice": the gender politics of cesarean sections in Taiwan. Med Anthropol Q. 2014;28(3):399-418.
55. Koigi-Kamau R, Leting PK, Kiarie JN. Perceptions and practices of vaginal birth after Caesarean section among privately practicing obstetricians in Kenya. East African medical journal. 2005;82(12):631-6.
56. Aminu M, Utz B, Halim A, van den Broek N. Reasons for performing a caesarean section in public hospitals in rural Bangladesh. BMC Pregnancy Childbirth. 2014;14(1):130.
57. Ionescu CA, Dimitriu M, Poenaru E, Banacu M, Furau GO, Navolan D, et al. Defensive caesarean section: A reality and a recommended health care improvement for Romanian obstetrics. Journal of evaluation in clinical practice. 2019;25(1):111-6.
58. Zhao P, Cai Z, Huang A, Liu C, Li H, Yang S, et al. Why is the labor epidural rate low and cesarean delivery rate high? A survey of Chinese perinatal care providers. PloS one. 2021;16(5):e0251345.
59. Savage W, Francome C. British consultants' attitudes to caesareans. Journal of obstetrics and gynaecology : the journal of the Institute of Obstetrics and Gynaecology. 2007;27(4):354-9.
60. Peel A, Bhartia A, Spicer N, Gautham M. 'If I do 10–15 normal deliveries in a month I hardly ever sleep at home.' A qualitative study of health providers' reasons for high rates of caesarean deliveries in private sector maternity care in Delhi, India. BMC Pregnancy and Childbirth. 2018;18(1):470.
61. Kessler DP, Summerton N, Graham JR. Effects of the medical liability system in Australia, the UK, and the USA. Lancet. 2006;368(9531):240-6.
62. Behague DP, Victora CG, Barros FC. Consumer demand for caesarean sections in Brazil: informed decision making, patient choice, or social inequality? A population based birth cohort study linking ethnographic and epidemiological methods. BMJ (Clinical research ed). 2002;324(7343):942-5.
63. Bettes BA, Coleman VH, Zinberg S, Spong CY, Portnoy B, DeVoto E, et al. Cesarean delivery on maternal request: obstetrician-gynecologists' knowledge, perception, and practice patterns. Obstet Gynecol. 2007;109(1):57-66.
64. Doraiswamy S, Billah SM, Karim F, Siraj MS, Buckingham A, Kingdon C. Physician–patient communication in decision-making about Caesarean sections in eight district hospitals in Bangladesh: a mixed-method study. Reproductive health. 2021;18(1):1-14.
65. Habiba M, Kaminski M, Da Fre M, Marsal K, Bleker O, Librero J, et al. Caesarean section on request: a comparison of obstetricians' attitudes in eight European countries. BJOG : an international journal of obstetrics and gynaecology. 2006;113(6):647-56.
66. Indraccolo U, Scutiero G, Matteo M, Indraccolo SR, Greco P. Cesarean section on maternal request: should it be formally prohibited in Italy? Annali dell'Istituto superiore di sanita. 2015;51(2):162-6.
67. Gau ML, Chang CY, Tian SH, Lin KC. Effects of birth ball exercise on pain and self-efficacy during childbirth: a randomised controlled trial in Taiwan. Midwifery. 2011;27(6):e293-300.
68. Kwee A, Cohlen BJ, Kanhai HH, Bruinse HW, Visser GH. Caesarean section on request: a survey in The Netherlands. European journal of obstetrics, gynecology, and reproductive biology. 2004;113(2):186-90.
69. Lundgren I, Healy P, Carroll M, Begley C, Matterne A, Gross MM, et al. Clinicians' views of factors of importance for improving the rate of VBAC (vaginal birth after caesarean section): a study from countries with low VBAC rates. BMC Pregnancy Childbirth. 2016;16(1):350.

For peer review only - http://bmjopen.bmj.com/site/about/guidelines.xhtml

70. Makhija B, Verma D, Mustafa A. A root cause analysis of increasing caesarean section rates in a tertiary care private hospital in North India. International Journal of Reproduction, Contraception, Obstetrics and Gynecology. 2019;8(11):4531.
71. Lundgren I, van Limbeek E, Vehvilainen-Julkunen K, Nilsson C. Clinicians' views of factors of importance for improving the rate of VBAC (vaginal birth after caesarean section): a qualitative study from countries with high VBAC rates. BMC Pregnancy Childbirth. 2015;15(1):196.
72. Deutsch AB, Duncan K, Rajaram L, Salihu HM, Spellacy WN, Belogolovkin V. Cesarean or vaginal delivery for the breech fetus at the threshold of viability: results from a maternal-fetal medicine specialists survey. The journal of maternal-fetal & neonatal medicine : the official journal of the European Association of Perinatal Medicine, the Federation of Asia and Oceania Perinatal Societies, the International Society of Perinatal Obstet. 2011;24(3):475-9.
73. Mushinski D, Zahran S, Frazier A. Physician behaviour, malpractice risk and defensive medicine: an investigation of cesarean deliveries. Health economics, policy, and law. 2021:1-19.
74. Panda S, Daly D, Begley C, Karlström A, Larsson B, Bäck L, et al. Factors influencing decision-making for caesarean section in Sweden – a qualitative study. BMC Pregnancy and Childbirth. 2018;18(1):377.
75. Carrera AM, Sternke EA, Rivera-Viñas JI. A pilot study of the perceptions of actively practicing obstetricians in Puerto Rico: factors that influence decision making in cesarean delivery. Puerto Rico Health Sciences Journal. 2017;36(1).
76. Barger MK, Dunn JT, Bearman S, DeLain M, Gates E. A survey of access to trial of labor in California hospitals in 2012. BMC Pregnancy Childbirth. 2013;13:83.
77. Long Q, Kingdon C, Yang F, Renecle MD, Jahanfar S, Bohren MA, et al. Prevalence of and reasons for women's, family members', and health professionals' preferences for cesarean section in China: A mixed-methods systematic review. PLoS medicine. 2018;15(10):e1002672.
78. Panda S, Begley C, Daly D. Clinicians' views of factors influencing decision-making for caesarean section: A systematic review and metasynthesis of qualitative, quantitative and mixed methods studies. PloS one. 2018;13(7):e0200941.
79. Whelehan DF, Conlon KC, Ridgway PF. Medicine and heuristics: cognitive biases and medical decision-making. Irish Journal of Medical Science (1971 -). 2020;189(4):1477-84.
80. Organization WH. WHO recommendations non-clinical interventions to reduce unnecessary caesarean sections: World Health Organization; 2018.
81. O'Keeffe M, Nickel B, Dakin T, Maher CG, Albarqouni L, McCaffery K, et al. Journalists' views on media coverage of medical tests and overdiagnosis: a qualitative study. BMJ open. 2021;11(6):e043991.
82. Moretto G, Walsh E, Haggard P. Experience of agency and sense of responsibility. Consciousness and cognition. 2011;20(4):1847-54.
83. Begum T, Saif-Ur-Rahman KM, Yaqoot F, Stekelenburg J, Anuradha S, Biswas T, et al. Global incidence of caesarean deliveries on maternal request: a systematic review and meta-regression. BJOG : an international journal of obstetrics and gynaecology. 2021;128(5):798-806.
84. Grant D, McInnes MM. Malpractice experience and the incidence of cesarean delivery: a physician-level longitudinal analysis. Inquiry : a journal of medical care organization, provision and financing. 2004;41(2):170-88.
85. Wells CE. Vaginal birth after cesarean delivery: views from the private practitioner. Semin Perinatol. 2010;34(5):345-50.

For peer review only - http://bmjopen.bmj.com/site/about/guidelines.xhtml

86. Cuttini M, Habiba M, Nilstun T, Donfrancesco S, Garel M, Arnaud C, et al. Patient refusal of emergency cesarean delivery: a study of obstetricians' attitudes in Europe. Obstet Gynecol. 2006;108(5):1121-9.
87. Marewski JN, Gigerenzer G. Heuristic decision making in medicine. Dialogues Clin Neurosci. 2012;14(1):77-89.
88. Gilovich T, Griffin D, Kahneman D. Heuristics and biases: The psychology of intuitive judgment: Cambridge university press; 2002.
89. Sinatra GM, Kienhues D, Hofer BK. Addressing challenges to public understanding of science: Epistemic cognition, motivated reasoning, and conceptual change. Educational Psychologist. 2014;49(2):123-38.
90. Kahneman D, Slovic P, Tversky A. Judgment Under Uncertainty: Heuristics and Biases: Cambridge University Press; 1982. 331-40 p.
91. Cioffi J. Decision making by emergency nurses in triage assessments. Accident and emergency nursing. 1998;6(4):184-91.
92. Abhyankar P, O'Connor DB, Lawton R. The role of message framing in promoting MMR vaccination: evidence of a loss-frame advantage. Psychology, health & medicine. 2008;13(1):1-16.
93. Cotterill S, Powell R, Rhodes S, Brown B, Roberts J, Tang MY, et al. The impact of social norms interventions on clinical behaviour change among health workers: protocol for a systematic review and meta-analysis. Systematic Reviews. 2019;8(1):176.
94. Borsari B, Carey KB. Descriptive and injunctive norms in college drinking: a meta-analytic integration. J Stud Alcohol. 2003;64(3):331-41.
95. Khatri N, Brown GD, Hicks LL. From a blame culture to a just culture in health care. Health care management review. 2009;34(4):312-22.
96. Hoffman JR, Kanzaria HK. Intolerance of error and culture of blame drive medical excess. BMJ (Clinical research ed). 2014;349:g5702.

For peer review only

For peer review only - http://bmjopen.bmj.com/site/about/guidelines.xhtml

Fig 1: Flowchart of process of study identification and inclusion.

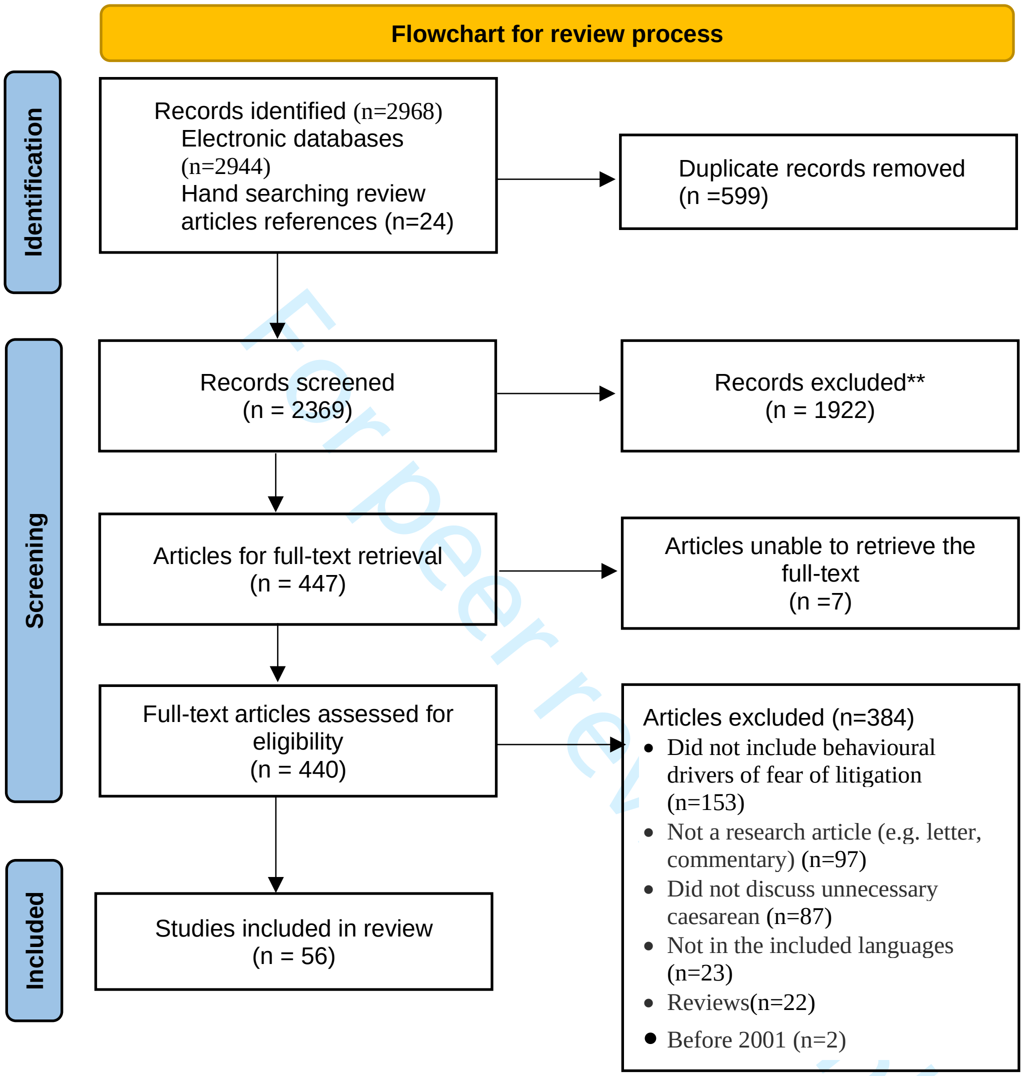

For peer review only - http://bmjopen.bmj.com/site/about/guidelines.xhtml

Fig 1: Conceptual framework for the classification of drivers of fear of litigation under the three domains defined in the WHO principles.

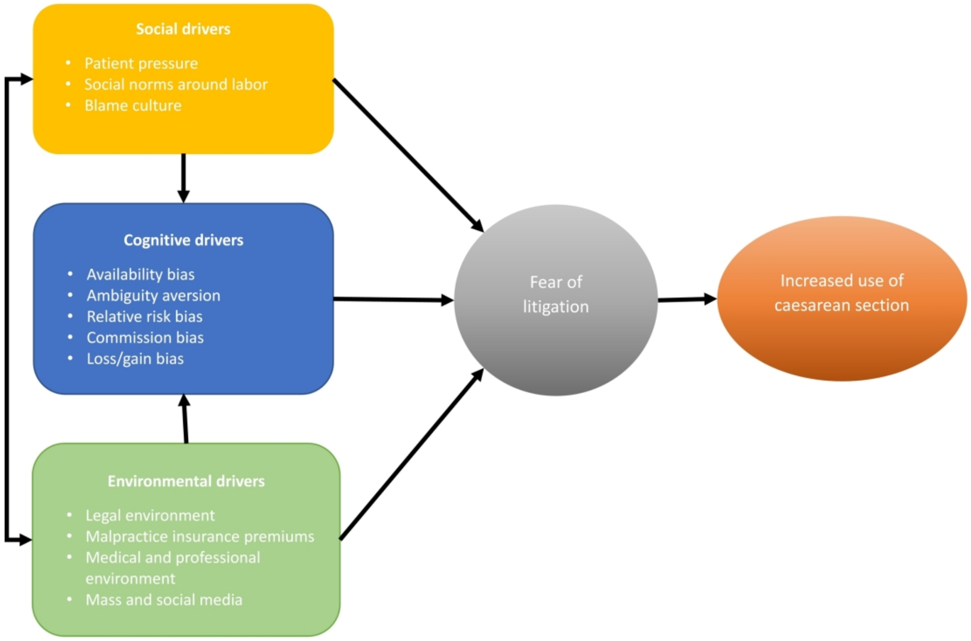

Fig 2: Conceptual framework for the classification of drivers of fear of litigation under the three domains defined in the WHO principles.

228x159mm (300 x 300 DPI)

Supplementary file 1

Search Strategy

PubMed

("OBSTETRICS/LEGISLATION AND JURISPRUDENCE"[MESH] OR "CESAREAN SECTION"[MESH] OR "CESAREAN SECTION"[TIAB] OR "CESAREAN SECTION"[TW] OR "CESAREAN SECTIONS"[TIAB] OR "CESAREAN SECTIONS"[TW] OR "CAESAREAN"[TW] OR "DELIVERY, ABDOMINAL" [TIAB] OR "ABDOMINAL DELIVERIES" [TIAB] OR "DELIVERIES, ABDOMINAL" [TIAB] OR "CAESAREAN SECTION" [TIAB] OR "CAESAREAN SECTIONS" [TIAB] OR "ABDOMINAL DELIVERY" [TIAB] OR "C-SECTION" [TIAB] OR "C SECTION" [TIAB] OR "C-SECTIONS" [TIAB] OR "POSTCESAREAN SECTION"[TIAB])

AND

(LEGIS OR "OBSTETRICS/LEGISLATION AND JURISPRUDENCE"[MESH] OR "JURISPRUDENCE"[MESH] OR "COURT DECISION*" [TIAB] OR "LAW" [TIAB] OR "LAW"[TW] OR "LAWS"[TW] OR "LEGAL*"[TW] OR "LAWY*" [TW] OR "LITIGATION"[TW] OR "LITIGATIONS"[TW] OR "LITIGAT*"[TW] OR "LEGAL CONSEQ*"[TIAB] OR "MEDICAL JURISPRUDENCE" [TW] OR "DEFENSIVE MEDICINE"[MESH] OR "DEFENSIVE PRACTICE*"[TIAB] OR "NEGOTIATING"[MESH] OR "NEGOTIAT*" [TIAB] OR "MEDIATION" [TIAB] OR "MEDIATING" [TIAB] OR "ARBITRATING" [TIAB] OR "ARBITRATION*" [TIAB] OR "CONFLICT RESOLUTION" [TIAB] OR "CONFLICT RESOLUTIONS" [TIAB] OR "RESOLUTION, CONFLICT" [TIAB] OR "MEDICO-LEGAL"[TIAB] OR "VIOLENCE"[TIAB] OR "CASE LAW"[TIAB] OR "LEGAL CASE*"[TIAB] OR "COURT CASE"[TIAB] OR "MALPRACTICE"[MESH] OR "MALPRACTICE"[TIAB] OR "NEGLIGENCE"[TIAB] OR "NEGLIGEN*"[TIAB] OR "MALFEASANCE"[TIAB] OR "MISCONDUCT"[TW] OR "PROFESSIONAL DISCIPLINE"[TW] OR "PATIENT ADVOCACY" [TW] OR "PATIENT RIGHTS"[TW] OR "LIABILITY"[TIAB])

AND

AND PUBYEAR > 2000

Scopus

(TITLE-ABS-KEY(("Caesarean section" OR "Cesarean Section" OR "Caesarean sections" OR "Cesarean Sections" OR "C-section" OR "C-sections" OR "Abdominal delivery" OR "Abdominal deliveries"))

For peer review only - http://bmjopen.bmj.com/site/about/guidelines.xhtml

AND

TITLE-ABS-KEY(("Jurisprudence" OR "Court Decision*" OR "Law" OR "law" OR "laws" OR "legal*" OR "lawy*" OR "litigation" OR "litigations" OR "litigat*" OR "legal conseq*" OR "Medical Jurisprudence" OR "Defensive Medicine" OR "Defensive Practice*" OR "Negotiating" OR "Negotiat*" OR "Mediation" OR "Mediating" OR "Arbitrating" OR "Arbitration" OR "Arbitrations" OR "Conflict Resolution" OR "Conflict Resolutions" OR "medico-legal" OR "Violence" OR "case law" OR "legal case*" OR "court case" OR "Malpractice" OR "malpractice" OR "Negligence" OR "negligen*" OR "malfeasance" OR "misconduct" OR "professional discipline" OR "patient advocacy" OR "patient rights")))

AND PUBYEAR > 2000

WHO Global Index Medicus

("Caesarean section" OR "Cesarean Section" OR "Caesarean sections" OR "Cesarean Sections" OR "C-section" OR "C-sections" OR "Abdominal delivery" OR "Abdominal deliveries")

AND

("Jurisprudence" OR "Court Decision*" OR "Law" OR "law" OR "laws" OR "legal*" OR "lawy*" OR "litigation" OR "litigations" OR "litigat*" OR "legal conseq*" OR "Medical Jurisprudence" OR "Defensive Medicine" OR "Defensive Practice*" OR "Negotiating" OR "Negotiat*" OR "Mediation" OR "Mediating" OR "Arbitrating" OR "Arbitration" OR "Arbitrations" OR "Conflict Resolution" OR "Conflict Resolutions" OR "medico-legal" OR "Violence" OR "case law" OR "legal case*" OR "court case" OR "Malpractice" OR "malpractice" OR "Negligence" OR "negligen*" OR "malfeasance" OR "misconduct" OR "professional discipline" OR "patient advocacy" OR "patient rights")

AND

PUBYEAR > 2000

Detailed inclusion and exclusion criteria

Inclusion criteria

- Focus on caesarean section (not broadly obstetrics).
- Include fear of litigation (broadly defined) in the findings.
- Include analysis of empirical data
- Include peer-reviewed articles, (thesis/dissertation and grey literature for India review only)
- Descriptive or experimental design

For peer review only - http://bmjopen.bmj.com/site/about/guidelines.xhtml

- Can include both quantitative designs/data:
  - *E.g.* cross sectional, cohort, non-randomized controlled trials; program evaluations; interrupted time-series; pre-post designs; pilot studies; etc
  - *N* > *6*
- Qualitative designs/data
  - In-depth interviews; focus groups; key-informant interviews
  - *N* > *1*
- And mixed methods

- Include drivers of fear of litigation (i.e. goes beyond mere mention of its existence).
  - These can include behavioural determinants or drivers.
  - Can include stakeholders or other actors influencing fear of litigation.
- Publication language: English, Spanish, Portuguese, French, or German
- Published between 2001 and 2021

Exclusion criteria

- Case-studies or case-series
- Studies that report on a study or program where main outcomes are NOT related to unnecessary caesarean sections (broadly discuss obstetric malpractice).
- Reports or articles that do not describe original research (e.g. Abstracts, communications, book chapters, news articles, etc)
- Studies with no mention of fear of litigation or its drivers (beyond its presence based on previous literature)
- Pure legal analysis that does not discuss healthcare decision-making
- Review papers with no substantial analysis of fear of litigation (but screened references for more studies)
- Publication language other than English, Spanish, Portuguese, German or French.
- Published before 2001

Variables collected (data-extraction form)

The following variables were extracted in an excel form specifically designed for this scoping review:

- Author, citation
- Year of the study
- Topic/domain
- Country or countries
- Study design
- Data type
- Sample size
- Type of participant
- Participant characteristics

For peer review only - http://bmjopen.bmj.com/site/about/guidelines.xhtml

For peer review only

- Behavioural drivers
- For each behavioural driver, how the behavioural drivers were assessed
- For each behavioural driver, rational or justification for effect on fear of litigation
- For each behavioural driver, other actors in relation with the behavioural driver
- Key limitations
- Key strengths
- Quotes
- Other notes

For peer review only

For peer review only - http://bmjopen.bmj.com/site/about/guidelines.xhtml

Supplementary file 2: Study details including behavioural drivers reported by each study.

Author, year	Country	Method/data type	Participants and sample size	Behavioural drivers for CS
Akpinar, 2021 (1)	Turkey	Survey<br><br>Quantitative data	404 Obstetricians and Gynaecological administrators and practitioners	Ambiguity aversion<br>Beliefs around safety<br>Legal environment<br>Limited coverage of liability insurance
Ali, 2016 (2)	Sudan	Survey<br><br>Quantitative data	117 OBGYN (42.7% consultants, 34.2% registrars and 23.1% specialists)	Availability bias<br>Blame Culture
Aminu et al., 2014 (3)	Bangladesh	Mixed methods (retrospective review of case notes and Key informant interviews with service providers)	18 OBGYN and case notes of 530 women who had CS in 5 Public Hospitals	Patient Pressure<br>Relative risk bias and Beliefs around safety
Asher, 2013 (4)	Israel	Survey<br><br>Quantitative data	117 board certified physicians and residents in OBGYN departments at tertiary hospitals	Availability bias<br>Medical and professional environment<br>Media
Bagheri, 2013 (5)	Iran	Qualitative data	18 OBGYN	Ambiguity aversion<br>Legal environment
Berger, 2012 (6)	United States	Survey<br><br>Quantitative data	Nurses at 243 civilian California birth hospitals	Availability bias<br>Medical and professional environment<br>Malpractice insurance premiums
Bastos-Dias, 2004 (7)	Brazil	Qualitative data	OBGYNs practicing in one public maternity in Rio de Janeiro, caring for women with low education	Beliefs around safety<br>Ambiguity aversion<br>Social norms
Behague, 2002 (8)	Brazil	Mixed methods	80 mothers from a birth cohort of 5304 women who gave birth in 1993 in any of the hospitals in Pelotas, Brazil. As well as 19 clinicians (six	Patient Pressure<br>Media

For peer review only

For peer review only - http://bmjopen.bmj.com/site/about/guidelines.xhtml

			OBGYN, six paediatricians, four general practitioners, and three nurses)	
Bermúdez-Tamayo, 2017 (9)	Spain	Qualitative data	14 OBGYN and 14 nurse-midwives (NMW) who work for Spain's National Healthcare System	Legal environment<br>Commission bias<br>Loss/gain framing or loss aversion bias<br>Social norms<br>Beliefs around safety
Bettes, 2007 (10)	United States	Survey<br><br>Quantitative data	699 OBGYN	Patient pressure
Brown, 2007 (11)	United States	Secondary analysis<br><br>Quantitative data	Hospital discharges data and Insurance Closed Claim File data. Analysis was conducted using a random sample, with replacement of 20,000 births out of the total birth population of 260,292	Availability bias
Carrera, 2017 (12)	United States (Puerto Rico)	Survey<br><br>Quantitative data	62 OBGYN	Patient pressure<br>Legal System<br>Availability bias<br>Malpractice insurance/premiums
Chaillet, Dube (13)	Canada	Qualitative data	10 Focus group discussions (FGDs) and 6 in-depth interviews (IDIs)	Patient pressure<br>Ambiguity aversion<br>Medical and professional environment
Chen and Yang (14)	Taiwan	Panel analysis<br><br>Quantitative data	Random subsamples of population of outpatient and inpatient claim in Taiwan (1997-2005) out of 1 million random individual medical claim histories	Legal environment
Cheng, 2014 (15)	United States	Survey<br><br>Quantitative data	1486 clinicians (general OBGYN, Maternal-Foetal Medicine specialists, family medicine physicians and clinical nurse midwives), who practice obstetrics in the US and who were registered as active members of the American Medical Association (AMA).	Availability bias<br>Malpractice insurance/premiums<br>Legal environment

For peer review only - http://bmjopen.bmj.com/site/about/guidelines.xhtml

Colomar, 2014 (16)	Nicaragua	Qualitative data	4 FGDs, and 17 IDIs with OBGYN and medical decision makers	Patient Pressure<br>Ambiguity aversion<br>Loss/gain framing or loss aversion bias<br>Medical and professional environment
Cox, 2011 (17)	United States	Semi-structured interviews<br><br>Qualitative data	11 obstetricians, 12 midwives, and a hospital administrator (n = 24)	Legal environment<br>Ambiguity aversion<br>Blame Culture
Deutch, 2011 (18)	United States	Survey<br><br>Quantitative data	510 OBGYN (Society for Maternal–Foetal Medicine (SMFM) members)	Legal environment
Doraiswany, 2021 (19)	Bangladesh	IDIs and Labour observations<br><br>Qualitative data	16 physicians and 32 women who delivered by CS	Patient pressure<br>Risk aversion bias (loss gain framing)
Dranove, 2010 (20)	United States	Quantitative record analysis	Using patient-level hospital discharge data and matching physician license of presiding OBGYN to data of closed medical malpractice claims. Examined data of 1539 unique physicians who carried out 98% of the included birth records.	Availability bias<br>Medical and professional environment
Durrance, 2018(21)	United States	Quantitative data	Around 2 million hospital deliveries linked to physician malpractice claim histories (around 2300 doctors) between 1994 and 2010	Availability bias
Esposto, 2012 (22)	United States	Quantitative data	Panel data of CS rates in three years (1987, 1990 and 1993) in 50 states	Legal System (tort reform)
Fineschi, 2021 (17)	Italy	Quantitative data	168 OBGYN	Legal system<br>Patient pressure<br>Relative risk bias and beliefs around safety
Firoozi et al, 2020 (23)	Iran	IDIs and an FGD<br><br>Qualitative data	26 IDIs with maternity care providers (OBGYN and Midwives) and mothers with prior CS	Legal environment<br>Ambiguity aversion<br>Blame culture

For peer review only - http://bmjopen.bmj.com/site/about/guidelines.xhtml

Frakes, 2012 (24)	United States	Secondary quantitative data	Using data on physician behaviour from the 1979–2005 National Hospital Discharge Surveys (NHDS),	Availability bias (Experience with litigation)<br>Legal environment (tort reform)
Fuglenes, 2009 (25)	Norway	Survey<br><br>Quantitative data	716 OBGYN	Perceived risk of litigation (Fear Index)
Gimm, 2010 (26)	United States	Secondary data<br><br>Panel Analysis	Secondary data from the 1992–2000 Florida Hospital Inpatient Discharge File, the Florida Medical Professional Liability Insurance Claims File, and the American Medical Association's Master File on physician characteristics.	Availability bias (Experience with litigation)
Goldenberg, 2016 (27)	Paraguay	IDIs<br><br>Qualitative data	10 OBGYN	Commission bias<br>Loss/gain framing or loss aversion bias<br>Ambiguity aversion<br>Patient pressure
Grant, 2004 (28)	United States	Panel Analysis<br><br>Quantitative data	835 obstetricians who performed at least 10 births in each year and had all the information on physician characteristics	Availability bias
Habiba, 2006 (29)	Luxembourg, Netherlands and Sweden, France, Germany, Italy, Spain and UK	self-administered questionnaire<br><br>Quantitative data	One hundred and five units Neonatal Intensive Care Unit (NICU) associated maternity units, and 1530 obstetricians participated in the study in eight European countries.	Patient pressure
Hellerstein, 2016 (30)	China	Survey and qualitative observations<br><br>Mixed methods data	Descriptive structured survey of obstetric services with standardized interview with obstetricians and qualitative observation of obstetric services in six different Shanghai obstetric facilities	Patient (and family) pressures<br>Ambiguity aversion<br>Social norms regarding birth

For peer review only - http://bmjopen.bmj.com/site/about/guidelines.xhtml

.				
Indraccolo, 2015 (31)	Italy	Survey<br>Quantitative data	36 OBGYNs, 42 midwives, 22 lawyers and 25 patients	Patient pressure
Ionsecu, 2018 (32)	Romania	Survey<br>Quantitative data	73 OBGYNs	Patient pressure<br>Relative risk bias and beliefs around safety<br>Legal environment
Kamal, 2005 (33)	United Kingdom	IDIs<br>Qualitative data	Trainee and consultant obstetricians, and community and hospital-based midwives involved in the care of women in pregnancy and labour in Leicestershire,	Ambiguity aversion<br>Blame Culture
Karlstro¨m, 2009 (34)	Sweden	FGDs<br>Qualitative data	5 FGDs where 16 midwives and 9 obstetricians participated.	Ambiguity aversion<br>Patient pressure
Koigi-Kamau, 2005 (35)	Kenya	Survey<br>Quantitative data	64 obstetricians with individual private practices (i.e., independent of institutional policies) from the first major towns in Kenya – namely Nairobi, Mombasa, Kisumu, Nakuru and Eldoret.	Ambiguity aversion
Kuan (36)	Taiwan	Multiple Qualitative methods	Interviews with eight obstetricians, two nurses, a doula, a midwife, and 34 pregnant women along with document review, and ethnographic observations	Patient pressure<br>Ambiguity aversion
Kucuk (37)	Turkey	Survey<br>Quantitative data	100 obstetricians who responded to the survey.	Legal environment<br>Ambiguity aversion
Kucuk (38)	Turkey	Survey<br>Quantitative data	108 practicing OBGYNs	Availability bias<br>Malpractice insurance/premiums<br>Legal environment

For peer review only - http://bmjopen.bmj.com/site/about/guidelines.xhtml

Kwee, Cohlen (39)	Netherlands	Survey<br><br>Quantitative data	583 OBGYN (80% consultants and 20% were senior registrar	Patient pressure
Lundgren, 2016 (40)	Ireland, Italy and Germany	FGDs<br><br>Qualitative methods	FGDs were held in Ireland, Italy and Germany. In total 71 clinicians participated in nine FGDs.	Social Norms regarding birth<br>Blame culture<br>Patient pressure
Makhija et al., 2019 (41)	India	Mixed methods (birth data and IDIs)	20 consultant OBGYNs	Patient pressure
Munro, 2017 (42)	Canada	Qualitative methods	35 Family physicians, midwives, obstetricians, nurses, anaesthetists, and health service decision makers recruited from 3 rural and 2 urban Canadian communities	Availability bias<br>Ambiguity aversion
Mushiniski, 2021 (43)	United States	Quantitative data		Legal environment
Panda, Daly (44)	Sweden	FGDs<br><br>Qualitative methods	4 FGDs with OBGYNs and Midwives involved in birth decision making	Legal environment
Peel, Bhartia (45)	India	IDs<br><br>Qualitative methods	14 OBGYN and providers involved in maternity care	Relative Risk bias and beliefs around safety<br>Commission bias
Perrotta, 2021 (46)	Argentina	Key informant interviews (KII) and Surveys<br><br>Mixed methods	680 surveys and 26 KIIs of heads of hospital departments, OBGYN, midwives and residents	Legal system<br>Relative Risk bias and beliefs around safety<br>Malpractice insurance/premiums and coverage
Rudey, 2021 (47)	Brazil	Survey<br><br>Quantitative data	403 General obstetricians who are members of premier Brazilian professional associations of gynaecology and obstetrics	Commission bias<br>Loss/gain framing or loss aversion bias<br>Ambiguity aversion<br>Availability bias<br>Legal environment<br>Media

For peer review only - http://bmjopen.bmj.com/site/about/guidelines.xhtml

Samadi, 2013 (48)	Iran	Survey<br><br>Quantitative data	75 OBGYN	Availability bias (Experience with litigation)<br>Legal environment
Savage, 2007 (49)	United Kingdom	Mixed methods	151 OBGYN and clinical directors in OBGYN listed by the royal college of OBGTN in British Isles	Experience with litigation (financial and time drain)<br>Legal environment<br>Patient pressure<br>Media
Wells, 2010 (50)	United States	Survey<br><br>Quantitative data	458 practicing obstetricians	Availability bias (Experience with litigation)
Yang, 2009 (51)	United States	Quantitative data	52 million recorded births state-level longitudinal mixed-effects regression models to examine data from the Natality Detail File on births in the United States (1991–2003)	Malpractice insurance/premiums<br>Legal environment
Yazdizadeh, 2011 (52)	Iran	IDIs and document analysis<br><br>Qualitative data	26 providers from various public and private hospitals responding to the ministry's C-section reduction interventions. Participants included hospital director, obstetricians and midwives.	Availability bias (Experience with litigation)<br>Legal environment (judges' capability, unrealistic expectation from medical professionals, and large settlements)<br>Patient pressure
Zwecker, 2011 (53)	United States	Quantitative record data	890,266 records of women who delivered and malpractice premium data in 37 states in 2006	Malpractice insurance/premiums

For peer review only - http://bmjopen.bmj.com/site/about/guidelines.xhtml

References

1. Akpinar F, Kilic F, Ozturk N, Coskun B, Aksar M, Erkaya S. The effect of medical and social conditions on the mode of delivery: a prospective questionnaire-based study applied to 404 Turkish obstetricians. Archives of gynecology and obstetrics. 2021;303(5):1167-74.
2. Ali AA, Hummeida ME, Elhassan YA, Nabag WO, Ahmed MA, Adam GK. Concept of defensive medicine and litigation among Sudanese doctors working in obstetrics and gynecology. BMC Med Ethics. 2016;17:12.
3. Aminu M, Utz B, Halim A, van den Broek N. Reasons for performing a caesarean section in public hospitals in rural Bangladesh. BMC Pregnancy Childbirth. 2014;14(1):130.
4. Asher E, Dvir S, Seidman DS, Greenberg-Dotan S, Kedem A, Sheizaf B, et al. Defensive medicine among obstetricians and gynecologists in tertiary hospitals. PloS one. 2013;8(3):e57108.
5. Bagheri A, Masoudi Alavi N, Abbaszadeh F. Iranian obstetricians' views about the factors that influence pregnant women's choice of delivery method: a qualitative study. Women Birth. 2013;26(1):e45-9.
6. Barger MK, Dunn JT, Bearman S, DeLain M, Gates E. A survey of access to trial of labor in California hospitals in 2012. BMC Pregnancy Childbirth. 2013;13:83.
7. Dias MA, Deslandes SF. [Cesarean sections: risk perception and indication by attending obstetricians in a public maternity hospital in Rio de Janeiro]. Cadernos de saude publica. 2004;20(1):109-16.
8. Behague DP, Victora CG, Barros FC. Consumer demand for caesarean sections in Brazil: informed decision making, patient choice, or social inequality? A population based birth cohort study linking ethnographic and epidemiological methods. BMJ (Clinical research ed). 2002;324(7343):942-5.
9. Bermudez-Tamayo C, Fernandez Ruiz E, Pastor Moreno G, Maroto-Navarro G, Garcia-Mochon L, Perez-Ramos FJ, et al. Barriers and enablers in the implementation of a program to reduce cesarean deliveries. Reprod Health. 2017;14(1):106.
10. Bettes BA, Coleman VH, Zinberg S, Spong CY, Portnoy B, DeVoto E, et al. Cesarean delivery on maternal request: obstetrician-gynecologists' knowledge, perception, and practice patterns. Obstet Gynecol. 2007;109(1):57-66.
11. Brown HS, 3rd. Lawsuit activity, defensive medicine, and small area variation: the case of Cesarean sections revisited. Health economics, policy, and law. 2007;2(Pt 3):285-96.
12. Carrera AM, Sternke EA, Rivera-Viñas JI. A pilot study of the perceptions of actively practicing obstetricians in Puerto Rico: factors that influence decision making in cesarean delivery. Puerto Rico Health Sciences Journal. 2017;36(1).
13. Chaillet N, Dube E, Dugas M, Francoeur D, Dube J, Gagnon S, et al. Identifying barriers and facilitators towards implementing guidelines to reduce caesarean section rates in Quebec. Bulletin of the World Health Organization. 2007;85(10):791-7.
14. Chen BK, Yang C-Y. Increased Perception of Malpractice Liability and the Practice of Defensive Medicine. Journal of Empirical Legal Studies. 2014;11(3):446-76.
15. Cheng YW, Snowden JM, Handler SJ, Tager IB, Hubbard AE, Caughey AB. Litigation in obstetrics: does defensive medicine contribute to increases in cesarean delivery? The journal of maternal-fetal & neonatal medicine : the official journal of the European Association of Perinatal Medicine, the Federation of Asia and Oceania Perinatal Societies, the International Society of Perinatal Obstet. 2014;27(16):1668-75.

For peer review only

16. Colomar M, Cafferata ML, Aleman A, Castellano G, Elorrio EG, Althabe F, et al. Mode of childbirth in low-risk pregnancies: Nicaraguan physicians' viewpoints. Maternal and child health journal. 2014;18(10):2382-92.
17. Cox KJ. Providers' perspectives on the vaginal birth after cesarean guidelines in Florida, United States: a qualitative study. BMC Pregnancy Childbirth. 2011;11:72.
18. Deutsch AB, Duncan K, Rajaram L, Salihu HM, Spellacy WN, Belogolovkin V. Cesarean or vaginal delivery for the breech fetus at the threshold of viability: results from a maternal-fetal medicine specialists survey. The journal of maternal-fetal & neonatal medicine : the official journal of the European Association of Perinatal Medicine, the Federation of Asia and Oceania Perinatal Societies, the International Society of Perinatal Obstet. 2011;24(3):475-9.
19. Doraiswamy S, Billah SM, Karim F, Siraj MS, Buckingham A, Kingdon C. Physician–patient communication in decision-making about Caesarean sections in eight district hospitals in Bangladesh: a mixed-method study. Reproductive health. 2021;18(1):1-14.
20. Dranove D, Watanabe Y. Influence and Deterrence: How Obstetricians Respond to Litigation against Themselves and Their Colleagues. American Law and Economics Review. 2010;12(1):69-94.
21. Durrance CP, Hankins S. Medical Malpractice Liability Exposure and OB/GYN Physician Delivery Decisions. Health services research. 2018;53(4):2633-50.
22. Esposto AG. Tort reform and caesarean deliveries. Applied Economics Letters. 2012;19(12):1171-4.
23. Firoozi M, Tara F, Ahanchian MR, Latifnejad Roudsari R. Health Care System Barriers to Vaginal Birth after Cesarean Section: A Qualitative Study. Iranian journal of nursing and midwifery research. 2020;25(3):202-11.
24. Frakes M. Defensive medicine and obstetric practices. Journal of Empirical Legal Studies. 2012;9(3):457-81.
25. Fuglenes D, Oian P, Kristiansen IS. Obstetricians' choice of cesarean delivery in ambiguous cases: is it influenced by risk attitude or fear of complaints and litigation? Am J Obstet Gynecol. 2009;200(1):48 e1-8.
26. Gimm GW. The impact of malpractice liability claims on obstetrical practice patterns. Health services research. 2010;45(1):195-211.
27. Goldenberg T, Foster J, Andes KL. Driving birth: Cesarean sections and the medicalization of birth in Gran Asunción, Paraguay. Revista Salud Pública del Paraguay. 2016;6(1):8-15.
28. Grant D, McInnes MM. Malpractice experience and the incidence of cesarean delivery: a physician-level longitudinal analysis. Inquiry : a journal of medical care organization, provision and financing. 2004;41(2):170-88.
29. Habiba M, Kaminski M, Da Fre M, Marsal K, Bleker O, Librero J, et al. Caesarean section on request: a comparison of obstetricians' attitudes in eight European countries. BJOG : an international journal of obstetrics and gynaecology. 2006;113(6):647-56.
30. Hellerstein S, Feldman S, Duan T. Survey of Obstetric Care and Cesarean Delivery Rates in Shanghai, China. Birth (Berkeley, Calif). 2016;43(3):193-9.
31. Indraccolo U, Scutiero G, Matteo M, Indraccolo SR, Greco P. Cesarean section on maternal request: should it be formally prohibited in Italy? Annali dell'Istituto superiore di sanita. 2015;51(2):162-6.
32. Ionescu CA, Dimitriu M, Poenaru E, Banacu M, Furau GO, Navolan D, et al. Defensive caesarean section: A reality and a recommended health care improvement for Romanian obstetrics. Journal of evaluation in clinical practice. 2019;25(1):111-6.

For peer review only - http://bmjopen.bmj.com/site/about/guidelines.xhtml

33. Kamal P, Dixon-Woods M, Kurinczuk JJ, Oppenheimer C, Squire P, Waugh J. Factors influencing repeat caesarean section: qualitative exploratory study of obstetricians' and midwives' accounts. BJOG : an international journal of obstetrics and gynaecology. 2005;112(8):1054-60.
34. Karlstrom A, Engstrom-Olofsson R, Nystedt A, Thomas J, Hildingsson I. Swedish caregivers' attitudes towards caesarean section on maternal request. Women Birth. 2009;22(2):57-63.
35. Koigi-Kamau R, Leting PK, Kiarie JN. Perceptions and practices of vaginal birth after Caesarean section among privately practicing obstetricians in Kenya. East African medical journal. 2005;82(12):631-6.
36. Kuan CI. "Suffering twice": the gender politics of cesarean sections in Taiwan. Med Anthropol Q. 2014;28(3):399-418.
37. Kucuk M. Obstetrician perceptions of the causes of high cesarean delivery rates in Turkey. International journal of gynaecology and obstetrics: the official organ of the International Federation of Gynaecology and Obstetrics. 2017;138(1):100-6.
38. Kucuk M. Defensive medicine among obstetricians and gynaecologists in Turkey. Journal of obstetrics and gynaecology : the journal of the Institute of Obstetrics and Gynaecology. 2018;38(2):200-5.
39. Kwee A, Cohlen BJ, Kanhai HH, Bruinse HW, Visser GH. Caesarean section on request: a survey in The Netherlands. European journal of obstetrics, gynecology, and reproductive biology. 2004;113(2):186-90.
40. Lundgren I, Healy P, Carroll M, Begley C, Matterne A, Gross MM, et al. Clinicians' views of factors of importance for improving the rate of VBAC (vaginal birth after caesarean section): a study from countries with low VBAC rates. BMC Pregnancy Childbirth. 2016;16(1):350.
41. Makhija B, Verma D, Mustafa A. A root cause analysis of increasing caesarean section rates in a tertiary care private hospital in North India. International Journal of Reproduction, Contraception, Obstetrics and Gynecology. 2019;8(11):4531.
42. Munro S, Kornelsen J, Corbett K, Wilcox E, Bansback N, Janssen P. Do Women Have a Choice? Care Providers' and Decision Makers' Perspectives on Barriers to Access of Health Services for Birth after a Previous Cesarean. Birth (Berkeley, Calif). 2017;44(2):153-60.
43. Mushinski D, Zahran S, Frazier A. Physician behaviour, malpractice risk and defensive medicine: an investigation of cesarean deliveries. Health economics, policy, and law. 2021:1-19.
44. Panda S, Daly D, Begley C, Karlström A, Larsson B, Bäck L, et al. Factors influencing decision-making for caesarean section in Sweden – a qualitative study. BMC Pregnancy and Childbirth. 2018;18(1):377.
45. Peel A, Bhartia A, Spicer N, Gautham M. 'If I do 10–15 normal deliveries in a month I hardly ever sleep at home.' A qualitative study of health providers' reasons for high rates of caesarean deliveries in private sector maternity care in Delhi, India. BMC Pregnancy and Childbirth. 2018;18(1):470.
46. Perrotta C, Romero M, Sguassero Y, Straw C, Gialdini C, Righetti N, et al. Caesarean birth in public maternities in Argentina: a formative research study on the views of obstetricians, midwives and trainees. BMJ open. 2022;12(1):e053419.
47. Rudey EL, Leal MDC, Rego G. Defensive medicine and cesarean sections in Brazil. Medicine. 2021;100(1):e24176.

For peer review only - http://bmjopen.bmj.com/site/about/guidelines.xhtml

48. Samadi S, Gholizadeh N, Shoar N, Shoar S. Attitudes of Obstetricians toward Cesarean Delivery in Challenging Cases. J Obstet Gynaecol India. 2013;63(5):301-5.
49. Savage W, Francome C. British consultants' attitudes to caesareans. Journal of obstetrics and gynaecology : the journal of the Institute of Obstetrics and Gynaecology. 2007;27(4):354-9.
50. Wells CE. Vaginal birth after cesarean delivery: views from the private practitioner. Semin Perinatol. 2010;34(5):345-50.
51. Yang YT, Mello MM, Subramanian SV, Studdert DM. Relationship between malpractice litigation pressure and rates of cesarean section and vaginal birth after cesarean section. Med Care. 2009;47(2):234-42.
52. Yazdizadeh B, Nedjat S, Mohammad K, Rashidian A, Changizi N, Majdzadeh R. Cesarean section rate in Iran, multidimensional approaches for behavioral change of providers: a qualitative study. BMC Health Serv Res. 2011;11(1):159.
53. Zwecker P, Azoulay L, Abenhaim HA. Effect of fear of litigation on obstetric care: a nationwide analysis on obstetric practice. American journal of perinatology. 2011;28(4):277-84.

For peer review only

For peer review only - http://bmjopen.bmj.com/site/about/guidelines.xhtml

Preferred Reporting Items for Systematic reviews and Meta-Analyses extension for Scoping Reviews (PRISMA-ScR) Checklist

SECTION	ITEM	PRISMA-ScR CHECKLIST ITEM	REPORTED ON PAGE #
**TITLE**			
Title	1	Identify the report as a scoping review.	1
**ABSTRACT**			
Structured summary	2	Provide a structured summary that includes (as applicable): background, objectives, eligibility criteria, sources of evidence, charting methods, results, and conclusions that relate to the review questions and objectives.	2
**INTRODUCTION**			
Rationale	3	Describe the rationale for the review in the context of what is already known. Explain why the review questions/objectives lend themselves to a scoping review approach.	4-5
Objectives	4	Provide an explicit statement of the questions and objectives being addressed with reference to their key elements (e.g., population or participants, concepts, and context) or other relevant key elements used to conceptualize the review questions and/or objectives.	5
**METHODS**			
Protocol and registration	5	Indicate whether a review protocol exists; state if and where it can be accessed (e.g., a Web address); and if available, provide registration information, including the registration number.	NA
Eligibility criteria	6	Specify characteristics of the sources of evidence used as eligibility criteria (e.g., years considered, language, and publication status), and provide a rationale.	5-6; Supplementary file
Information sources*	7	Describe all information sources in the search (e.g., databases with dates of coverage and contact with authors to identify additional sources), as well as the date the most recent search was executed.	5-6
Search	8	Present the full electronic search strategy for at least 1 database, including any limits used, such that it could be repeated.	Supplementary file
Selection of sources of evidence†	9	State the process for selecting sources of evidence (i.e., screening and eligibility) included in the scoping review.	5-6
Data charting process‡	10	Describe the methods of charting data from the included sources of evidence (e.g., calibrated forms or forms that have been tested by the team before their use, and whether data charting was done independently or in duplicate) and any processes for obtaining and confirming data from investigators.	6; Fig 1
Data items	11	List and define all variables for which data were sought and any assumptions and simplifications made.	Supplementary file
Critical appraisal of individual	12	If done, provide a rationale for conducting a critical appraisal of included sources of evidence; describe	NA

For peer review only

SECTION	ITEM	PRISMA-ScR CHECKLIST ITEM	REPORTED ON PAGE #
sources of evidence§		the methods used and how this information was used in any data synthesis (if appropriate).	
Synthesis of results	13	Describe the methods of handling and summarizing the data that were charted.	7
**RESULTS**			
Selection of sources of evidence	14	Give numbers of sources of evidence screened, assessed for eligibility, and included in the review, with reasons for exclusions at each stage, ideally using a flow diagram.	Fig 1
Characteristics of sources of evidence	15	For each source of evidence, present characteristics for which data were charted and provide the citations.	7; Table 1
Critical appraisal within sources of evidence	16	If done, present data on critical appraisal of included sources of evidence (see item 12).	NA
Results of individual sources of evidence	17	For each included source of evidence, present the relevant data that were charted that relate to the review questions and objectives.	8-15; Table 2
Synthesis of results	18	Summarize and/or present the charting results as they relate to the review questions and objectives.	8-15
**DISCUSSION**			
Summary of evidence	19	Summarize the main results (including an overview of concepts, themes, and types of evidence available), link to the review questions and objectives, and consider the relevance to key groups.	16
Limitations	20	Discuss the limitations of the scoping review process.	19
Conclusions	21	Provide a general interpretation of the results with respect to the review questions and objectives, as well as potential implications and/or next steps.	20
**FUNDING**			
Funding	22	Describe sources of funding for the included sources of evidence, as well as sources of funding for the scoping review. Describe the role of the funders of the scoping review.	21

JBI = Joanna Briggs Institute; PRISMA-ScR = Preferred Reporting Items for Systematic reviews and Meta-Analyses extension for Scoping Reviews.
* Where *sources of evidence* (see second footnote) are compiled from, such as bibliographic databases, social media platforms, and Web sites.
† A more inclusive/heterogeneous term used to account for the different types of evidence or data sources (e.g., quantitative and/or qualitative research, expert opinion, and policy documents) that may be eligible in a scoping review as opposed to only studies. This is not to be confused with *information sources* (see first footnote).
‡ The frameworks by Arksey and O'Malley (6) and Levac and colleagues (7) and the JBI guidance (4, 5) refer to the process of data extraction in a scoping review as data charting.
§ The process of systematically examining research evidence to assess its validity, results, and relevance before using it to inform a decision. This term is used for items 12 and 19 instead of "risk of bias" (which is more applicable to systematic reviews of interventions) to include and acknowledge the various sources of evidence that may be used in a scoping review (e.g., quantitative and/or qualitative research, expert opinion, and policy document).

From: Tricco AC, Lillie E, Zarin W, O'Brien KK, Colquhoun H, Levac D, et al. PRISMA Extension for Scoping Reviews (PRISMAScR): Checklist and Explanation. Ann Intern Med. 2018;169:467–473. doi: 10.7326/M18-0850.

For peer review only

BMJ Open

Behavioural factors associated with fear of litigation as a driver for the increased use of caesarean sections: A scoping review

Journal:	*BMJ Open*
Manuscript ID	bmjopen-2022-070454.R1
Article Type:	Original research
Date Submitted by the Author:	02-Mar-2023
Complete List of Authors:	Elaraby, Sarah; Alexandria University Faculty of Medicine, Community Medicine Department; World Health Organization, Behavioural Insights Unit<br>Altieri, Elena; World Health Organization, Behavioural Insights Unit<br>Downe, Soo; University of Central Lancashire<br>Erdman, Joanna; Dalhousie University<br>Mannava, Sunny; University of Hyderabad<br>Moncrieff, Gill; University of Central Lancashire<br>BR, Shamanna; University of Hyderabad, School of Medical Sciences<br>Torloni, Maria Regina; Sao Paulo Federal University, Department of Medicine<br>Betran, Ana Pilar; World Health Organization
**Primary Subject Heading**:	Obstetrics and gynaecology
Secondary Subject Heading:	Global health, Public health, Qualitative research, Patient-centred medicine
Keywords:	PUBLIC HEALTH, QUALITATIVE RESEARCH, Maternal medicine < OBSTETRICS

SCHOLARONE™
Manuscripts

I, the Submitting Author has the right to grant and does grant on behalf of all authors of the Work (as defined in the below author licence), an exclusive licence and/or a non-exclusive licence for contributions from authors who are: i) UK Crown employees; ii) where BMJ has agreed a CC-BY licence shall apply, and/or iii) in accordance with the terms applicable for US Federal Government officers or employees acting as part of their official duties; on a worldwide, perpetual, irrevocable, royalty-free basis to BMJ Publishing Group Ltd ("BMJ") its licensees and where the relevant Journal is co-owned by BMJ to the co-owners of the Journal, to publish the Work in this journal and any other BMJ products and to exploit all rights, as set out in our licence.

The Submitting Author accepts and understands that any supply made under these terms is made by BMJ to the Submitting Author unless you are acting as an employee on behalf of your employer or a postgraduate student of an affiliated institution which is paying any applicable article publishing charge ("APC") for Open Access articles. Where the Submitting Author wishes to make the Work available on an Open Access basis (and intends to pay the relevant APC), the terms of reuse of such Open Access shall be governed by a Creative Commons licence – details of these licences and which Creative Commons licence will apply to this Work are set out in our licence referred to above.

Other than as permitted in any relevant BMJ Author's Self Archiving Policies, I confirm this Work has not been accepted for publication elsewhere, is not being considered for publication elsewhere and does not duplicate material already published. I confirm all authors consent to publication of this Work and authorise the granting of this licence.

For peer review only

Behavioural factors associated with fear of litigation as a driver for the increased use of caesarean sections: A scoping review

Authors

Sarah Elaraby[1, 2]

Elena Altieri[2]

Soo Downe[3]

Joanna Erdman[4]

Sunny Mannava[5]

Gill Moncrieff [3]

BR Shamanna[5]

Maria Regina Torloni[6]

Ana Pilar Betrán[7*]

* Corresponding author: Ana Pilar Betrán; betrana@who.int

Afiliations

1. Community Medicine Department, Alexandria Faculty of Medicine, Egypt
2. Behavioural Insights Unit, World Health Organization, Switzerland
3. University of Central Lancashire, United Kingdom
4. Dalhousie University, Canada
5. University of Hyderabad, India
6. Evidence Based Healthcare Post-Graduate Program, São Paulo Federal University Brazil
7. UNDP/UNFPA/UNICEF/World Bank Special Program of Research, Development and Research Training in Human Reproduction (HRP), Department of Sexual and Reproductive Health and Research, World Health Organization, Switzerland

For peer review only

Abstract

Objective: To explore the behavioural drivers of fear of litigation among healthcare providers influencing CS rates.

Design: Scoping review.

Data sources: We searched MEDLINE, Scopus, and WHO Global Index (January 1, 2001-March 9th, 2022).

Data extraction and synthesis: Data were extracted using a form specifically designed for this review and we conducted content analysis using textual coding for relevant themes. We used the WHO principles for the adoption of a behavioural science perspective in public health developed by the WHO Technical Advisory Group for Behavioural Sciences and Insights to organize and analyse the findings. We used a narrative approach to summarize the findings.

Results: We screened 2968 citations, and 56 were included. Reviewed articles did not use a standard measure of influence of fear of litigation on provider's behaviour. None of the studies used a clear theoretical framework to discuss the behavioural drivers of fear of litigation. We identified 12 drivers under the three domains of the WHO principles: 1) Cognitive drivers: availability bias, ambiguity aversion, relative risk bias, commission bias, and loss aversion bias; 2) social and cultural drivers: patient pressure, social norms, and blame culture; and 3) environmental drivers: legal, insurance, medical and professional, and media. Cognitive biases were the most discussed drivers of fear of litigation, followed by legal environment, and patient pressure.

Conclusions: Despite lack of consensus on a definition or measurement, we found that fear of litigation as a driver for rising CS rates results from a complex interaction between cognitive, social, and environmental drivers. Many of our findings were transferable across

For peer review only - http://bmjopen.bmj.com/site/about/guidelines.xhtml

geographical and practice settings. Behavioural interventions that consider these drivers are crucial to address fear of litigation as part of strategies to reduce CS.

Keywords: Caesarean sections, litigation, behavioural drivers, scoping review

Summary: Strengths and limitations

- Fear of litigation (FoL) is a well-documented cause for increased rate of caesarean sections (CS) globally. To our knowledge, this is the first review to systematically explore global accounts for FoL and its behavioural drivers.
- This scoping review employs a behavioural approach and the WHO behavioural principles to identify and classify the key cognitive, social and environmental drivers of FoL among health care providers offering clear pathways for the design of interventions and health policies to address these fears and decrease rates of unnecessary CS.
- We may have overlooked evidence from grey-literature and other informal publications.
- While our interest was the understanding the drivers of medically unnecessary CS, the reviewed literature often did not differentiate between necessary and unnecessary CS. Hence, we have approached our study of drivers of FoL in relation to "increasing use of CS" rather than "increasing rates of unnecessary CS".

For peer review only - http://bmjopen.bmj.com/site/about/guidelines.xhtml

Introduction

Over the past decades, rates of caesarean section (CS) have been rising steadily worldwide, in many cases without medical indication (1). Latest available estimates show that worldwide, the average CS rate increased from 6.7% in 1990 to 21% in 2018 (2). The medically unnecessary use of CS impacts the quality of care and is a burden on often stretched healthcare systems (3), in addition to exposing women and offspring to the short- and long-term risks associated with this surgery (4, 5).

Medical litigation can be defined as a legal dispute that involved carrying out a lawsuit or civil action against healthcare providers or a medical entity (6). We use litigation here as an umbrella term to cover a variety of lawsuits including medical malpractice, negligence, and the associated legal liability, risk and settlements.

Fear of litigation (FoL) has been commonly recognized as an influential factor in medical decision making (7). Obstetrics is one of the leading medical specialities in terms of litigation risk and cost (8-11). FoL and defensive medicine (practice wherein a healthcare professional makes decisions out of fear of litigation and not for the benefit of the patients) are commonly cited reasons for performing CS (12-14), sometimes without medical indication (12, 15, 16). Beyond mode of birth (MoB) decision-making, FoL has profound impact maternity care, with studies across the world documenting high rates of defensive medicine (17-22), a decline in the desire to practice obstetrics or early retirement of practicing obstetricians due to litigation concerns (15, 23).

Despite the frequent emphasis on FoL as a driver of increased CS, there is no agreed definition or a standardized tool to measure it, which reduces the ability to assess its role as a driver of CS rates. For the purpose of this review, we rely on explicit mentions of FoL and its consequences in relation to MoB decision-making.

For peer review only - http://bmjopen.bmj.com/site/about/guidelines.xhtml

Behavioural drivers of fear of litigation

Evidence from behavioural science indicates that our decisions and behaviours are often not deliberate and rational, but rather automatic and profoundly influenced by our environment (12, 24). Understanding human behaviour is essential to improve the design and implementation of interventions aiming to influence decision making of providers. To improve understanding of FoL in relation to MoB, and map existing evidence that could inform interventions to optimize the use of CS, we conducted a scoping review asking the following questions: a) how is FoL defined by providers in relation to MoB? b) What are the various behavioural drivers influencing FoL and affecting decision making for MoB?

We used the principles for the adoption of a behavioural science perspective in public health developed by WHO as a guideline to organize and analyse the findings of the review (25). Based on these behavioural principles, we mapped the different types of influences on behaviour, namely cognitive and psychological factors, social and cultural factors, and environment factors driving FoL among providers. The findings of this review further informed this initial mapping.

Methods

The scoping review followed the standard methodology recommended by the Joanna Briggs Institute and is reported in adherence to Preferred Reporting Items for Systematic Reviews and Meta-Analyses for Scoping reviews (PRISMA-ScR) (26) (27).

Data sources and searches

We searched three electronic databases (MEDLINE via PubMed, Scopus, and WHO Global Index) for studies published from January 1, 2001, to March 9th, 2022. The search strategy used two main concepts (and their synonyms): “caesarean section” and “litigation” (detailed

For peer review only - http://bmjopen.bmj.com/site/about/guidelines.xhtml

search strategy in the Supplementary file 1). We also screened the reference lists of reviews and included articles for potentially relevant studies not captured by the electronic search.

Selection criteria

The review included primary research assessing drivers of FoL experienced by providers in relation to MoB or decision-making for CS. We included studies published after 2000 with qualitative, quantitative, or mixed-method designs. Studies from any country or region were eligible for inclusion. Studies were included if they were published in English, Spanish, Portuguese or French. We excluded case reports or case-series (N<6), reports not describing original research (e.g. commentaries, communications, news articles, reviews, etc.), or if they only covered pure legal analysis (i.e. not reporting original data regarding decision-making). Reviews were excluded but their reference list screened for eligible studies. We included articles covering maternity care by providers in any cadre and involved in any health setting or sector.

Study identification and data extraction

Citations retrieved from electronic databases were uploaded into Covidence (Veritas Health Innovation, Melbourne, Australia) and duplicates were excluded. Titles and abstracts were screened in duplicate, and full texts of potentially relevant studies were obtained and assessed by two reviewers independently (SE and APB). Discrepancies were discussed until consensus was reached.

A data-extraction form was specifically designed for this review to capture key aspects of the studies (setting, design, participants, behavioural drivers, and factors affecting FoL; detailed variables extracted in the Supplementary file). We used content analysis to identify emerging themes in the articles, assisted by Atlas.ti 9®, for further analysis of FoL, its drivers and how they influence decision making regarding MoB (28, 29). We developed a qualitative

For peer review only - http://bmjopen.bmj.com/site/about/guidelines.xhtml

codebook informed by previous relevant reviews (12, 24), and the behavioural principles described before (30, 31). One reviewer (SE) supervised by two reviewers (EA and APB) conducted the data extraction and developed the initial codebook. The codebook was iteratively modified as themes emerged inductively during the process of extraction of the included articles in this scoping review, and through continuous team discussions and reflection. We finally cut out redundant themes or those that were either outside the study scope or were included in only one study.

Data synthesis

Using the qualitative coding process, along with the extraction sheet, it was possible to identify and compare different behavioural drivers of FoL within and across studies (28, 29). This started with applying the codebook and extraction sheet to a few studies, and then going through an iterative process, with continuous revision of codes and overarching themes as more studies were analysed. This process was conducted by one reviewer (SE) and discussions among co-authors informed the classification of drivers according to the WHO principles and the definition of how they interact.

In this manuscript, "litigation" and associated terms are used in a non-specific or technical way reflecting their use in the studies reviewed which is often from the perspective of non-legally trained person, namely providers. It also serves as a construct that covers the risk of legal process, and the experience of litigation itself, rather than specific litigation outcomes.

Patient and public involvement

Patients and/or the public were not involved in the design, or conduct, or reporting, or dissemination plans of this scoping review.

For peer review only - http://bmjopen.bmj.com/site/about/guidelines.xhtml

Results

A total of 2968 citation were identified (2944 from electronic databases and 24 from other sources). We selected 440 studies for full-text evaluation and included 56 in this review (Fig 1).

Main characteristics of included studies

Table 1 shows the main characteristics of the 56 included studies. Thirty-four (60.7%) were from high-income countries (HIC) while 22 (39.3%) were from low- and middle-income countries (LMIC). Most (33, 58.9%) had quantitative designs and were published after 2010. All but three included data from a single country; 47 (83.9%) included the perspective of doctors, 12 (21.4%) of midwives, nurses or doulas, 5 (8.9%) of service users and 1 (1.8%) of lawyers. Study details including behavioural drivers reported for each of the 56 studies are presented in Supplementary file 2.

Table 1. Main characteristics of 56 studies included in the scoping review.

Characteristic	N of studies (n=56)	References
Economic category (World Bank classification)		
Low-, lower-, or middle-income countries	22	(13, 14, 17, 32-50)
High income	34	(16, 18, 19, 21, 22, 51-79)
Study design*		
Quantitative	33	(13, 14, 16-19, 21, 22, 44-48, 50, 60-78)
Qualitative	17	(36-43, 52-59, 79)
Mixed methods	6	(32-35, 51)
Year of publication		
2001-2010	17	(16, 19, 33, 37, 46, 51, 53-55, 61, 65, 67-69, 71-73)
2011 to 2022	39	(13, 14, 17, 18, 21, 22, 32, 34-36, 38-45, 47, 48, 52, 56-60, 62-64, 66, 70, 74-79)
Number of countries		
1	53	(13, 14, 16-19, 22, 32-48, 51-55, 58-68, 70-79)
>1	3	(56, 57, 69)
Perspectives included *		
Doctors	47	(13, 14, 16-19, 21, 32-44, 46-64, 68-72, 75-77, 79)

For peer review only - http://bmjopen.bmj.com/site/about/guidelines.xhtml

Midwives, nurses and doulas	12	(33, 40, 49, 50, 52, 54, 58-60, 63, 70, 79)
Service users (pregnant women and their families)	5	(33, 40, 58, 70, 79)
Administrators and health service decision makers	2	(38, 58)
Lawyers	1	(70)
Other (including records)	9	(22, 35, 45, 58, 65-67, 73, 74, 78)

* Articles can include multiple perspectives

Defining and measuring fear of litigation

There was marked heterogeneity in defining or describing FoL in the context of MoB decision-making. Other terms used to imply FoL included liability concerns (22, 38, 45, 63), medico-legal fears (13, 16, 47, 48), and defensive medicine (14, 17, 18, 21, 45, 52, 62, 74, 80). The only tool we found was a risk attitude and fear index developed by Fuglenes, to measure the impact of perceived risk of complaints and malpractice litigation on providers' choice of MoB (16).

Ten studies explored FoL impact on MoB decision-making beyond providers self-report. The proxy measures used to explore FoL included changes to malpractice insurance premiums (49, 62, 67, 73, 74), previous experience with litigation (19, 22, 65, 66), and changes to liability laws (22, 78).

Drivers of fear of litigation

Using the WHO principles (25), we organized the drivers under three domains: 1) Cognitive or psychological drivers; 2) Socio-cultural drivers; and 3) Environmental drivers (Fig 2). For each of the three domains, Table 2 shows the key drivers for FoL identified in this review, their definitions, and frequency and countries from which the findings drive. The number of citations supporting each driver are visualized in Fig 3 showing that the legal environment

For peer review only - http://bmjopen.bmj.com/site/about/guidelines.xhtml

(n=24), the patient pressure (n=19) and the availability bias (n=18) are the most frequently reported drivers in the literature. Findings for each domain are summarized below.

Table 2: Behavioural drivers of fear of litigation (FoL) under the three domains of the WHO principles, definitions, and frequency and countries from which the findings derive.

Domain/ Construct	Definition	Frequency and citations	Countries
**1. Cognitive or psychological drivers** The identified constructs fall under heuristics, which as decision-making strategies rely on a few relevant predictors, while ignoring others. These shortcuts are helpful in some cases but may lead to cognitive biases i.e. a systematic error in judgement when processing information regarding the world around us that eases the cognitive burden of judgement and decision making (24).			
**1.1 Availability bias (in relation to experience with litigation)**	To make judgments of likelihood or frequency based on ease of recall rather than on actual probabilities and pertains to both direct experience, or vicarious through others(24)	18 (14, 16-19, 22, 43, 47, 48, 51, 58, 60, 62, 65-68, 72)	Brazil, Iran, Israel, Norway, Sudan, Turkey, UK, and US
**1.2 Ambiguity aversion: CS "just in case", avoid "worst case scenario"**	The tendency to prefer known or certain probabilities over uncertain probabilities regardless of the actual benefits (24).	16 (13, 14, 34, 36-38, 40, 41, 44, 46, 53-55, 58, 63, 79)	Brazil, Canada, China, Iran, Kenya, Nicaragua, Paraguay, Sweden, Taiwan, Turkey, UK, US,
**1.3 Relative risk bias and beliefs around safety**	Stronger inclination to [a birth mode] when presented with the relative risk than when presented with the same [information] described in terms of the absolute risk (24).	7 (21, 32, 37, 44, 49, 52, 76)	Argentina, Bangladesh, Brazil, Italy, Romania, Spain, and Turkey
**1.4 Commission bias**	Tendency towards action versus inaction.<br>Omission bias: where harmful commissions are usually judged harsher than the corresponding omissions (24).	6 (14, 36, 41, 42, 47, 52)	Brazil, India, Iran, Paraguay, Spain, and Turkey
**1.5 Loss/gain framing or loss aversion bias**	Losses are often perceived as looming larger than corresponding gains (24).	4 (14, 38, 41, 52)	Brazil, Nicaragua, Paraguay, Spain
**2.Social and cultural drivers**			
**2.1 Patient pressure**	Pregnant women, or their families exerting pressure on providers to perform Caesarean delivery on Maternal Request (CDMR), for nonmedical reasons.	19 (32, 33, 35, 38, 39, 41, 43, 50, 51, 53, 55, 56, 61, 69-71, 76, 79, 81).	Bangladesh, Brazil, Canada, France, Germany, India, Iran, Italy, Luxembourg, Netherlands, Nicaragua, Paraguay, Romania, Spain, Sweden, Taiwan, UK, US,
**2.2 Social norms: CS as the new 'normal birth'**	Implicit or explicit rules used by a group and which determine values, beliefs, attitudes and behaviours (82). These norms can be	8 (14, 33, 34, 37, 38, 43, 52, 56)	Brazil, China, Germany, Iran, Ireland, Italy, Nicaragua, Spain

For peer review only - http://bmjopen.bmj.com/site/about/guidelines.xhtml

	descriptive, i.e. what people observe as the typical overt behaviour of others, or injunctive norms, i.e. inference of what is expected of an individual by others (83).		
**2.3 Blame culture**	A culture that does not tolerate error can be a driver to medical excesses, and negative impacts on healthcare quality and patient safety (84, 85).	10 (17, 36, 38, 40, 54-57, 63, 69)	Iran, Ireland, Italy, Netherland, Nicaragua. Sudan, UK, US<br><br>Sweden (mention of absence of blame culture)
**3. Environmental drivers:** Any factor external to the individual that could impact the behaviour. This could include physical environment, or social environment (e.g. medical administrators, peers, patients and their families, etc)			
**3.1 Legal environment**	The legal framework within which providers practice and are faced with liability. This could include tort laws, court systems, admissible evidence, and expert witnesses. This also covers legal mediation, negotiation and settlements outside the court systems	24 (13, 14, 21, 22, 34, 36, 40, 43-45, 47-49, 51, 52, 59, 62-64, 73, 75-78)	Brazil, China, Iran, Italy, Romania, Spain, Sweden, Turkey, UK, US,
**3.2 Malpractice insurance premiums**	The existing insurance mechanisms against malpractice including degree of coverage and premiums	9 (44, 47, 49, 53, 60, 62, 73, 74, 77)	Argentina, Canada, Turkey, US,
**3.3 Medical and professional environment**	The medical system which organizes obstetric practices through medical guidelines, obstetric organizations, or health facility administration	7 (18, 38, 40, 52, 60, 63, 65)	Iran, Israel, Nicaragua, Spain, US,
**3.4 Mass and social media**	This including social and mass media	5 (14, 18, 33, 47, 51)	Brazil, Israel, Turkey, US

1. Cognitive or psychological drivers

1. 1 Availability bias (in relation to experience with litigation)

The impact of malpractice claims was discussed in 18 studies. In these accounts, personal experience of litigation was not essential for inciting FoL, rather 'hearing' about or knowing someone who was sued for malpractice could be enough (18, 48, 65). Malpractice suits can be highly publicized events in the media and within the healthcare system (18, 47) even though their impact is not always tied to adverse judicial outcomes for the provider. The mere

For peer review only - http://bmjopen.bmj.com/site/about/guidelines.xhtml

thought of the experience of being sued due to failure or delay in performing CS, or the hypothesised impact on providers' time and reputation alone influenced decision-making towards a CS (43, 58).

Some authors have quantified the impact of malpractice claims on defensive medicine practices among providers beyond personal experience showing mixed results. Brown et al found that one standard deviation increase in lawsuits was associated with a 1.073 increase in risk of performing CS in United States (19). Also in the United States, Cheng et al reported that clinicians who were sued were more likely to recommend CS than those who had not gone through this experience (17.2 % vs 11.3% respectively, P= 0.008) (62) while Dranove and Watanabe found a modest impact of litigation experience on CS rates of sued obstetricians and their immediate hospital colleagues (65).

1.2 Ambiguity aversion: CS "just in case", avoid "worst case scenario"

Ambiguity, in the form of uncertainty of length and outcome of vaginal birth (VB), was a justification for preferring CS to avoid litigation consequences in 16 studies (13, 14, 34, 36-38, 40, 41, 44, 46, 53-55, 58, 63, 79). This tendency to avoid ambiguity is what leads professionals to perform CS, especially in the context of caesarean delivery on maternal request (CDMR) (55), or vaginal birth after CS (VBAC), or risk of uterine rupture (54, 63).

This ambiguity also may lead providers to shift the responsibility of decision making to the women and their families, rather than giving their professional opinion, as a means of avoiding litigation in case of complications; a CS becomes a sort of insurance policy (55). Some authors note that this behaviour is reinforced by the fact that women and their families are increasingly perceiving adverse events in maternity care as unacceptable (13, 44, 54).

For peer review only - http://bmjopen.bmj.com/site/about/guidelines.xhtml

1.3 Relative risk bias and beliefs around safety

Seven studies explicitly discussed providers' beliefs around the relative safety of CS (32, 37, 44, 49, 50, 52, 76). The inclination to perform CS to avoid litigation risk can be motivated by providers' belief that CS is a safer option than VB, and that CS guarantees the best outcome for mother and child (51). This belief can supersede training, guidelines, or statistical evidence demonstrating the opposite (42).

In LMIC, this belief is further reaffirmed by the reduction in maternal morbidity and mortality due to the introduction –and framing– of CS as a lifesaving intervention in these countries (32). Guidelines and research outlining the benefits of VB could be dismissed by providers in LMIC as perceived not reflecting the realities of their experiences (36).

Even when providers acknowledge the comparative disadvantages of CS to mother and child in the long-term, they may not share this information with their clients (36). As a consequence, the perceived short-term disadvantages of VB are exaggerated or overstated, making VB the less desirable option for providers in light of the risk of litigation, even when VB is objectively safer for women and babies (38).

1.4 Commission bias

Six studies identified drivers that described a tendency to perform CS even if medically unnecessary, just to "do something" rather than wait for uncertain labour events or outcomes (14, 36, 41, 42, 47, 52). With rising rates of CS and lower tolerance for negative maternity care outcomes, providers find waiting for VB challenging, especially if labour is not progressing as expected (36).

Such findings can be categorized as commission bias in maternity care despite evidence that performing CS without medical indication does not reduce risk of lawsuits (86). It contrasts with "omission bias" in other medical specialties (24), where harmful commissions are

For peer review only - http://bmjopen.bmj.com/site/about/guidelines.xhtml

For peer review only

usually judged harsher than the corresponding omissions. In case of complications, maternity care providers perceived that they are more likely to be sued if they failed to perform a CS or delayed it, but unlikely to be sued for performing an unnecessary CS. A study surveying 403 obstetricians in Brazil reported that 67.5% believed VB poses a higher risk of litigation in case of complications, while only 0.5% found that CS had a higher litigation risk (14). A Turkish study found that the perception of VB as a litigation risk was reinforced by wide media coverage of legal cases focusing on delay or failure to perform a CS (47).

1.5 Loss/gain framing or loss aversion bias

Four studies discussed the substantial direct and indirect costs associated with litigation, regardless of the litigation outcome, which underpinned the loss aversion bias pervasiveness in maternity care. Losing a case might result in losing medical licensure, and/or paying large settlements (14, 43, 47). Moreover, regardless of the legal outcome, providers might have to pay higher premiums for malpractice insurance (74), face negative impacts on their reputation (65, 67), and suffer the time and psychological burden of the litigation process itself (43, 62).

2. Socio-cultural drivers

2.1 Patient pressure

Nineteen studies explicitly examined FoL as a driver for providers to agree to a CS based on maternal request and/or pressure by mothers and families (32, 33, 35, 38, 39, 41, 43, 50, 51, 53, 55, 56, 61, 69-71, 76, 79, 81).

Providers' attitudes towards CDMR varied between some providers believing women have a right to choose how they give birth, and others asserting that medical necessity supersedes service users' preferences (71, 76). A study conducted in Italy aimed to explore the perceptions of provider legal liability in case of CDMR among obstetricians, midwives,

For peer review only - http://bmjopen.bmj.com/site/about/guidelines.xhtml

lawyers and patients (70). On average, patients and lawyers rated concession to CDMR as highly appropriate, in contrast to providers who found it inappropriate. Patients and lawyers also rated judicial responsibility of the providers for complications after a CDMR lower than after a VB, suggesting that provider's compliance with maternal choice can be the deciding factor in litigation process. Providers reported that respecting maternal wish for CS is more likely to help avoid litigation in case of complications.

Women's socioeconomic status and education also impacted the providers' perception of their likelihood to be sued in case of complications. Brazilian providers described how more educated women have a higher expectation of care and intolerance to complications during birth, both of which lead to increased risk of litigation if their demands for a CS are not met (33).

2.2 Social Norms: CS as the new 'normal birth'

Eight studies reported providers' concerns that CS is becoming a social norm among women and relatives, since it is widely regarded as safer and 'more modern' than VB (14, 33, 34, 37, 38, 43, 52, 56). In two studies in China and Brazil, providers claimed that CS is expected by service users and families, posing substantial pressure on providers (14, 34). It has been observed that, as countries become more economically stable, families have less children and acceptance or tolerance of adverse events during labour is reduced (18, 34, 43). In these contexts where CS may be seen as the norm, the perception of VB by providers as riskier increases its perceived litigation risk and decreases a provider's ability to defend their performance following a complication (33, 38).

2.3 Blame Culture

Six studies explicitly addressed the effect of blame culture on FoL in relation to CS choice (17, 36, 38, 40, 56, 69), in addition to four studies implicitly discussing effects of blame

For peer review only - http://bmjopen.bmj.com/site/about/guidelines.xhtml

culture (54, 55, 57, 63). This culture can result in hesitancy to even suggest VB as an option for fear of blame in case of complications (55).

In countries where providers expressed less fear of being blamed, they also reported less inclination to perform a CS. In a study on CS decision-making in Sweden, midwives expressed that FoL is not a concern because the medical and legal environment do not foster placing blame, which improves transparency and empowers midwife-led care. Similarly, a study in multiple European countries reported that participants attributed the low rates of VBAC in their countries (Ireland and Italy) to blame culture, in comparison to higher rates in Sweden (56). A qualitative study of three countries with high VBAC rates (Finland, Sweden, and the Netherlands) showed that providers in the Netherlands were concerned with their decline in VBAC rates due to increased risk of litigation and blame in case of complications (57).

3. Environmental drivers

The third principle encompasses the external factors that enable or block a behaviour, including the legal environment, organizational culture, medical bodies policies, guidance, media, and other larger environmental or structural factors that might influence the perceptions of the providers and their decisions (Table 2).

3.1 The legal environment

The legal environment and how it influences maternity care was the focus of 24 studies (13, 14, 21, 22, 34, 36, 40, 43-45, 47-49, 51, 52, 59, 62-64, 73, 75-78). While laws, legal norms and procedures differ by context, we identified common themes across different studies.

The first theme depicted the perceived randomness of legal processes due to the absence of specialized courts, or of a clear legal framework for medical complaints (14, 43), or in some

For peer review only - http://bmjopen.bmj.com/site/about/guidelines.xhtml

cases even a malpractice law (14, 47, 63). These gaps resulted in a perception of lack of fair and specialized legal processes and reinforced providers' perceptions of their vulnerability in case of adverse event, contributing to FoL.

In addition, reliance on expert witnesses in court to testify regarding fault or negligence is a driver for fear of litigation because of the perceived lack of rigour and criteria for who could be considered an expert to assess medical negligence or malpractice or the degree to which they understand the variability in medical opinions (16). In a study in Iran, providers expressed discontent with the lack of training or knowledge of expert witnesses whom they perceived as chosen based on personal relationships, and as having no interest in examining the causes of adverse events, but who rather relied on emotional criteria (43).

The second theme illustrates the lack of clear demarcation within legal systems between malpractice and unavoidable adverse events (44). Providers in some of the included studies perceived that judges, jurors, and expert witnesses assign fault based on empathy with the families' suffering a loss or disability following birth, rather than looking at whether or not this negative outcome was a result of negligence (63). This perception could be reinforced in contexts where limited social or financial support to families and children with disabilities existed, and hence the legal environment seeks to place blame as a way to help the family with the resulting settlement (12).

Once again, this legal environment results in providers considering CS a safer option. If sued after a VB, providers believe they will need to justify not having conducted a CS and whether a CS could have prevented the negative outcome (36, 70). On the other hand, performing a CS when not medically necessary is not recognized as malpractice (16, 36, 70). In addition, depending on the legal context, providers can be at risk of liability for fault even years after the birth. This adds to providers' long-term perceived risk of not performing a CS (43).

For peer review only - http://bmjopen.bmj.com/site/about/guidelines.xhtml

For peer review only

As more cases are settled in favour of families based on adverse outcomes, and more service users are aware of their right to sue if the birth does not go according to their expectations, the legal and financial incentives for litigation are mounting (62). In five studies providers claimed that the settlements themselves have become an incentive for more litigation, and for providers to avoid VB for FoL (14, 34, 43, 52, 67).

3.2 Malpractice insurance premiums

Malpractice insurance premiums and their relationship to FoL was discussed in nine studies (44, 47, 49, 53, 60, 62, 73, 74, 77). A study in the US, found that the likelihood for having a CS increased, and the likelihood for a VD or an instrumental VB decreased, in states where insurance premiums for malpractice exceeded 100,000 USD compared to states where insurance premiums were below 50,000 USD. Inversely, another study found that higher malpractice insurance premiums were not positively associated with recommending CS, but rather that providers who did not know how much they paid for malpractice insurance premiums were less likely to recommend CS, suggesting a possible lesser preoccupation with malpractice risk (62).

3.3 Medical and professional environment

Seven studies discussed medical and professional environments in which providers reported lack of or little protection afforded to them by medical bodies and authorities (18, 38, 40, 52, 60, 63, 65). Medical providers report feeling discouraged or 'demoralized' for having to face these risks without the backing of their colleagues and scientific authorities for their clinical decisions in societies that are becoming increasingly litigious (52). Three articles also included mention that absent or conflicting guidelines regarding CS decision making contributed to their FoL(38, 43, 60). However, administrators in a study in Nicaragua argued that the guidelines exist but are not followed (38).

For peer review only - http://bmjopen.bmj.com/site/about/guidelines.xhtml

3.4 Mass and social media

Media was mentioned as an influential actor in motivating FoL associated with MoB in five studies (14, 18, 33, 47, 51). The sensationalizing of malpractice claims incited fear and frustration among providers. A study in Turkey found that all included providers attested to practicing defensive medicine and being affected by recent high profile malpractice cases in media which resulted in large settlements (47). This media effect, whether traditional or social media, went beyond fearing the risk of litigation, to having a serious impact on providers' mental health, reputation and their approach to their profession (14).

Discussion

This scoping review gathered the evidence available on drivers of FoL when deciding for MoB from the perspective of behavioural theory. The findings highlight the complexity of FoL as a construct that contributes to rising rates of CS across the world. Our use of behavioural principles to develop a conceptual framework for examining drivers FoL at different levels, aided in understanding how they can interact to influence providers' behaviours and choices (Fig 2). This behavioural lens also lent itself to identifying cognitive influences and biases that, while not always explicitly mentioned in the literature, were interpreted through providers' accounts. Identifying such bias could inform the design of behavioural interventions aimed at influencing the driver of FOL.

This review exposed the underlying factors –and the domains they pertain to– which build FoL motivating providers' decision to perform a CS beyond medical need. While our focus was litigation, it is hard to extricate these fears from perceptions of relative safety of CS, fears of disrepute, and consequences of the process of litigation itself rather than its outcome. This more nuanced understanding of fear as a driver for CS is crucial to address unnecessary surgical births globally.

For peer review only - http://bmjopen.bmj.com/site/about/guidelines.xhtml

For peer review only

The review exposed the need for a consensus around measuring FoL in maternity care, given the diversity of proxies used such as malpractice premiums or experience of litigation. Most studies relied on providers self-reported subjective definition, understanding and perception of FoL, without a standardized scale or comparison of actual litigation risk and outcomes. Relying on self-report poses a challenge to reliably quantify and compare the magnitude or burden of FoL, particularly given the multitude of other factors driving the increasing CS rates and which may be beyond the providers' control (87, 88). Using more complex measures such as a 'fear index' as composite measures of FoL as proposed by Fuglenes et al. (16) could provide some value if used in conjunction with other objective measures of litigation risk such as malpractice claims.

Healthcare providers have to rapidly integrate and interpret a complex range of social, psychological, clinical and emotional data relating to both the person they are caring for and the wider context they are working in, in continuously changing situations where often little time is available to make decisions. In maternity care, this is intensified by the fact that the care provider is undertaking this task for both mother and foetus during both pregnancy, labour, and birth, and balancing the sometimes-differing needs of both. Despite existing guidelines, fast decisions require intuitive thinking processes, that jump to the most likely interpretation of and solution to a problem based on patterns of expert knowledge rather than following sequential rules of rationality (89). This review suggests that, in terms of decision for MoB, emotional responses and primarily fear, can be driven by cognitive biases including rational and irrational beliefs regarding safety and risk. These beliefs can supersede training, guidelines or statistical evidence and are deeply entrenched in socio-cultural structures (12, 24). Crucially, the review confirms the influence of sociocultural and environmental factors which encompasses multiple stakeholders including women, families, health professionals,

For peer review only - http://bmjopen.bmj.com/site/about/guidelines.xhtml

healthcare organizations and legal systems. Hence multi-level approaches beyond knowledge-based intervention or training are required to address FoL (90).

This scoping review unveiled the diversity of drivers behind fear as experienced by providers, the domains to which these drivers belong as, the actual risk of litigation and how it manifests in medical practice. Further contextual studies are warranted for in-depth understanding of local dynamics. However, the range of contexts included in the review suggest that underlying concepts revealed in the data synthesis are potentially transferable between quite different contexts.

Our review also highlights the close relationship and interaction between FoL and shifts in norms, particularly through the role and power of media either mainstream or social. Our findings suggest that trial-by-media may be regarded as having a very real immediate influence while the consequences of any litigation process may take years and are ambiguous. The increasing tendency for sensationalist media coverage, and 'click-bait' reinforces this trend (91). Media may also feed into the public perception that CS is the safest and most modern option, and that women should have a choice of elective CS, while downplaying the need to inform people about the risks associated with this choice (as 'fear mongering'). Media pressures may also act to divorce the sense of entitlement to a right to choose from the personal responsibility to accept the consequences for such choices (92). This means that any adverse outcome, even if resulting from personal choices, is deemed to be the fault of someone else, and thus ripe for a litigation case. Further research into how these fears and factors operate and reinforce each other is needed.

Our focus on behavioural drivers for providers means we did not include perspectives of service users and their families, which may be quite different. Although accounts of providers are necessary to understand drivers of FoL, they do not represent an objective measure of the

For peer review only - http://bmjopen.bmj.com/site/about/guidelines.xhtml

strength or frequency of the phenomena described. For example, while 20 of the included studies discuss CDMR as an important driver of increasing CS rates, global rates of CDMR show great variability across regions (0.2%-42%), with upper middle income countries having 11 times the rate of CDMR compared to high income countries (93).

Strengths and limitations

To our knowledge, this is the first review to systematically explore global accounts of FoL and its behavioural drivers in detail providing a broad overview of the evidence on this multidisciplinary topic and identifying research gaps. The use of the WHO behavioural principles enabled us to classify behavioural drivers within specific domains offering clearer pathways for the design of interventions and policies.

Our review has some limitations. We might have overlooked evidence from grey-literature and other informal publications. However, we found and presented data from a range of high-, middle-, and low-income countries. Our classification of cognitive influences on FoL relied on commonly used behavioural constructs and terminologies in medical decision-making literature (12, 24). These constructs may have not been explicitly used by the authors of the studies included to classify their findings, or by participants to describe their own rationale for MoB decision-making. In order to avoid forcing false categories upon the data, we were very conservative in how we classified findings and explicitly sought data that disconfirmed prior assumptions. This was facilitated by the fact that a few distinct themes resonated in the articles across settings. On the other hand, local socio-cultural and environmental influences were often explicitly mentioned in the reviewed articles, and in some cases, were the focus of the study. Although our synthesis showed consistent themes across settings, any initiatives or strategies to address FoL need to be tailored to the specific local context to address the particularities of legislative and health systems and consider how behaviour change

For peer review only - http://bmjopen.bmj.com/site/about/guidelines.xhtml

interventions can be operationalised locally. Lastly, while we were interested in understanding the drivers of medically unnecessary CS, the reviewed literature often did not differentiate between necessary and unnecessary CS, or document the rates of unnecessary CS. Hence, we have approached our study of drivers of FoL in relation to "increasing use of CS" rather than "increasing rates of unnecessary CS".

Conclusion

FoL as a driver for rising CS rates is the result of a complex interaction between cognitive, social, and environment factors. Factors identified across countries include cognitive bias, social pressure from service users, and prevailing norms such as growing intolerance to complications and uncertainty, legal and medical practice environments, and experience with litigation. FoL may be generated less by the actual risk of litigation and more by how the providers perceive this risk and its potential consequences of the process itself independently of the actual legal outcome. Behavioural interventions addressing these drivers are likely to be crucial to address FoL as part of strategies aimed at reducing CS rates.

Acknowledgements

With thanks to Jane Gibbon, Sarah Cordey and Louise Hunt.

Contributorship statement

All authors included on a paper fulfil the criteria of authorship. SE, EA, SD and APB conceived and plan the review. SE and APB screened the studies. SE conducted data extraction and analyses, SE wrote the first draft of the manuscript with support from EA and APB. SD, JE, SM, BRS, MRT made substantial contributions to interpretation, and critically reviewed the manuscript for important intellectual content.

For peer review only - http://bmjopen.bmj.com/site/about/guidelines.xhtml

Competing interest

The authors have declared that no competing interests exist.

Funding

This scoping review was funded by the Behavioural Insights Unit at World Health Organization, the UNDP-UNFPA-UNICEF-WHO-World Bank Special Programme of Research, Development and Research Training in Human Reproduction (HRP), a cosponsored program executed by the World Health Organization (WHO) in the Department of Sexual and Reproductive Health and Research (SRH) and the Gates Grand Challenges 2020: Improving Access to and Use of Safe and Appropriate Cesarean Section (INV-023309). The review was conceived to inform the project Re-JUDGE (https://gcgh.grandchallenges.org/grant/re-judge-reducing-rates-non-medically-indicated-cesarean-sections-through-open-access-multi).

Data sharing statement

All data relevant to the study is included in the article or is available in the supplementary files.

Ethics approval statement

This is a scoping review. No ethics approval was needed.

Figures

Figure 1: Flowchart of process of study identification and inclusion.

Figure 2: Conceptual framework for the classification of drivers of fear of litigation under the three domains defined in the WHO principles.

For peer review only - http://bmjopen.bmj.com/site/about/guidelines.xhtml

Figure 3: Number of citations supporting each driver among 56 citations included in this scoping review.

For peer review only

For peer review only - http://bmjopen.bmj.com/site/about/guidelines.xhtml

References

1. Betran AP, Temmerman M, Kingdon C, Mohiddin A, Opiyo N, Torloni MR, et al. Interventions to reduce unnecessary caesarean sections in healthy women and babies. Lancet. 2018;392(10155):1358-68.
2. Betran AP, Ye J, Moller A-B, Souza JP, Zhang J. Trends and projections of caesarean section rates: global and regional estimates. BMJ Global Health. 2021;6(6):e005671.
3. Gibbons L, Belizan JM, Lauer JA, Betran AP, Merialdi M, Althabe F. Inequities in the use of cesarean section deliveries in the world. American journal of obstetrics and gynecology. 2012;206(4):331. e1-. e19.
4. Steer PJ, Modi N. Elective caesarean sections--risks to the infant. Lancet. 2009;374(9691):675-6.
5. Victora CG, Barros FC. Beware: unnecessary caesarean sections may be hazardous. Lancet. 2006;367(9525):1796-7.
6. Thirumoorthy T. Understanding Medical Negligence and Litigation–Basic for the Medical Professional. BMA News. 2011:12-3.
7. Grant K, White J, Martin J, Haines T. The costs of risk and fear: a qualitative study of risk conceptualisations in allied health resource allocation decision-making. Health, Risk & Society. 2019;21(7-8):373-89.
8. Domingues AP, Moura P, Vieira DN. Obstetric litigation: the importance of the quality of clinical files and its influence on expertise conclusions. Journal of obstetrics and gynaecology : the journal of the Institute of Obstetrics and Gynaecology. 2015;35(2):146-9.
9. Montilla P, Merzagora F, Scolaro E, Requejo J, Ricciardi W, Meli E, et al. Lessons from a multidisciplinary partnership involving women parliamentarians to address the overuse of caesarean section in Italy. BMJ Global Health. 2020;5(2):e002025.
10. Almannie R, Almuhaideb M, Alyami F, Alkhayyal A, Binsaleh S. The status of medical malpractice litigations in Saudi Arabia: Analysis of the annual report. Saudi journal of anaesthesia. 2021;15(2):97-100.
11. Lane J, Bhome R, Somani B. National trends and cost of litigation in UK National Health Service (NHS): a specialty-specific analysis from the past decade. Scottish Medical Journal. 2021;66(4):168-74.
12. Minkoff H. Fear of litigation and cesarean section rates. Semin Perinatol. 2012;36(5):390-4.
13. Kucuk M. Obstetrician perceptions of the causes of high cesarean delivery rates in Turkey. International journal of gynaecology and obstetrics: the official organ of the International Federation of Gynaecology and Obstetrics. 2017;138(1):100-6.
14. Rudey EL, Leal MDC, Rego G. Defensive medicine and cesarean sections in Brazil. Medicine. 2021;100(1):e24176.
15. Adinma J. Litigations and the Obstetrician in Clinical Practice. Ann Med Health Sci Res. 2016;6(2):74-9.
16. Fuglenes D, Oian P, Kristiansen IS. Obstetricians' choice of cesarean delivery in ambiguous cases: is it influenced by risk attitude or fear of complaints and litigation? Am J Obstet Gynecol. 2009;200(1):48 e1-8.
17. Ali AA, Hummeida ME, Elhassan YA, Nabag WO, Ahmed MA, Adam GK. Concept of defensive medicine and litigation among Sudanese doctors working in obstetrics and gynecology. BMC Med Ethics. 2016;17:12.

For peer review only - http://bmjopen.bmj.com/site/about/guidelines.xhtml

18. Asher E, Dvir S, Seidman DS, Greenberg-Dotan S, Kedem A, Sheizaf B, et al. Defensive medicine among obstetricians and gynecologists in tertiary hospitals. PloS one. 2013;8(3):e57108.
19. Brown HS, 3rd. Lawsuit activity, defensive medicine, and small area variation: the case of Cesarean sections revisited. Health economics, policy, and law. 2007;2(Pt 3):285-96.
20. Clark K. Litigation: a threat to obstetric practice? Journal of law and medicine. 2002;9(3):303-22.
21. Fineschi V, Arcangeli M, Di Fazio N, Del Fante Z, Fineschi B, Santoro P, et al. Defensive Medicine in the Management of Cesarean Delivery: A Survey among Italian Physicians. Healthcare (Basel, Switzerland). 2021;9(9).
22. Frakes M. Defensive medicine and obstetric practices. Journal of Empirical Legal Studies. 2012;9(3):457-81.
23. AlDakhil LO. Obstetric and gynecologic malpractice claims in Saudi Arabia: Incidence and cause. Journal of forensic and legal medicine. 2016;40:8-11.
24. Blumenthal-Barby JS, Krieger H. Cognitive biases and heuristics in medical decision making: a critical review using a systematic search strategy. Medical Decision Making. 2015;35(4):539-57.
25. Technical Note from the WHO Technical Advisory Group (TAG) on behavioural insights and science for health. Principles and steps for applying a behavioural perspective to public health: World Health Organization; 2021 [Available from: https://cdn.who.int/media/docs/default-source/documents/bi-tag-technical-note1_principles-and-steps.pdf?sfvrsn=efdefb39_5&download=true.
26. Peters MD, Godfrey CM, Khalil H, McInerney P, Parker D, Soares CB. Guidance for conducting systematic scoping reviews. JBI Evidence Implementation. 2015;13(3):141-6.
27. Tricco AC, Lillie E, Zarin W, O'Brien KK, Colquhoun H, Levac D, et al. PRISMA extension for scoping reviews (PRISMA-ScR): checklist and explanation. Annals of internal medicine. 2018;169(7):467-73.
28. Erlingsson C, Brysiewicz P. A hands-on guide to doing content analysis. Afr J Emerg Med. 2017;7(3):93-9.
29. Graneheim UH, Lindgren BM, Lundman B. Methodological challenges in qualitative content analysis: A discussion paper. Nurse Educ Today. 2017;56:29-34.
30. Bandura A. The evolution of social cognitive theory. Great minds in management. 2005:9-35.
31. Schunk DH, DiBenedetto MK. Motivation and social cognitive theory. Contemporary Educational Psychology. 2020;60:101832.
32. Aminu M, Utz B, Halim A, van den Broek N. Reasons for performing a caesarean section in public hospitals in rural Bangladesh. BMC Pregnancy Childbirth. 2014;14(1):130.
33. Behague DP, Victora CG, Barros FC. Consumer demand for caesarean sections in Brazil: informed decision making, patient choice, or social inequality? A population based birth cohort study linking ethnographic and epidemiological methods. BMJ (Clinical research ed). 2002;324(7343):942-5.
34. Hellerstein S, Feldman S, Duan T. Survey of Obstetric Care and Cesarean Delivery Rates in Shanghai, China. Birth (Berkeley, Calif). 2016;43(3):193-9.
35. Makhija B, Verma D, Mustafa A. A root cause analysis of increasing caesarean section rates in a tertiary care private hospital in North India. International Journal of Reproduction, Contraception, Obstetrics and Gynecology. 2019;8(11):4531.

For peer review only

For peer review only - http://bmjopen.bmj.com/site/about/guidelines.xhtml

36. Bagheri A, Masoudi Alavi N, Abbaszadeh F. Iranian obstetricians' views about the factors that influence pregnant women's choice of delivery method: a qualitative study. Women Birth. 2013;26(1):e45-9.
37. Dias MA, Deslandes SF. [Cesarean sections: risk perception and indication by attending obstetricians in a public maternity hospital in Rio de Janeiro]. Cadernos de saude publica. 2004;20(1):109-16.
38. Colomar M, Cafferata ML, Aleman A, Castellano G, Elorrio EG, Althabe F, et al. Mode of childbirth in low-risk pregnancies: Nicaraguan physicians' viewpoints. Maternal and child health journal. 2014;18(10):2382-92.
39. Doraiswamy S, Billah SM, Karim F, Siraj MS, Buckingham A, Kingdon C. Physician–patient communication in decision-making about Caesarean sections in eight district hospitals in Bangladesh: a mixed-method study. Reproductive health. 2021;18(1):1-14.
40. Firoozi M, Tara F, Ahanchian MR, Latifnejad Roudsari R. Health Care System Barriers to Vaginal Birth after Cesarean Section: A Qualitative Study. Iranian journal of nursing and midwifery research. 2020;25(3):202-11.
41. Goldenberg T, Foster J, Andes KL. Driving birth: Cesarean sections and the medicalization of birth in Gran Asunción, Paraguay. Revista Salud Pública del Paraguay. 2016;6(1):8-15.
42. Peel A, Bhartia A, Spicer N, Gautham M. 'If I do 10–15 normal deliveries in a month I hardly ever sleep at home.' A qualitative study of health providers' reasons for high rates of caesarean deliveries in private sector maternity care in Delhi, India. BMC Pregnancy and Childbirth. 2018;18(1):470.
43. Yazdizadeh B, Nedjat S, Mohammad K, Rashidian A, Changizi N, Majdzadeh R. Cesarean section rate in Iran, multidimensional approaches for behavioral change of providers: a qualitative study. BMC Health Serv Res. 2011;11(1):159.
44. Akpinar F, Kilic F, Ozturk N, Coskun B, Aksar M, Erkaya S. The effect of medical and social conditions on the mode of delivery: a prospective questionnaire-based study applied to 404 Turkish obstetricians. Archives of gynecology and obstetrics. 2021;303(5):1167-74.
45. Chen BK, Yang C-Y. Increased Perception of Malpractice Liability and the Practice of Defensive Medicine. Journal of Empirical Legal Studies. 2014;11(3):446-76.
46. Koigi-Kamau R, Leting PK, Kiarie JN. Perceptions and practices of vaginal birth after Caesarean section among privately practicing obstetricians in Kenya. East African medical journal. 2005;82(12):631-6.
47. Kucuk M. Defensive medicine among obstetricians and gynaecologists in Turkey. Journal of obstetrics and gynaecology : the journal of the Institute of Obstetrics and Gynaecology. 2018;38(2):200-5.
48. Samadi S, Gholizadeh N, Shoar N, Shoar S. Attitudes of Obstetricians toward Cesarean Delivery in Challenging Cases. J Obstet Gynaecol India. 2013;63(5):301-5.
49. Perrotta C, Romero M, Sguassero Y, Straw C, Gialdini C, Righetti N, et al. Caesarean birth in public maternities in Argentina: a formative research study on the views of obstetricians, midwives and trainees. BMJ open. 2022;12(1):e053419.
50. Zhao P, Cai Z, Huang A, Liu C, Li H, Yang S, et al. Why is the labor epidural rate low and cesarean delivery rate high? A survey of Chinese perinatal care providers. PloS one. 2021;16(5):e0251345.
51. Savage W, Francome C. British consultants' attitudes to caesareans. Journal of obstetrics and gynaecology : the journal of the Institute of Obstetrics and Gynaecology. 2007;27(4):354-9.

For peer review only - http://bmjopen.bmj.com/site/about/guidelines.xhtml

52. Bermudez-Tamayo C, Fernandez Ruiz E, Pastor Moreno G, Maroto-Navarro G, Garcia-Mochon L, Perez-Ramos FJ, et al. Barriers and enablers in the implementation of a program to reduce cesarean deliveries. Reprod Health. 2017;14(1):106.
53. Chaillet N, Dube E, Dugas M, Francoeur D, Dube J, Gagnon S, et al. Identifying barriers and facilitators towards implementing guidelines to reduce caesarean section rates in Quebec. Bulletin of the World Health Organization. 2007;85(10):791-7.
54. Kamal P, Dixon-Woods M, Kurinczuk JJ, Oppenheimer C, Squire P, Waugh J. Factors influencing repeat caesarean section: qualitative exploratory study of obstetricians' and midwives' accounts. BJOG : an international journal of obstetrics and gynaecology. 2005;112(8):1054-60.
55. Karlstrom A, Engstrom-Olofsson R, Nystedt A, Thomas J, Hildingsson I. Swedish caregivers' attitudes towards caesarean section on maternal request. Women Birth. 2009;22(2):57-63.
56. Lundgren I, Healy P, Carroll M, Begley C, Matterne A, Gross MM, et al. Clinicians' views of factors of importance for improving the rate of VBAC (vaginal birth after caesarean section): a study from countries with low VBAC rates. BMC Pregnancy Childbirth. 2016;16(1):350.
57. Lundgren I, van Limbeek E, Vehvilainen-Julkunen K, Nilsson C. Clinicians' views of factors of importance for improving the rate of VBAC (vaginal birth after caesarean section): a qualitative study from countries with high VBAC rates. BMC Pregnancy Childbirth. 2015;15(1):196.
58. Munro S, Kornelsen J, Corbett K, Wilcox E, Bansback N, Janssen P. Do Women Have a Choice? Care Providers' and Decision Makers' Perspectives on Barriers to Access of Health Services for Birth after a Previous Cesarean. Birth (Berkeley, Calif). 2017;44(2):153-60.
59. Panda S, Daly D, Begley C, Karlström A, Larsson B, Bäck L, et al. Factors influencing decision-making for caesarean section in Sweden – a qualitative study. BMC Pregnancy and Childbirth. 2018;18(1):377.
60. Barger MK, Dunn JT, Bearman S, DeLain M, Gates E. A survey of access to trial of labor in California hospitals in 2012. BMC Pregnancy Childbirth. 2013;13:83.
61. Bettes BA, Coleman VH, Zinberg S, Spong CY, Portnoy B, DeVoto E, et al. Cesarean delivery on maternal request: obstetrician-gynecologists' knowledge, perception, and practice patterns. Obstet Gynecol. 2007;109(1):57-66.
62. Cheng YW, Snowden JM, Handler SJ, Tager IB, Hubbard AE, Caughey AB. Litigation in obstetrics: does defensive medicine contribute to increases in cesarean delivery? The journal of maternal-fetal & neonatal medicine: the official journal of the European Association of Perinatal Medicine, the Federation of Asia and Oceania Perinatal Societies, the International Society of Perinatal Obstet. 2014;27(16):1668-75.
63. Cox KJ. Providers' perspectives on the vaginal birth after cesarean guidelines in Florida, United States: a qualitative study. BMC Pregnancy Childbirth. 2011;11:72.
64. Deutsch AB, Duncan K, Rajaram L, Salihu HM, Spellacy WN, Belogolovkin V. Cesarean or vaginal delivery for the breech fetus at the threshold of viability: results from a maternal-fetal medicine specialists survey. The journal of maternal-fetal & neonatal medicine: the official journal of the European Association of Perinatal Medicine, the Federation of Asia and Oceania Perinatal Societies, the International Society of Perinatal Obstet. 2011;24(3):475-9.
65. Dranove D, Watanabe Y. Influence and Deterrence: How Obstetricians Respond to Litigation against Themselves and Their Colleagues. American Law and Economics Review. 2010;12(1):69-94.

For peer review only

66. Durrance CP, Hankins S. Medical Malpractice Liability Exposure and OB/GYN Physician Delivery Decisions. Health services research. 2018;53(4):2633-50.
67. Gimm GW. The impact of malpractice liability claims on obstetrical practice patterns. Health services research. 2010;45(1):195-211.
68. Grant D, McInnes MM. Malpractice experience and the incidence of cesarean delivery: a physician-level longitudinal analysis. Inquiry : a journal of medical care organization, provision and financing. 2004;41(2):170-88.
69. Habiba M, Kaminski M, Da Fre M, Marsal K, Bleker O, Librero J, et al. Caesarean section on request: a comparison of obstetricians' attitudes in eight European countries. BJOG : an international journal of obstetrics and gynaecology. 2006;113(6):647-56.
70. Indraccolo U, Scutiero G, Matteo M, Indraccolo SR, Greco P. Cesarean section on maternal request: should it be formally prohibited in Italy? Annali dell'Istituto superiore di sanita. 2015;51(2):162-6.
71. Kwee A, Cohlen BJ, Kanhai HH, Bruinse HW, Visser GH. Caesarean section on request: a survey in The Netherlands. European journal of obstetrics, gynecology, and reproductive biology. 2004;113(2):186-90.
72. Wells CE. Vaginal birth after cesarean delivery: views from the private practitioner. Semin Perinatol. 2010;34(5):345-50.
73. Yang YT, Mello MM, Subramanian SV, Studdert DM. Relationship between malpractice litigation pressure and rates of cesarean section and vaginal birth after cesarean section. Med Care. 2009;47(2):234-42.
74. Zwecker P, Azoulay L, Abenhaim HA. Effect of fear of litigation on obstetric care: a nationwide analysis on obstetric practice. American journal of perinatology. 2011;28(4):277-84.
75. Mushinski D, Zahran S, Frazier A. Physician behaviour, malpractice risk and defensive medicine: an investigation of cesarean deliveries. Health economics, policy, and law. 2021:1-19.
76. Ionescu CA, Dimitriu M, Poenaru E, Banacu M, Furau GO, Navolan D, et al. Defensive caesarean section: A reality and a recommended health care improvement for Romanian obstetrics. Journal of evaluation in clinical practice. 2019;25(1):111-6.
77. Carrera AM, Sternke EA, Rivera-Viñas JI. A pilot study of the perceptions of actively practicing obstetricians in Puerto Rico: factors that influence decision making in cesarean delivery. Puerto Rico Health Sciences Journal. 2017;36(1).
78. Esposto AG. Tort reform and caesarean deliveries. Applied Economics Letters. 2012;19(12):1171-4.
79. Kuan CI. "Suffering twice": the gender politics of cesarean sections in Taiwan. Med Anthropol Q. 2014;28(3):399-418.
80. Ionescu C. Medical malpractice cases spark debate in Romania. Lancet. 2008;371(9611):462-3.
81. Gau ML, Chang CY, Tian SH, Lin KC. Effects of birth ball exercise on pain and self-efficacy during childbirth: a randomised controlled trial in Taiwan. Midwifery. 2011;27(6):e293-300.
82. Cotterill S, Powell R, Rhodes S, Brown B, Roberts J, Tang MY, et al. The impact of social norms interventions on clinical behaviour change among health workers: protocol for a systematic review and meta-analysis. Systematic Reviews. 2019;8(1):176.
83. Borsari B, Carey KB. Descriptive and injunctive norms in college drinking: a meta-analytic integration. J Stud Alcohol. 2003;64(3):331-41.
84. Khatri N, Brown GD, Hicks LL. From a blame culture to a just culture in health care. Health care management review. 2009;34(4):312-22.

For peer review only - http://bmjopen.bmj.com/site/about/guidelines.xhtml

85. Hoffman JR, Kanzaria HK. Intolerance of error and culture of blame drive medical excess. BMJ (Clinical research ed). 2014;349:g5702.
86. Kessler DP, Summerton N, Graham JR. Effects of the medical liability system in Australia, the UK, and the USA. Lancet. 2006;368(9531):240-6.
87. Long Q, Kingdon C, Yang F, Renecle MD, Jahanfar S, Bohren MA, et al. Prevalence of and reasons for women's, family members', and health professionals' preferences for cesarean section in China: A mixed-methods systematic review. PLoS medicine. 2018;15(10):e1002672.
88. Panda S, Begley C, Daly D. Clinicians' views of factors influencing decision-making for caesarean section: A systematic review and metasynthesis of qualitative, quantitative and mixed methods studies. PLoS One. 2018;13(7):e0200941.
89. Whelehan DF, Conlon KC, Ridgway PF. Medicine and heuristics: cognitive biases and medical decision-making. Irish Journal of Medical Science (1971 -). 2020;189(4):1477-84.
90. Organization WH. WHO recommendations non-clinical interventions to reduce unnecessary caesarean sections: World Health Organization; 2018.
91. O'Keeffe M, Nickel B, Dakin T, Maher CG, Albarqouni L, McCaffery K, et al. Journalists' views on media coverage of medical tests and overdiagnosis: a qualitative study. BMJ open. 2021;11(6):e043991.
92. Moretto G, Walsh E, Haggard P. Experience of agency and sense of responsibility. Consciousness and cognition. 2011;20(4):1847-54.
93. Begum T, Saif-Ur-Rahman KM, Yaqoot F, Stekelenburg J, Anuradha S, Biswas T, et al. Global incidence of caesarean deliveries on maternal request: a systematic review and meta-regression. BJOG : an international journal of obstetrics and gynaecology. 2021;128(5):798-806.

For peer review only

For peer review only - http://bmjopen.bmj.com/site/about/guidelines.xhtml

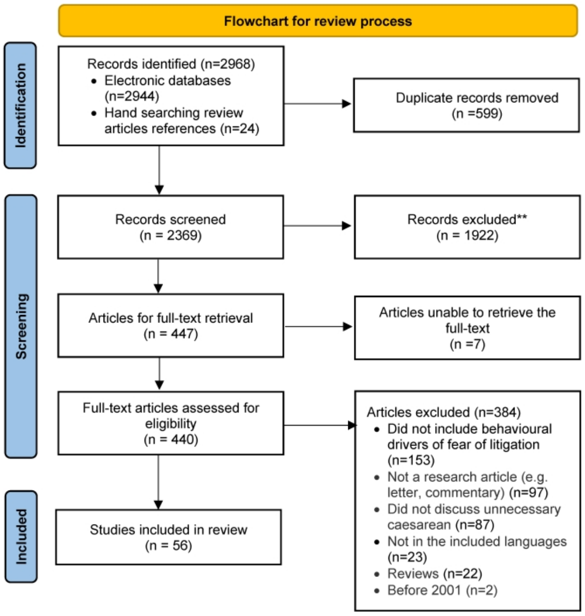

Fig 1: Flowchart of process of study identification and inclusion.

548x710mm (236 x 236 DPI)

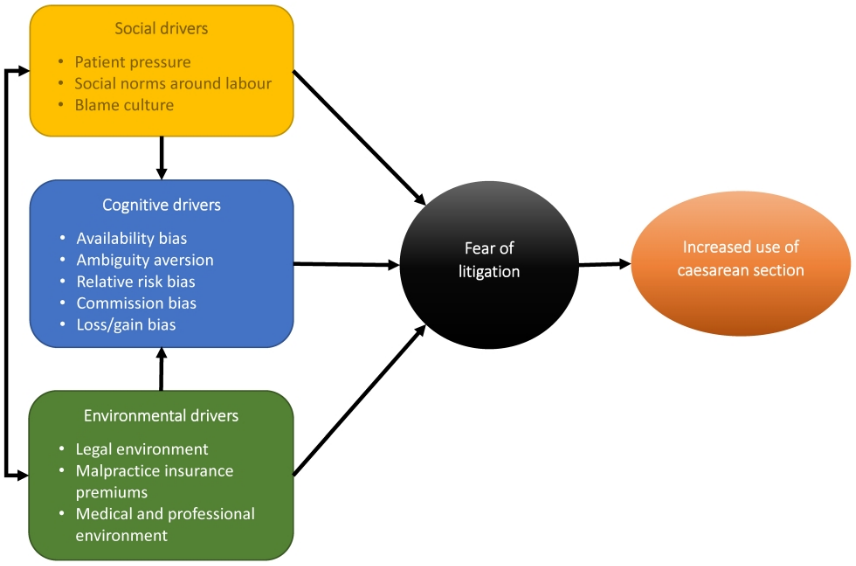

Fig 2: Conceptual framework for the classification of drivers of fear of litigation under the three domains defined in the WHO principles.

710x548mm (236 x 236 DPI)

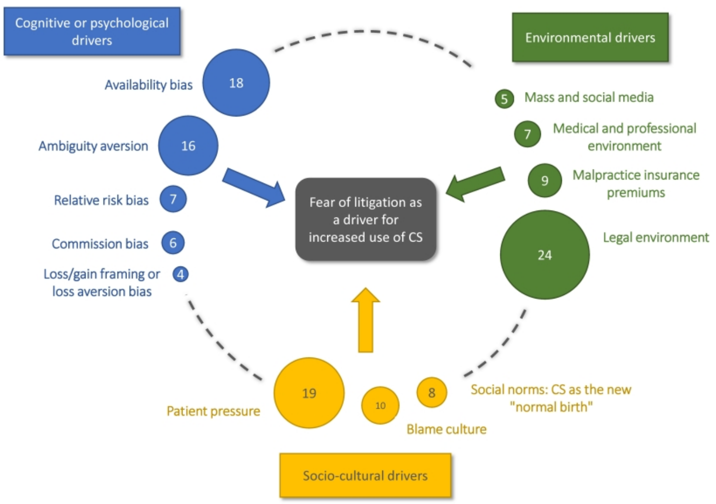

Fig 3: Number of citations supporting each driver among 56 citations included in this scoping review.

861x484mm (236 x 236 DPI)

Supplementary file 1

Search Strategy

PubMed

("OBSTETRICS/LEGISLATION AND JURISPRUDENCE"[MESH] OR "CESAREAN SECTION"[MESH] OR "CESAREAN SECTION"[TIAB] OR "CESAREAN SECTION"[TW] OR "CESAREAN SECTIONS"[TIAB] OR "CESAREAN SECTIONS"[TW] OR "CAESAREAN"[TW] OR "DELIVERY, ABDOMINAL" [TIAB] OR "ABDOMINAL DELIVERIES" [TIAB] OR "DELIVERIES, ABDOMINAL" [TIAB] OR "CAESAREAN SECTION" [TIAB] OR "CAESAREAN SECTIONS" [TIAB] OR "ABDOMINAL DELIVERY" [TIAB] OR "C-SECTION" [TIAB] OR "C SECTION" [TIAB] OR "C-SECTIONS" [TIAB] OR "POSTCESAREAN SECTION"[TIAB])

AND

(LEGIS OR "OBSTETRICS/LEGISLATION AND JURISPRUDENCE"[MESH] OR "JURISPRUDENCE"[MESH] OR "COURT DECISION*" [TIAB] OR "LAW" [TIAB] OR "LAW"[TW] OR "LAWS"[TW] OR "LEGAL*"[TW] OR "LAWY*" [TW] OR "LITIGATION"[TW] OR "LITIGATIONS"[TW] OR "LITIGAT*"[TW] OR "LEGAL CONSEQ*"[TIAB] OR "MEDICAL JURISPRUDENCE" [TW] OR "DEFENSIVE MEDICINE"[MESH] OR "DEFENSIVE PRACTICE*"[TIAB] OR "NEGOTIATING"[MESH] OR "NEGOTIAT*" [TIAB] OR "MEDIATION" [TIAB] OR "MEDIATING" [TIAB] OR "ARBITRATING" [TIAB] OR "ARBITRATION*" [TIAB] OR "CONFLICT RESOLUTION" [TIAB] OR "CONFLICT RESOLUTIONS" [TIAB] OR "RESOLUTION, CONFLICT" [TIAB] OR "MEDICO-LEGAL"[TIAB] OR "VIOLENCE"[TIAB] OR "CASE LAW"[TIAB] OR "LEGAL CASE*"[TIAB] OR "COURT CASE"[TIAB] OR "MALPRACTICE"[MESH] OR "MALPRACTICE"[TIAB] OR "NEGLIGENCE"[TIAB] OR "NEGLIGEN*"[TIAB] OR "MALFEASANCE"[TIAB] OR "MISCONDUCT"[TW] OR "PROFESSIONAL DISCIPLINE"[TW] OR "PATIENT ADVOCACY" [TW] OR "PATIENT RIGHTS"[TW] OR "LIABILITY"[TIAB])

AND

AND PUBYEAR > 2000

Scopus

(TITLE-ABS-KEY(("Caesarean section" OR "Cesarean Section" OR "Caesarean sections" OR "Cesarean Sections" OR "C-section" OR "C-sections" OR "Abdominal delivery" OR "Abdominal deliveries"))

For peer review only - http://bmjopen.bmj.com/site/about/guidelines.xhtml

AND

TITLE-ABS-KEY(("Jurisprudence" OR "Court Decision*" OR "Law" OR "law" OR "laws" OR "legal*" OR "lawy*" OR "litigation" OR "litigations" OR "litigat*" OR "legal conseq*" OR "Medical Jurisprudence" OR "Defensive Medicine" OR "Defensive Practice*" OR "Negotiating" OR "Negotiat*" OR "Mediation" OR "Mediating" OR "Arbitrating" OR "Arbitration" OR "Arbitrations" OR "Conflict Resolution" OR "Conflict Resolutions" OR "medico-legal" OR "Violence" OR "case law" OR "legal case*" OR "court case" OR "Malpractice" OR "malpractice" OR "Negligence" OR "negligen*" OR "malfeasance" OR "misconduct" OR "professional discipline" OR "patient advocacy" OR "patient rights")))

AND PUBYEAR > 2000

WHO Global Index Medicus

("Caesarean section" OR "Cesarean Section" OR "Caesarean sections" OR "Cesarean Sections" OR "C-section" OR "C-sections" OR "Abdominal delivery" OR "Abdominal deliveries")

AND

("Jurisprudence" OR "Court Decision*" OR "Law" OR "law" OR "laws" OR "legal*" OR "lawy*" OR "litigation" OR "litigations" OR "litigat*" OR "legal conseq*" OR "Medical Jurisprudence" OR "Defensive Medicine" OR "Defensive Practice*" OR "Negotiating" OR "Negotiat*" OR "Mediation" OR "Mediating" OR "Arbitrating" OR "Arbitration" OR "Arbitrations" OR "Conflict Resolution" OR "Conflict Resolutions" OR "medico-legal" OR "Violence" OR "case law" OR "legal case*" OR "court case" OR "Malpractice" OR "malpractice" OR "Negligence" OR "negligen*" OR "malfeasance" OR "misconduct" OR "professional discipline" OR "patient advocacy" OR "patient rights")

AND

PUBYEAR > 2000

Detailed inclusion and exclusion criteria

Inclusion criteria

- Focus on caesarean section (not broadly obstetrics).
- Include fear of litigation (broadly defined) in the findings
- Include analysis of empirical data
- Include peer-reviewed articles
- Descriptive or experimental design

For peer review only - http://bmjopen.bmj.com/site/about/guidelines.xhtml

- Can include both quantitative designs/data:
  - *E.g.* cross sectional, cohort, non-randomized controlled trials; program evaluations; interrupted time-series; pre-post designs; pilot studies; etc
  - *N > 6*
- Qualitative designs/data
  - In-depth interviews; focus groups; key-informant interviews
  - *N > 1*
- And mixed methods

- Include drivers of fear of litigation (i.e. goes beyond mere mention of its existence).
  - These can include behavioural determinants or drivers.
  - Can include stakeholders or other actors influencing fear of litigation.
- Publication language: English, Spanish, Portuguese, French, or German
- Published between 2001 and 2021

Exclusion criteria

- Case-studies or case-series
- Studies that report on a study or program where main outcomes are NOT related to unnecessary caesarean sections (broadly discuss obstetric malpractice).
- Reports or articles that do not describe original research (e.g. Abstracts, communications, book chapters, news articles, etc)
- Studies with no mention of fear of litigation or its drivers (beyond its presence based on previous literature)
- Pure legal analysis that does not discuss healthcare decision-making
- Review papers with no substantial analysis of fear of litigation (but screened references for more studies)
- Publication language other than English, Spanish, Portuguese, German or French.
- Published before 2001

Variables collected (data-extraction form)

The following variables were extracted in an excel form specifically designed for this scoping review:

- Author, citation
- Year of the study
- Topic/domain
- Country or countries
- Study design
- Data type
- Sample size
- Type of participant
- Participant characteristics

For peer review only - http://bmjopen.bmj.com/site/about/guidelines.xhtml

- Behavioural drivers
- For each behavioural driver, how the behavioural drivers were assessed
- For each behavioural driver, rational or justification for effect on fear of litigation
- For each behavioural driver, other actors in relation with the behavioural driver
- Key limitations
- Key strengths
- Quotes
- Other notes

For peer review only

For peer review only - http://bmjopen.bmj.com/site/about/guidelines.xhtml

Supplementary file 2: Study details including behavioural drivers reported by each study.

Author, year	Country	Method/data type	Participants and sample size	Behavioural drivers for CS
Akpinar, 2021 (1)	Turkey	Survey<br><br>Quantitative data	404 Obstetricians and Gynaecological administrators and practitioners	Ambiguity aversion<br>Beliefs around safety<br>Legal environment<br>Limited coverage of liability insurance
Ali, 2016 (2)	Sudan	Survey<br><br>Quantitative data	117 OBGYN (42.7% consultants, 34.2% registrars and 23.1% specialists)	Availability bias<br>Blame Culture
Aminu et al., 2014 (3)	Bangladesh	Mixed methods (retrospective review of case notes and Key informant interviews with service providers)	18 OBGYN and case notes of 530 women who had CS in 5 Public Hospitals	Patient Pressure<br>Relative risk bias and Beliefs around safety
Asher, 2013 (4)	Israel	Survey<br><br>Quantitative data	117 board certified physicians and residents in OBGYN departments at tertiary hospitals	Availability bias<br>Medical and professional environment<br>Media
Bagheri, 2013 (5)	Iran	Qualitative data	18 OBGYN	Ambiguity aversion<br>Legal environment
Berger, 2012 (6)	United States	Survey<br><br>Quantitative data	Nurses at 243 civilian California birth hospitals	Availability bias<br>Medical and professional environment<br>Malpractice insurance premiums
Bastos-Dias, 2004 (7)	Brazil	Qualitative data	OBGYNs practicing in one public maternity in Rio de Janeiro, caring for women with low education	Beliefs around safety<br>Ambiguity aversion<br>Social norms
Behague, 2002 (8)	Brazil	Mixed methods	80 mothers from a birth cohort of 5304 women who gave birth in 1993 in any of the hospitals in Pelotas, Brazil. As well as 19 clinicians (six	Patient Pressure<br>Media

For peer review only - http://bmjopen.bmj.com/site/about/guidelines.xhtml

			OBGYN, six paediatricians, four general practitioners, and three nurses)	
Bermúdez-Tamayo, 2017 (9)	Spain	Qualitative data	14 OBGYN and 14 nurse-midwives (NMW) who work for Spain's National Healthcare System	Legal environment<br>Commission bias<br>Loss/gain framing or loss aversion bias<br>Social norms<br>Beliefs around safety
Bettes, 2007 (10)	United States	Survey<br>Quantitative data	699 OBGYN	Patient pressure
Brown, 2007 (11)	United States	Secondary analysis<br>Quantitative data	Hospital discharges data and Insurance Closed Claim File data. Analysis was conducted using a random sample, with replacement of 20,000 births out of the total birth population of 260,292	Availability bias
Carrera, 2017 (12)	United States (Puerto Rico)	Survey<br>Quantitative data	62 OBGYN	Patient pressure<br>Legal System<br>Availability bias<br>Malpractice insurance/premiums
Chaillet, Dube (13)	Canada	Qualitative data	10 Focus group discussions (FGDs) and 6 in-depth interviews (IDIs)	Patient pressure<br>Ambiguity aversion<br>Medical and professional environment
Chen and Yang (14)	Taiwan	Panel analysis<br>Quantitative data	Random subsamples of population of outpatient and inpatient claim in Taiwan (1997-2005) out of 1 million random individual medical claim histories	Legal environment
Cheng, 2014 (15)	United States	Survey<br>Quantitative data	1486 clinicians (general OBGYN, Maternal-Foetal Medicine specialists, family medicine physicians and clinical nurse midwives), who practice obstetrics in the US and who were registered as active members of the American Medical Association (AMA).	Availability bias<br>Malpractice insurance/premiums<br>Legal environment

For peer review only - http://bmjopen.bmj.com/site/about/guidelines.xhtml

Colomar, 2014 (16)	Nicaragua	Qualitative data	4 FGDs, and 17 IDIs with OBGYN and medical decision makers	Patient Pressure<br>Ambiguity aversion<br>Loss/gain framing or loss aversion bias<br>Medical and professional environment
Cox, 2011 (17)	United States	Semi-structured interviews<br><br>Qualitative data	11 obstetricians, 12 midwives, and a hospital administrator (n = 24)	Legal environment<br>Ambiguity aversion<br>Blame Culture
Deutch, 2011 (18)	United States	Survey<br><br>Quantitative data	510 OBGYN (Society for Maternal–Foetal Medicine (SMFM) members)	Legal environment
Doraiswany, 2021 (19)	Bangladesh	IDIs and Labour observations<br><br>Qualitative data	16 physicians and 32 women who delivered by CS	Patient pressure<br>Risk aversion bias (loss gain framing)
Dranove, 2010 (20)	United States	Quantitative record analysis	Using patient-level hospital discharge data and matching physician license of presiding OBGYN to data of closed medical malpractice claims. Examined data of 1539 unique physicians who carried out 98% of the included birth records.	Availability bias<br>Medical and professional environment
Durrance, 2018(21)	United States	Quantitative data	Around 2 million hospital deliveries linked to physician malpractice claim histories (around 2300 doctors) between 1994 and 2010	Availability bias
Esposto, 2012 (22)	United States	Quantitative data	Panel data of CS rates in three years (1987, 1990 and 1993) in 50 states	Legal System (tort reform)
Fineschi, 2021 (23)	Italy	Quantitative data	168 OBGYN	Legal system<br>Patient pressure<br>Relative risk bias and beliefs around safety
Firoozi et al, 2020 (24)	Iran	IDIs and an FGD<br><br>Qualitative data	26 IDIs with maternity care providers (OBGYN and Midwives) and mothers with prior CS	Legal environment<br>Ambiguity aversion<br>Blame culture

For peer review only - http://bmjopen.bmj.com/site/about/guidelines.xhtml

Frakes, 2012 (25)	United States	Secondary quantitative data	Using data on physician behaviour from the 1979–2005 National Hospital Discharge Surveys (NHDS),	Availability bias (Experience with litigation)<br>Legal environment (tort reform)
Fuglenes, 2009 (26)	Norway	Survey<br><br>Quantitative data	716 OBGYN	Perceived risk of litigation (Fear Index)
Gimm, 2010 (27)	United States	Secondary data<br><br>Panel Analysis	Secondary data from the 1992–2000 Florida Hospital Inpatient Discharge File, the Florida Medical Professional Liability Insurance Claims File, and the American Medical Association's Master File on physician characteristics.	Availability bias (Experience with litigation)
Goldenberg, 2016 (28)	Paraguay	IDIs<br><br>Qualitative data	10 OBGYN	Commission bias<br>Loss/gain framing or loss aversion bias<br>Ambiguity aversion<br>Patient pressure
Grant, 2004 (29)	United States	Panel Analysis<br><br>Quantitative data	835 obstetricians who performed at least 10 births in each year and had all the information on physician characteristics	Availability bias
Habiba, 2006 (30)	Luxembourg, Netherlands and Sweden, France, Germany, Italy, Spain and UK	self-administered questionnaire<br><br>Quantitative data	One hundred and five units Neonatal Intensive Care Unit (NICU) associated maternity units, and 1530 obstetricians participated in the study in eight European countries.	Patient pressure
Hellerstein, 2016 (31)	China	Survey and qualitative observations<br><br>Mixed methods data	Descriptive structured survey of obstetric services with standardized interview with obstetricians and qualitative observation of obstetric services in six different Shanghai obstetric facilities	Patient (and family) pressures<br>Ambiguity aversion<br>Social norms regarding birth

For peer review only - http://bmjopen.bmj.com/site/about/guidelines.xhtml

.				
Indraccolo, 2015 (32)	Italy	Survey<br><br>Quantitative data	36 OBGYNs, 42 midwives, 22 lawyers and 25 patients	Patient pressure
Ionsecu, 2018 (33)	Romania	Survey<br><br>Quantitative data	73 OBGYNs	Patient pressure<br>Relative risk bias and beliefs around safety<br>Legal environment
Kamal, 2005 (34)	United Kingdom	IDIs<br><br>Qualitative data	Trainee and consultant obstetricians, and community and hospital-based midwives involved in the care of women in pregnancy and labour in Leicestershire,	Ambiguity aversion<br>Blame Culture
Karlstro¨m, 2009 (35)	Sweden	FGDs<br><br>Qualitative data	5 FGDs where 16 midwives and 9 obstetricians participated.	Ambiguity aversion<br>Patient pressure
Koigi-Kamau, 2005 (36)	Kenya	Survey<br><br>Quantitative data	64 obstetricians with individual private practices (i.e., independent of institutional policies) from the first major towns in Kenya – namely Nairobi, Mombasa, Kisumu, Nakuru and Eldoret.	Ambiguity aversion
Kuan (37)	Taiwan	Multiple Qualitative methods	Interviews with eight obstetricians, two nurses, a doula, a midwife, and 34 pregnant women along with document review, and ethnographic observations	Patient pressure<br>Ambiguity aversion
Kucuk (38)	Turkey	Survey<br><br>Quantitative data	100 obstetricians who responded to the survey.	Legal environment<br>Ambiguity aversion
Kucuk (39)	Turkey	Survey<br><br>Quantitative data	108 practicing OBGYNs	Availability bias<br>Malpractice insurance/premiums<br>Legal environment

For peer review only - http://bmjopen.bmj.com/site/about/guidelines.xhtml

Kwee, Cohlen (40)	Netherlands	Survey<br><br>Quantitative data	583 OBGYN (80% consultants and 20% were senior registrar	Patient pressure
Lundgren, 2016 (41)	Ireland, Italy, and Germany	FGDs<br><br>Qualitative methods	FGDs were held in Ireland, Italy and Germany. In total 71 clinicians participated in nine FGDs.	Social Norms regarding birth<br>Blame culture<br>Patient pressure
Lundgren, 2015 (42)	Finland, Sweden, and the Netherlands	IDIs (face to face and over the telephone)<br><br>FGFs<br><br>Qualitative methods	26 midwives and 18 obstetricians.	Legal System<br>Blame culture (absence of)<br>Media
Makhija et al., 2019 (43)	India	Mixed methods (birth data and IDIs)	20 consultant OBGYNs	Patient pressure
Munro, 2017 (44)	Canada	Qualitative methods	35 Family physicians, midwives, obstetricians, nurses, anaesthetists, and health service decision makers recruited from 3 rural and 2 urban Canadian communities	Availability bias<br>Ambiguity aversion
Mushiniski, 2021 (45)	United States	Quantitative data		Legal environment
Panda, Daly (46)	Sweden	FGDs<br><br>Qualitative methods	4 FGDs with OBGYNs and Midwives involved in birth decision making	Legal environment
Peel, Bhartia (47)	India	IDIs<br><br>Qualitative methods	14 OBGYN and providers involved in maternity care	Relative Risk bias and beliefs around safety<br>Commission bias
Perrotta, 2021 (48)	Argentina	Key informant interviews (KII) and Surveys<br><br>Mixed methods	680 surveys and 26 KIIs of heads of hospital departments, OBGYN, midwives and residents	Legal system<br>Relative Risk bias and beliefs around safety<br>Malpractice insurance/premiums and coverage

For peer review only - http://bmjopen.bmj.com/site/about/guidelines.xhtml

Rudey, 2021 (49)	Brazil	Survey<br><br>Quantitative data	403 General obstetricians who are members of premier Brazilian professional associations of gynaecology and obstetrics	Commission bias<br>Loss/gain framing or loss aversion bias<br>Ambiguity aversion<br>Availability bias<br>Legal environment<br>Media
Samadi, 2013 (50)	Iran	Survey<br><br>Quantitative data	75 OBGYN	Availability bias (Experience with litigation)<br>Legal environment
Savage, 2007 (51)	United Kingdom	Mixed methods	151 OBGYN and clinical directors in OBGYN listed by the royal college of OBGTN in British Isles	Experience with litigation (financial and time drain)<br>Legal environnement<br>Patient pressure<br>Media
Wells, 2010 (52)	United States	Survey<br><br>Quantitative data	458 practicing obstetricians	Availability bias (Experience with litigation)
Yang, 2009 (53)	United States	Quantitative data	52 million recorded births state-level longitudinal mixed-effects regression models to examine data from the Natality Detail File on births in the United States (1991–2003)	Malpractice insurance/premiums<br>Legal environment
Yazdizadeh, 2011 (54)	Iran	IDIs and document analysis<br><br>Qualitative data	26 providers from various public and private hospitals responding to the ministry's C-section reduction interventions. Participants included hospital director, obstetricians and midwives.	Availability bias (Experience with litigation)<br>Legal environment (judges' capability, unrealistic expectation from medical professionals, and large settlements)<br>Patient pressure
Zhao, 2021(55)	China	Survey<br><br>Quantitative data	606/1412 anesthesiologists , 495 obstetricians, 219 midwives and labor & delivery nurses, 1412 hospital administrators and 59 specialties unspecified.	Legal environment<br>Patient pressure<br>Relative risk bias and beliefs around safety

For peer review only - http://bmjopen.bmj.com/site/about/guidelines.xhtml

Zwecker, 2011 (56)	United States	Quantitative record data	890,266 records of women who delivered and malpractice premium data in 37 states in 2006	Malpractice insurance/premiums

For peer review only

For peer review only - http://bmjopen.bmj.com/site/about/guidelines.xhtml

References

1. Akpinar F, Kilic F, Ozturk N, Coskun B, Aksar M, Erkaya S. The effect of medical and social conditions on the mode of delivery: a prospective questionnaire-based study applied to 404 Turkish obstetricians. Archives of gynecology and obstetrics. 2021;303(5):1167-74.
2. Ali AA, Hummeida ME, Elhassan YA, Nabag WO, Ahmed MA, Adam GK. Concept of defensive medicine and litigation among Sudanese doctors working in obstetrics and gynecology. BMC Med Ethics. 2016;17:12.
3. Aminu M, Utz B, Halim A, van den Broek N. Reasons for performing a caesarean section in public hospitals in rural Bangladesh. BMC Pregnancy Childbirth. 2014;14(1):130.
4. Asher E, Dvir S, Seidman DS, Greenberg-Dotan S, Kedem A, Sheizaf B, et al. Defensive medicine among obstetricians and gynecologists in tertiary hospitals. PloS one. 2013;8(3):e57108.
5. Bagheri A, Masoudi Alavi N, Abbaszadeh F. Iranian obstetricians' views about the factors that influence pregnant women's choice of delivery method: a qualitative study. Women Birth. 2013;26(1):e45-9.
6. Barger MK, Dunn JT, Bearman S, DeLain M, Gates E. A survey of access to trial of labor in California hospitals in 2012. BMC Pregnancy Childbirth. 2013;13:83.
7. Dias MA, Deslandes SF. [Cesarean sections: risk perception and indication by attending obstetricians in a public maternity hospital in Rio de Janeiro]. Cadernos de saude publica. 2004;20(1):109-16.
8. Behague DP, Victora CG, Barros FC. Consumer demand for caesarean sections in Brazil: informed decision making, patient choice, or social inequality? A population based birth cohort study linking ethnographic and epidemiological methods. BMJ (Clinical research ed). 2002;324(7343):942-5.
9. Bermudez-Tamayo C, Fernandez Ruiz E, Pastor Moreno G, Maroto-Navarro G, Garcia-Mochon L, Perez-Ramos FJ, et al. Barriers and enablers in the implementation of a program to reduce cesarean deliveries. Reprod Health. 2017;14(1):106.
10. Bettes BA, Coleman VH, Zinberg S, Spong CY, Portnoy B, DeVoto E, et al. Cesarean delivery on maternal request: obstetrician-gynecologists' knowledge, perception, and practice patterns. Obstet Gynecol. 2007;109(1):57-66.
11. Brown HS, 3rd. Lawsuit activity, defensive medicine, and small area variation: the case of Cesarean sections revisited. Health economics, policy, and law. 2007;2(Pt 3):285-96.
12. Carrera AM, Sternke EA, Rivera-Viñas JI. A pilot study of the perceptions of actively practicing obstetricians in Puerto Rico: factors that influence decision making in cesarean delivery. Puerto Rico Health Sciences Journal. 2017;36(1).
13. Chaillet N, Dube E, Dugas M, Francoeur D, Dube J, Gagnon S, et al. Identifying barriers and facilitators towards implementing guidelines to reduce caesarean section rates in Quebec. Bulletin of the World Health Organization. 2007;85(10):791-7.
14. Chen BK, Yang C-Y. Increased Perception of Malpractice Liability and the Practice of Defensive Medicine. Journal of Empirical Legal Studies. 2014;11(3):446-76.
15. Cheng YW, Snowden JM, Handler SJ, Tager IB, Hubbard AE, Caughey AB. Litigation in obstetrics: does defensive medicine contribute to increases in cesarean delivery? The journal of maternal-fetal & neonatal medicine : the official journal of the European Association of Perinatal Medicine, the Federation of Asia and Oceania Perinatal Societies, the International Society of Perinatal Obstet. 2014;27(16):1668-75.
16. Colomar M, Cafferata ML, Aleman A, Castellano G, Elorrio EG, Althabe F, et al. Mode of childbirth in low-risk pregnancies: Nicaraguan physicians' viewpoints. Maternal and child health journal. 2014;18(10):2382-92.
17. Cox KJ. Providers' perspectives on the vaginal birth after cesarean guidelines in Florida, United States: a qualitative study. BMC Pregnancy Childbirth. 2011;11:72.

For peer review only - http://bmjopen.bmj.com/site/about/guidelines.xhtml

18. Deutsch AB, Duncan K, Rajaram L, Salihu HM, Spellacy WN, Belogolovkin V. Cesarean or vaginal delivery for the breech fetus at the threshold of viability: results from a maternal-fetal medicine specialists survey. The journal of maternal-fetal & neonatal medicine : the official journal of the European Association of Perinatal Medicine, the Federation of Asia and Oceania Perinatal Societies, the International Society of Perinatal Obstet. 2011;24(3):475-9.
19. Doraiswamy S, Billah SM, Karim F, Siraj MS, Buckingham A, Kingdon C. Physician–patient communication in decision-making about Caesarean sections in eight district hospitals in Bangladesh: a mixed-method study. Reproductive health. 2021;18(1):1-14.
20. Dranove D, Watanabe Y. Influence and Deterrence: How Obstetricians Respond to Litigation against Themselves and Their Colleagues. American Law and Economics Review. 2010;12(1):69-94.
21. Durrance CP, Hankins S. Medical Malpractice Liability Exposure and OB/GYN Physician Delivery Decisions. Health services research. 2018;53(4):2633-50.
22. Esposto AG. Tort reform and caesarean deliveries. Applied Economics Letters. 2012;19(12):1171-4.
23. Fineschi V, Arcangeli M, Di Fazio N, Del Fante Z, Fineschi B, Santoro P, et al. Defensive Medicine in the Management of Cesarean Delivery: A Survey among Italian Physicians. Healthcare (Basel, Switzerland). 2021;9(9).
24. Firoozi M, Tara F, Ahanchian MR, Latifnejad Roudsari R. Health Care System Barriers to Vaginal Birth after Cesarean Section: A Qualitative Study. Iranian journal of nursing and midwifery research. 2020;25(3):202-11.
25. Frakes M. Defensive medicine and obstetric practices. Journal of Empirical Legal Studies. 2012;9(3):457-81.
26. Fuglenes D, Oian P, Kristiansen IS. Obstetricians' choice of cesarean delivery in ambiguous cases: is it influenced by risk attitude or fear of complaints and litigation? Am J Obstet Gynecol. 2009;200(1):48 e1-8.
27. Gimm GW. The impact of malpractice liability claims on obstetrical practice patterns. Health services research. 2010;45(1):195-211.
28. Goldenberg T, Foster J, Andes KL. Driving birth: Cesarean sections and the medicalization of birth in Gran Asunción, Paraguay. Revista Salud Pública del Paraguay. 2016;6(1):8-15.
29. Grant D, McInnes MM. Malpractice experience and the incidence of cesarean delivery: a physician-level longitudinal analysis. Inquiry : a journal of medical care organization, provision and financing. 2004;41(2):170-88.
30. Habiba M, Kaminski M, Da Fre M, Marsal K, Bleker O, Librero J, et al. Caesarean section on request: a comparison of obstetricians' attitudes in eight European countries. BJOG : an international journal of obstetrics and gynaecology. 2006;113(6):647-56.
31. Hellerstein S, Feldman S, Duan T. Survey of Obstetric Care and Cesarean Delivery Rates in Shanghai, China. Birth (Berkeley, Calif). 2016;43(3):193-9.
32. Indraccolo U, Scutiero G, Matteo M, Indraccolo SR, Greco P. Cesarean section on maternal request: should it be formally prohibited in Italy? Annali dell'Istituto superiore di sanita. 2015;51(2):162-6.
33. Ionescu CA, Dimitriu M, Poenaru E, Banacu M, Furau GO, Navolan D, et al. Defensive caesarean section: A reality and a recommended health care improvement for Romanian obstetrics. Journal of evaluation in clinical practice. 2019;25(1):111-6.
34. Kamal P, Dixon-Woods M, Kurinczuk JJ, Oppenheimer C, Squire P, Waugh J. Factors influencing repeat caesarean section: qualitative exploratory study of obstetricians' and midwives' accounts. BJOG : an international journal of obstetrics and gynaecology. 2005;112(8):1054-60.
35. Karlstrom A, Engstrom-Olofsson R, Nystedt A, Thomas J, Hildingsson I. Swedish caregivers' attitudes towards caesarean section on maternal request. Women Birth. 2009;22(2):57-63.
36. Koigi-Kamau R, Leting PK, Kiarie JN. Perceptions and practices of vaginal birth after Caesarean section among privately practicing obstetricians in Kenya. East African medical journal. 2005;82(12):631-6.

For peer review only - http://bmjopen.bmj.com/site/about/guidelines.xhtml

37. Kuan CI. "Suffering twice": the gender politics of cesarean sections in Taiwan. Med Anthropol Q. 2014;28(3):399-418.
38. Kucuk M. Obstetrician perceptions of the causes of high cesarean delivery rates in Turkey. International journal of gynaecology and obstetrics: the official organ of the International Federation of Gynaecology and Obstetrics. 2017;138(1):100-6.
39. Kucuk M. Defensive medicine among obstetricians and gynaecologists in Turkey. Journal of obstetrics and gynaecology : the journal of the Institute of Obstetrics and Gynaecology. 2018;38(2):200-5.
40. Kwee A, Cohlen BJ, Kanhai HH, Bruinse HW, Visser GH. Caesarean section on request: a survey in The Netherlands. European journal of obstetrics, gynecology, and reproductive biology. 2004;113(2):186-90.
41. Lundgren I, Healy P, Carroll M, Begley C, Matterne A, Gross MM, et al. Clinicians' views of factors of importance for improving the rate of VBAC (vaginal birth after caesarean section): a study from countries with low VBAC rates. BMC Pregnancy Childbirth. 2016;16(1):350.
42. Lundgren I, van Limbeek E, Vehvilainen-Julkunen K, Nilsson C. Clinicians' views of factors of importance for improving the rate of VBAC (vaginal birth after caesarean section): a qualitative study from countries with high VBAC rates. BMC Pregnancy Childbirth. 2015;15(1):196.
43. Makhija B, Verma D, Mustafa A. A root cause analysis of increasing caesarean section rates in a tertiary care private hospital in North India. International Journal of Reproduction, Contraception, Obstetrics and Gynecology. 2019;8(11):4531.
44. Munro S, Kornelsen J, Corbett K, Wilcox E, Bansback N, Janssen P. Do Women Have a Choice? Care Providers' and Decision Makers' Perspectives on Barriers to Access of Health Services for Birth after a Previous Cesarean. Birth (Berkeley, Calif). 2017;44(2):153-60.
45. Mushinski D, Zahran S, Frazier A. Physician behaviour, malpractice risk and defensive medicine: an investigation of cesarean deliveries. Health economics, policy, and law. 2021:1-19.
46. Panda S, Daly D, Begley C, Karlström A, Larsson B, Bäck L, et al. Factors influencing decision-making for caesarean section in Sweden – a qualitative study. BMC Pregnancy and Childbirth. 2018;18(1):377.
47. Peel A, Bhartia A, Spicer N, Gautham M. 'If I do 10–15 normal deliveries in a month I hardly ever sleep at home.' A qualitative study of health providers' reasons for high rates of caesarean deliveries in private sector maternity care in Delhi, India. BMC Pregnancy and Childbirth. 2018;18(1):470.
48. Perrotta C, Romero M, Sguassero Y, Straw C, Gialdini C, Righetti N, et al. Caesarean birth in public maternities in Argentina: a formative research study on the views of obstetricians, midwives and trainees. BMJ open. 2022;12(1):e053419.
49. Rudey EL, Leal MDC, Rego G. Defensive medicine and cesarean sections in Brazil. Medicine. 2021;100(1):e24176.
50. Samadi S, Gholizadeh N, Shoar N, Shoar S. Attitudes of Obstetricians toward Cesarean Delivery in Challenging Cases. J Obstet Gynaecol India. 2013;63(5):301-5.
51. Savage W, Francome C. British consultants' attitudes to caesareans. Journal of obstetrics and gynaecology : the journal of the Institute of Obstetrics and Gynaecology. 2007;27(4):354-9.
52. Wells CE. Vaginal birth after cesarean delivery: views from the private practitioner. Semin Perinatol. 2010;34(5):345-50.
53. Yang YT, Mello MM, Subramanian SV, Studdert DM. Relationship between malpractice litigation pressure and rates of cesarean section and vaginal birth after cesarean section. Med Care. 2009;47(2):234-42.
54. Yazdizadeh B, Nedjat S, Mohammad K, Rashidian A, Changizi N, Majdzadeh R. Cesarean section rate in Iran, multidimensional approaches for behavioral change of providers: a qualitative study. BMC Health Serv Res. 2011;11(1):159.

For peer review only - http://bmjopen.bmj.com/site/about/guidelines.xhtml

55. Zhao P, Cai Z, Huang A, Liu C, Li H, Yang S, et al. Why is the labor epidural rate low and cesarean delivery rate high? A survey of Chinese perinatal care providers. PloS one. 2021;16(5):e0251345.
56. Zwecker P, Azoulay L, Abenhaim HA. Effect of fear of litigation on obstetric care: a nationwide analysis on obstetric practice. American journal of perinatology. 2011;28(4):277-84.

For peer review only

For peer review only - http://bmjopen.bmj.com/site/about/guidelines.xhtml

Preferred Reporting Items for Systematic reviews and Meta-Analyses extension for Scoping Reviews (PRISMA-ScR) Checklist

SECTION	ITEM	PRISMA-ScR CHECKLIST ITEM	REPORTED ON PAGE #
**TITLE**			
Title	1	Identify the report as a scoping review.	1
**ABSTRACT**			
Structured summary	2	Provide a structured summary that includes (as applicable): background, objectives, eligibility criteria, sources of evidence, charting methods, results, and conclusions that relate to the review questions and objectives.	2
**INTRODUCTION**			
Rationale	3	Describe the rationale for the review in the context of what is already known. Explain why the review questions/objectives lend themselves to a scoping review approach.	4-5
Objectives	4	Provide an explicit statement of the questions and objectives being addressed with reference to their key elements (e.g., population or participants, concepts, and context) or other relevant key elements used to conceptualize the review questions and/or objectives.	5
**METHODS**			
Protocol and registration	5	Indicate whether a review protocol exists; state if and where it can be accessed (e.g., a Web address); and if available, provide registration information, including the registration number.	NA
Eligibility criteria	6	Specify characteristics of the sources of evidence used as eligibility criteria (e.g., years considered, language, and publication status), and provide a rationale.	5-6; Supplementary file
Information sources*	7	Describe all information sources in the search (e.g., databases with dates of coverage and contact with authors to identify additional sources), as well as the date the most recent search was executed.	5-6
Search	8	Present the full electronic search strategy for at least 1 database, including any limits used, such that it could be repeated.	Supplementary file
Selection of sources of evidence†	9	State the process for selecting sources of evidence (i.e., screening and eligibility) included in the scoping review.	5-6
Data charting process‡	10	Describe the methods of charting data from the included sources of evidence (e.g., calibrated forms or forms that have been tested by the team before their use, and whether data charting was done independently or in duplicate) and any processes for obtaining and confirming data from investigators.	6; Fig 1
Data items	11	List and define all variables for which data were sought and any assumptions and simplifications made.	Supplementary file
Critical appraisal of individual	12	If done, provide a rationale for conducting a critical appraisal of included sources of evidence; describe	NA

For peer review only - http://bmjopen.bmj.com/site/about/guidelines.xhtml

SECTION	ITEM	PRISMA-ScR CHECKLIST ITEM	REPORTED ON PAGE #
sources of evidence§		the methods used and how this information was used in any data synthesis (if appropriate).	
Synthesis of results	13	Describe the methods of handling and summarizing the data that were charted.	7
**RESULTS**			
Selection of sources of evidence	14	Give numbers of sources of evidence screened, assessed for eligibility, and included in the review, with reasons for exclusions at each stage, ideally using a flow diagram.	Fig 1
Characteristics of sources of evidence	15	For each source of evidence, present characteristics for which data were charted and provide the citations.	7; Table 1
Critical appraisal within sources of evidence	16	If done, present data on critical appraisal of included sources of evidence (see item 12).	NA
Results of individual sources of evidence	17	For each included source of evidence, present the relevant data that were charted that relate to the review questions and objectives.	8-15; Table 2
Synthesis of results	18	Summarize and/or present the charting results as they relate to the review questions and objectives.	8-15
**DISCUSSION**			
Summary of evidence	19	Summarize the main results (including an overview of concepts, themes, and types of evidence available), link to the review questions and objectives, and consider the relevance to key groups.	16
Limitations	20	Discuss the limitations of the scoping review process.	19
Conclusions	21	Provide a general interpretation of the results with respect to the review questions and objectives, as well as potential implications and/or next steps.	20
**FUNDING**			
Funding	22	Describe sources of funding for the included sources of evidence, as well as sources of funding for the scoping review. Describe the role of the funders of the scoping review.	21

JBI = Joanna Briggs Institute; PRISMA-ScR = Preferred Reporting Items for Systematic reviews and Meta-Analyses extension for Scoping Reviews.

* Where *sources of evidence* (see second footnote) are compiled from, such as bibliographic databases, social media platforms, and Web sites.

† A more inclusive/heterogeneous term used to account for the different types of evidence or data sources (e.g., quantitative and/or qualitative research, expert opinion, and policy documents) that may be eligible in a scoping review as opposed to only studies. This is not to be confused with *information sources* (see first footnote).

‡ The frameworks by Arksey and O'Malley (6) and Levac and colleagues (7) and the JBI guidance (4, 5) refer to the process of data extraction in a scoping review as data charting.

§ The process of systematically examining research evidence to assess its validity, results, and relevance before using it to inform a decision. This term is used for items 12 and 19 instead of "risk of bias" (which is more applicable to systematic reviews of interventions) to include and acknowledge the various sources of evidence that may be used in a scoping review (e.g., quantitative and/or qualitative research, expert opinion, and policy document).

From: Tricco AC, Lillie E, Zarin W, O'Brien KK, Colquhoun H, Levac D, et al. PRISMA Extension for Scoping Reviews (PRISMAScR): Checklist and Explanation. Ann Intern Med. 2018;169:467–473. doi: 10.7326/M18-0850.

For peer review only - http://bmjopen.bmj.com/site/about/guidelines.xhtml

BMJ Open

Behavioural factors associated with fear of litigation as a driver for the increased use of caesarean sections: A scoping review

Journal:	*BMJ Open*
Manuscript ID	bmjopen-2022-070454.R2
Article Type:	Original research
Date Submitted by the Author:	03-Apr-2023
Complete List of Authors:	Elaraby, Sarah; Alexandria University Faculty of Medicine, Community Medicine Department; World Health Organization, Behavioural Insights Unit<br>Altieri, Elena; World Health Organization, Behavioural Insights Unit<br>Downe, Soo; University of Central Lancashire<br>Erdman, Joanna; Dalhousie University<br>Mannava, Sunny; University of Hyderabad<br>Moncrieff, Gill; University of Central Lancashire<br>BR, Shamanna; University of Hyderabad, School of Medical Sciences<br>Torloni, Maria Regina; Sao Paulo Federal University, Department of Medicine<br>Betran, Ana Pilar; World Health Organization
**Primary Subject Heading**:	Obstetrics and gynaecology
Secondary Subject Heading:	Global health, Public health, Qualitative research, Patient-centred medicine
Keywords:	PUBLIC HEALTH, QUALITATIVE RESEARCH, Maternal medicine < OBSTETRICS

SCHOLARONE™
Manuscripts

I, the Submitting Author has the right to grant and does grant on behalf of all authors of the Work (as defined in the below author licence), an exclusive licence and/or a non-exclusive licence for contributions from authors who are: i) UK Crown employees; ii) where BMJ has agreed a CC-BY licence shall apply, and/or iii) in accordance with the terms applicable for US Federal Government officers or employees acting as part of their official duties; on a worldwide, perpetual, irrevocable, royalty-free basis to BMJ Publishing Group Ltd ("BMJ") its licensees and where the relevant Journal is co-owned by BMJ to the co-owners of the Journal, to publish the Work in this journal and any other BMJ products and to exploit all rights, as set out in our licence.

The Submitting Author accepts and understands that any supply made under these terms is made by BMJ to the Submitting Author unless you are acting as an employee on behalf of your employer or a postgraduate student of an affiliated institution which is paying any applicable article publishing charge ("APC") for Open Access articles. Where the Submitting Author wishes to make the Work available on an Open Access basis (and intends to pay the relevant APC), the terms of reuse of such Open Access shall be governed by a Creative Commons licence – details of these licences and which Creative Commons licence will apply to this Work are set out in our licence referred to above.

Other than as permitted in any relevant BMJ Author's Self Archiving Policies, I confirm this Work has not been accepted for publication elsewhere, is not being considered for publication elsewhere and does not duplicate material already published. I confirm all authors consent to publication of this Work and authorise the granting of this licence.

For peer review only

Behavioural factors associated with fear of litigation as a driver for the increased use of caesarean sections: A scoping review

Authors

Sarah Elaraby[1, 2]

Elena Altieri[2]

Soo Downe[3]

Joanna Erdman[4]

Sunny Mannava[5]

Gill Moncrieff [3]

BR Shamanna[5]

Maria Regina Torloni[6]

Ana Pilar Betrán[7*]

* Corresponding author: Ana Pilar Betrán; betrana@who.int

Afiliations

1. Community Medicine Department, Alexandria Faculty of Medicine, Egypt
2. Behavioural Insights Unit, World Health Organization, Switzerland
3. University of Central Lancashire, United Kingdom
4. Dalhousie University, Canada
5. University of Hyderabad, India
6. Evidence Based Healthcare Post-Graduate Program, São Paulo Federal University Brazil
7. UNDP/UNFPA/UNICEF/World Bank Special Program of Research, Development and Research Training in Human Reproduction (HRP), Department of Sexual and Reproductive Health and Research, World Health Organization, Switzerland

For peer review only

Abstract

Objective: To explore the behavioural drivers of fear of litigation among healthcare providers influencing CS rates.

Design: Scoping review.

Data sources: We searched MEDLINE, Scopus, and WHO Global Index (January 1, 2001-March 9th, 2022).

Data extraction and synthesis: Data were extracted using a form specifically designed for this review and we conducted content analysis using textual coding for relevant themes. We used the WHO principles for the adoption of a behavioural science perspective in public health developed by the WHO Technical Advisory Group for Behavioural Sciences and Insights to organize and analyse the findings. We used a narrative approach to summarize the findings.

Results: We screened 2968 citations, and 56 were included. Reviewed articles did not use a standard measure of influence of fear of litigation on provider's behaviour. None of the studies used a clear theoretical framework to discuss the behavioural drivers of fear of litigation. We identified 12 drivers under the three domains of the WHO principles: 1) Cognitive drivers: availability bias, ambiguity aversion, relative risk bias, commission bias, and loss aversion bias; 2) social and cultural drivers: patient pressure, social norms, and blame culture; and 3) environmental drivers: legal, insurance, medical and professional, and media. Cognitive biases were the most discussed drivers of fear of litigation, followed by legal environment, and patient pressure.

Conclusions: Despite lack of consensus on a definition or measurement, we found that fear of litigation as a driver for rising CS rates results from a complex interaction between cognitive, social, and environmental drivers. Many of our findings were transferable across

For peer review only

For peer review only - http://bmjopen.bmj.com/site/about/guidelines.xhtml

geographical and practice settings. Behavioural interventions that consider these drivers are crucial to address fear of litigation as part of strategies to reduce CS.

Keywords: Caesarean sections, litigation, behavioural drivers, scoping review

Summary: Strengths and limitations

- We systematically explore global accounts of FoL and associated behavioural factors as drivers for the increased use of CS providing a broad overview of the evidence and identifying research gaps.
- We employed a behavioural approach and the WHO behavioural principles which allowed us to classify drivers within specific domains (cognitive, social and environmental) offering clear pathways for the design of interventions to address these fears.
- We developed a comprehensive search strategy and searched multiple databases but we did not search grey-literature and other informal publications.
- While our interest was the understanding the drivers of medically unnecessary CS, the studies did not differentiate between necessary and unnecessary CS; hence, we reported FoL in relation to "increasing use of CS" rather than "increasing use of unnecessary CS".

For peer review only

Introduction

Over the past decades, rates of caesarean section (CS) have been rising steadily worldwide, in many cases without medical indication (1). Latest available estimates show that worldwide, the average CS rate increased from 6.7% in 1990 to 21% in 2018 (2). The medically unnecessary use of CS impacts the quality of care and is a burden on often stretched healthcare systems (3), in addition to exposing women and offspring to the short- and long-term risks associated with this surgery (4, 5).

Medical litigation can be defined as a legal dispute that involved carrying out a lawsuit or civil action against healthcare providers or a medical entity (6). We use litigation here as an umbrella term to cover a variety of lawsuits including medical malpractice, negligence, and the associated legal liability, risk and settlements.

For peer review only

Fear of litigation (FoL) has been commonly recognized as an influential factor in medical decision making (7). Obstetrics is one of the leading medical specialities in terms of litigation risk and cost (8-11). FoL and defensive medicine (practice wherein a healthcare professional makes decisions out of fear of litigation and not for the benefit of the patients) are commonly cited reasons for performing CS (12-14), sometimes without medical indication (12, 15, 16). Beyond mode of birth (MoB) decision-making, FoL has profound impact maternity care, with studies across the world documenting high rates of defensive medicine (17-22), a decline in the desire to practice obstetrics or early retirement of practicing obstetricians due to litigation concerns (15, 23).

Despite the frequent emphasis on FoL as a driver of increased CS, there is no agreed definition or a standardized tool to measure it, which reduces the ability to assess its role as a driver of CS rates. For the purpose of this review, we rely on explicit mentions of FoL and its consequences in relation to MoB decision-making.

For peer review only - http://bmjopen.bmj.com/site/about/guidelines.xhtml

Behavioural drivers of fear of litigation

Evidence from behavioural science indicates that our decisions and behaviours are often not deliberate and rational, but rather automatic and profoundly influenced by our environment (12, 24). Understanding human behaviour is essential to improve the design and implementation of interventions aiming to influence decision making of providers. To improve understanding of FoL in relation to MoB, and map existing evidence that could inform interventions to optimize the use of CS, we conducted a scoping review asking the following questions: a) how is FoL defined by providers in relation to MoB? b) What are the various behavioural drivers influencing FoL and affecting decision making for MoB?

We used the principles for the adoption of a behavioural science perspective in public health developed by WHO as a guideline to organize and analyse the findings of the review (25). Based on these behavioural principles, we mapped the different types of influences on behaviour, namely cognitive and psychological factors, social and cultural factors, and environment factors driving FoL among providers. The findings of this review further informed this initial mapping.

Methods

The scoping review followed the standard methodology recommended by the Joanna Briggs Institute and is reported in adherence to Preferred Reporting Items for Systematic Reviews and Meta-Analyses for Scoping reviews (PRISMA-ScR) (26) (27).

Data sources and searches

We searched three electronic databases (MEDLINE via PubMed, Scopus, and WHO Global Index) for studies published from January 1, 2001, to March 9th, 2022. The search strategy used two main concepts (and their synonyms): “caesarean section” and “litigation” (detailed

For peer review only - http://bmjopen.bmj.com/site/about/guidelines.xhtml

search strategy in the Supplementary file 1). We also screened the reference lists of reviews and included articles for potentially relevant studies not captured by the electronic search.

Selection criteria

The review included primary research assessing drivers of FoL experienced by providers in relation to MoB or decision-making for CS. We included studies published after 2000 with qualitative, quantitative, or mixed-method designs. Studies from any country or region were eligible for inclusion. Studies were included if they were published in English, Spanish, Portuguese or French. We excluded case reports or case-series (N<6), reports not describing original research (e.g. commentaries, communications, news articles, reviews, etc.), or if they only covered pure legal analysis (i.e. not reporting original data regarding decision-making). Reviews were excluded but their reference list screened for eligible studies. We included articles covering maternity care by providers in any cadre and involved in any health setting or sector.

Study identification and data extraction

Citations retrieved from electronic databases were uploaded into Covidence (Veritas Health Innovation, Melbourne, Australia) and duplicates were excluded. Titles and abstracts were screened in duplicate, and full texts of potentially relevant studies were obtained and assessed by two reviewers independently (SE and APB). Discrepancies were discussed until consensus was reached.

A data-extraction form was specifically designed for this review to capture key aspects of the studies (setting, design, participants, behavioural drivers, and factors affecting FoL; detailed variables extracted in the Supplementary file). We used content analysis to identify emerging themes in the articles, assisted by Atlas.ti 9®, for further analysis of FoL, its drivers and how they influence decision making regarding MoB (28, 29). We developed a qualitative

For peer review only - http://bmjopen.bmj.com/site/about/guidelines.xhtml

codebook informed by previous relevant reviews (12, 24), and the behavioural principles described before (30, 31). One reviewer (SE) supervised by two reviewers (EA and APB) conducted the data extraction and developed the initial codebook. The codebook was iteratively modified as themes emerged inductively during the process of extraction of the included articles in this scoping review, and through continuous team discussions and reflection. We finally cut out redundant themes or those that were either outside the study scope or were included in only one study.

Data synthesis

Using the qualitative coding process, along with the extraction sheet, it was possible to identify and compare different behavioural drivers of FoL within and across studies (28, 29). This started with applying the codebook and extraction sheet to a few studies, and then going through an iterative process, with continuous revision of codes and overarching themes as more studies were analysed. This process was conducted by one reviewer (SE) and discussions among co-authors informed the classification of drivers according to the WHO principles and the definition of how they interact.

In this manuscript, "litigation" and associated terms are used in a non-specific or technical way reflecting their use in the studies reviewed which is often from the perspective of non-legally trained person, namely providers. It also serves as a construct that covers the risk of legal process, and the experience of litigation itself, rather than specific litigation outcomes.

Patient and public involvement

Patients and/or the public were not involved in the design, or conduct, or reporting, or dissemination plans of this scoping review.

For peer review only - http://bmjopen.bmj.com/site/about/guidelines.xhtml

For peer review only

Results

A total of 2968 citation were identified (2944 from electronic databases and 24 from other sources). We selected 440 studies for full-text evaluation and included 56 in this review (Fig 1).

Main characteristics of included studies

Table 1 shows the main characteristics of the 56 included studies. Thirty-four (60.7%) were from high-income countries (HIC) while 22 (39.3%) were from low- and middle-income countries (LMIC). Most (33, 58.9%) had quantitative designs and were published after 2010. All but three included data from a single country; 47 (83.9%) included the perspective of doctors, 12 (21.4%) of midwives, nurses or doulas, 5 (8.9%) of service users and 1 (1.8%) of lawyers. Study details including behavioural drivers reported for each of the 56 studies are presented in Supplementary file 2.

Table 1. Main characteristics of 56 studies included in the scoping review.

Characteristic	N of studies (n=56)	References
Economic category (World Bank classification)		
Low-, lower-, or middle-income countries	22	(13, 14, 17, 32-50)
High income	34	(16, 18, 19, 21, 22, 51-79)
Study design*		
Quantitative	33	(13, 14, 16-19, 21, 22, 44-48, 50, 60-78)
Qualitative	17	(36-43, 52-59, 79)
Mixed methods	6	(32-35, 51)
Year of publication		
2001-2010	17	(16, 19, 33, 37, 46, 51, 53-55, 61, 65, 67-69, 71-73)
2011 to 2022	39	(13, 14, 17, 18, 21, 22, 32, 34-36, 38-45, 47, 48, 52, 56-60, 62-64, 66, 70, 74-79)
Number of countries		
1	53	(13, 14, 16-19, 22, 32-48, 51-55, 58-68, 70-79)
>1	3	(56, 57, 69)
Perspectives included *		
Doctors	47	(13, 14, 16-19, 21, 32-44, 46-64, 68-72, 75-77, 79)

For peer review only - http://bmjopen.bmj.com/site/about/guidelines.xhtml

Midwives, nurses and doulas	12	(33, 40, 49, 50, 52, 54, 58-60, 63, 70, 79)
Service users (pregnant women and their families)	5	(33, 40, 58, 70, 79)
Administrators and health service decision makers	2	(38, 58)
Lawyers	1	(70)
Other (including records)	9	(22, 35, 45, 58, 65-67, 73, 74, 78)

* Articles can include multiple perspectives

Defining and measuring fear of litigation

There was marked heterogeneity in defining or describing FoL in the context of MoB decision-making. Other terms used to imply FoL included liability concerns (22, 38, 45, 63), medico-legal fears (13, 16, 47, 48), and defensive medicine (14, 17, 18, 21, 45, 52, 62, 74, 80). The only tool we found was a risk attitude and fear index developed by Fuglenes, to measure the impact of perceived risk of complaints and malpractice litigation on providers' choice of MoB (16).

Ten studies explored FoL impact on MoB decision-making beyond providers self-report. The proxy measures used to explore FoL included changes to malpractice insurance premiums (49, 62, 67, 73, 74), previous experience with litigation (19, 22, 65, 66), and changes to liability laws (22, 78).

Drivers of fear of litigation

Using the WHO principles (25), we organized the drivers under three domains: 1) Cognitive or psychological drivers; 2) Socio-cultural drivers; and 3) Environmental drivers (Fig 2). For each of the three domains, Table 2 shows the key drivers for FoL identified in this review, their definitions, and frequency and countries from which the findings drive. The number of citations supporting each driver are visualized in Fig 3 showing that the legal environment

For peer review only - http://bmjopen.bmj.com/site/about/guidelines.xhtml

(n=24), the patient pressure (n=19) and the availability bias (n=18) are the most frequently reported drivers in the literature. Findings for each domain are summarized below.

Table 2: Behavioural drivers of fear of litigation (FoL) under the three domains of the WHO principles, definitions, and frequency and countries from which the findings derive.

Domain/ Construct	Definition	Frequency and citations	Countries
**1. Cognitive or psychological drivers** The identified constructs fall under heuristics, which as decision-making strategies rely on a few relevant predictors, while ignoring others. These shortcuts are helpful in some cases but may lead to cognitive biases i.e. a systematic error in judgement when processing information regarding the world around us that eases the cognitive burden of judgement and decision making (24).			
**1.1 Availability bias (in relation to experience with litigation)**	To make judgments of likelihood or frequency based on ease of recall rather than on actual probabilities and pertains to both direct experience, or vicarious through others(24)	18 (14, 16-19, 22, 43, 47, 48, 51, 58, 60, 62, 65-68, 72)	Brazil, Iran, Israel, Norway, Sudan, Turkey, UK, and US
**1.2 Ambiguity aversion: CS "just in case", avoid "worst case scenario"**	The tendency to prefer known or certain probabilities over uncertain probabilities regardless of the actual benefits (24).	16 (13, 14, 34, 36-38, 40, 41, 44, 46, 53-55, 58, 63, 79)	Brazil, Canada, China, Iran, Kenya, Nicaragua, Paraguay, Sweden, Taiwan, Turkey, UK, US,
**1.3 Relative risk bias and beliefs around safety**	Stronger inclination to [a birth mode] when presented with the relative risk than when presented with the same [information] described in terms of the absolute risk (24).	7 (21, 32, 37, 44, 49, 52, 76)	Argentina, Bangladesh, Brazil, Italy, Romania, Spain, and Turkey
**1.4 Commission bias**	Tendency towards action versus inaction.<br>Omission bias: where harmful commissions are usually judged harsher than the corresponding omissions (24).	6 (14, 36, 41, 42, 47, 52)	Brazil, India, Iran, Paraguay, Spain, and Turkey
**1.5 Loss/gain framing or loss aversion bias**	Losses are often perceived as looming larger than corresponding gains (24).	4 (14, 38, 41, 52)	Brazil, Nicaragua, Paraguay, Spain
**2.Social and cultural drivers**			
**2.1 Patient pressure**	Pregnant women, or their families exerting pressure on providers to perform Caesarean delivery on Maternal Request (CDMR), for nonmedical reasons.	19 (32, 33, 35, 38, 39, 41, 43, 50, 51, 53, 55, 56, 61, 69-71, 76, 79, 81).	Bangladesh, Brazil, Canada, France, Germany, India, Iran, Italy, Luxembourg, Netherlands, Nicaragua, Paraguay, Romania, Spain, Sweden, Taiwan, UK, US,
**2.2 Social norms: CS as the new 'normal birth'**	Implicit or explicit rules used by a group and which determine values, beliefs, attitudes and behaviours (82). These norms can be	8 (14, 33, 34, 37, 38, 43, 52, 56)	Brazil, China, Germany, Iran, Ireland, Italy, Nicaragua, Spain

For peer review only - http://bmjopen.bmj.com/site/about/guidelines.xhtml

	descriptive, i.e. what people observe as the typical overt behaviour of others, or injunctive norms, i.e. inference of what is expected of an individual by others (83).		
**2.3 Blame culture**	A culture that does not tolerate error can be a driver to medical excesses, and negative impacts on healthcare quality and patient safety (84, 85).	10 (17, 36, 38, 40, 54-57, 63, 69)	Iran, Ireland, Italy, Netherland, Nicaragua. Sudan, UK, US<br><br>Sweden (mention of absence of blame culture)
**3. Environmental drivers:** Any factor external to the individual that could impact the behaviour. This could include physical environment, or social environment (e.g. medical administrators, peers, patients and their families, etc)			
**3.1 Legal environment**	The legal framework within which providers practice and are faced with liability. This could include tort laws, court systems, admissible evidence, and expert witnesses. This also covers legal mediation, negotiation and settlements outside the court systems	24 (13, 14, 21, 22, 34, 36, 40, 43-45, 47-49, 51, 52, 59, 62-64, 73, 75-78)	Brazil, China, Iran, Italy, Romania, Spain, Sweden, Turkey, UK, US,
**3.2 Malpractice insurance premiums**	The existing insurance mechanisms against malpractice including degree of coverage and premiums	9 (44, 47, 49, 53, 60, 62, 73, 74, 77)	Argentina, Canada, Turkey, US,
**3.3 Medical and professional environment**	The medical system which organizes obstetric practices through medical guidelines, obstetric organizations, or health facility administration	7 (18, 38, 40, 52, 60, 63, 65)	Iran, Israel, Nicaragua, Spain, US,
**3.4 Mass and social media**	This including social and mass media	5 (14, 18, 33, 47, 51)	Brazil, Israel, Turkey, US

1. Cognitive or psychological drivers

1. 1 Availability bias (in relation to experience with litigation)

The impact of malpractice claims was discussed in 18 studies. In these accounts, personal experience of litigation was not essential for inciting FoL, rather 'hearing' about or knowing someone who was sued for malpractice could be enough (18, 48, 65). Malpractice suits can be highly publicized events in the media and within the healthcare system (18, 47) even though their impact is not always tied to adverse judicial outcomes for the provider. The mere

For peer review only - http://bmjopen.bmj.com/site/about/guidelines.xhtml

thought of the experience of being sued due to failure or delay in performing CS, or the hypothesised impact on providers' time and reputation alone influenced decision-making towards a CS (43, 58).

Some authors have quantified the impact of malpractice claims on defensive medicine practices among providers beyond personal experience showing mixed results. Brown et al found that one standard deviation increase in lawsuits was associated with a 1.073 increase in risk of performing CS in United States (19). Also in the United States, Cheng et al reported that clinicians who were sued were more likely to recommend CS than those who had not gone through this experience (17.2 % vs 11.3% respectively, P= 0.008) (62) while Dranove and Watanabe found a modest impact of litigation experience on CS rates of sued obstetricians and their immediate hospital colleagues (65).

1.2 Ambiguity aversion: CS "just in case", avoid "worst case scenario"

Ambiguity, in the form of uncertainty of length and outcome of vaginal birth (VB), was a justification for preferring CS to avoid litigation consequences in 16 studies (13, 14, 34, 36-38, 40, 41, 44, 46, 53-55, 58, 63, 79). This tendency to avoid ambiguity is what leads professionals to perform CS, especially in the context of caesarean delivery on maternal request (CDMR) (55), or vaginal birth after CS (VBAC), or risk of uterine rupture (54, 63).

This ambiguity also may lead providers to shift the responsibility of decision making to the women and their families, rather than giving their professional opinion, as a means of avoiding litigation in case of complications; a CS becomes a sort of insurance policy (55). Some authors note that this behaviour is reinforced by the fact that women and their families are increasingly perceiving adverse events in maternity care as unacceptable (13, 44, 54).

For peer review only - http://bmjopen.bmj.com/site/about/guidelines.xhtml

1.3 Relative risk bias and beliefs around safety

Seven studies explicitly discussed providers' beliefs around the relative safety of CS (32, 37, 44, 49, 50, 52, 76). The inclination to perform CS to avoid litigation risk can be motivated by providers' belief that CS is a safer option than VB, and that CS guarantees the best outcome for mother and child (51). This belief can supersede training, guidelines, or statistical evidence demonstrating the opposite (42).

In LMIC, this belief is further reaffirmed by the reduction in maternal morbidity and mortality due to the introduction –and framing– of CS as a lifesaving intervention in these countries (32). Guidelines and research outlining the benefits of VB could be dismissed by providers in LMIC as perceived not reflecting the realities of their experiences (36).

Even when providers acknowledge the comparative disadvantages of CS to mother and child in the long-term, they may not share this information with their clients (36). As a consequence, the perceived short-term disadvantages of VB are exaggerated or overstated, making VB the less desirable option for providers in light of the risk of litigation, even when VB is objectively safer for women and babies (38).

1.4 Commission bias

Six studies identified drivers that described a tendency to perform CS even if medically unnecessary, just to "do something" rather than wait for uncertain labour events or outcomes (14, 36, 41, 42, 47, 52). With rising rates of CS and lower tolerance for negative maternity care outcomes, providers find waiting for VB challenging, especially if labour is not progressing as expected (36).

Such findings can be categorized as commission bias in maternity care despite evidence that performing CS without medical indication does not reduce risk of lawsuits (86). It contrasts with "omission bias" in other medical specialties (24), where harmful commissions are

For peer review only - http://bmjopen.bmj.com/site/about/guidelines.xhtml

usually judged harsher than the corresponding omissions. In case of complications, maternity care providers perceived that they are more likely to be sued if they failed to perform a CS or delayed it, but unlikely to be sued for performing an unnecessary CS. A study surveying 403 obstetricians in Brazil reported that 67.5% believed VB poses a higher risk of litigation in case of complications, while only 0.5% found that CS had a higher litigation risk (14). A Turkish study found that the perception of VB as a litigation risk was reinforced by wide media coverage of legal cases focusing on delay or failure to perform a CS (47).

1.5 Loss/gain framing or loss aversion bias

Four studies discussed the substantial direct and indirect costs associated with litigation, regardless of the litigation outcome, which underpinned the loss aversion bias pervasiveness in maternity care. Losing a case might result in losing medical licensure, and/or paying large settlements (14, 43, 47). Moreover, regardless of the legal outcome, providers might have to pay higher premiums for malpractice insurance (74), face negative impacts on their reputation (65, 67), and suffer the time and psychological burden of the litigation process itself (43, 62).

2. Socio-cultural drivers

2.1 Patient pressure

Nineteen studies explicitly examined FoL as a driver for providers to agree to a CS based on maternal request and/or pressure by mothers and families (32, 33, 35, 38, 39, 41, 43, 50, 51, 53, 55, 56, 61, 69-71, 76, 79, 81).

Providers' attitudes towards CDMR varied between some providers believing women have a right to choose how they give birth, and others asserting that medical necessity supersedes service users' preferences (71, 76). A study conducted in Italy aimed to explore the perceptions of provider legal liability in case of CDMR among obstetricians, midwives,

For peer review only - http://bmjopen.bmj.com/site/about/guidelines.xhtml

lawyers and patients (70). On average, patients and lawyers rated concession to CDMR as highly appropriate, in contrast to providers who found it inappropriate. Patients and lawyers also rated judicial responsibility of the providers for complications after a CDMR lower than after a VB, suggesting that provider's compliance with maternal choice can be the deciding factor in litigation process. Providers reported that respecting maternal wish for CS is more likely to help avoid litigation in case of complications.

Women's socioeconomic status and education also impacted the providers' perception of their likelihood to be sued in case of complications. Brazilian providers described how more educated women have a higher expectation of care and intolerance to complications during birth, both of which lead to increased risk of litigation if their demands for a CS are not met (33).

2.2 Social Norms: CS as the new 'normal birth'

Eight studies reported providers' concerns that CS is becoming a social norm among women and relatives, since it is widely regarded as safer and 'more modern' than VB (14, 33, 34, 37, 38, 43, 52, 56). In two studies in China and Brazil, providers claimed that CS is expected by service users and families, posing substantial pressure on providers (14, 34). It has been observed that, as countries become more economically stable, families have less children and acceptance or tolerance of adverse events during labour is reduced (18, 34, 43). In these contexts where CS may be seen as the norm, the perception of VB by providers as riskier increases its perceived litigation risk and decreases a provider's ability to defend their performance following a complication (33, 38).

2.3 Blame Culture

Six studies explicitly addressed the effect of blame culture on FoL in relation to CS choice (17, 36, 38, 40, 56, 69), in addition to four studies implicitly discussing effects of blame

For peer review only - http://bmjopen.bmj.com/site/about/guidelines.xhtml

culture (54, 55, 57, 63). This culture can result in hesitancy to even suggest VB as an option for fear of blame in case of complications (55).

In countries where providers expressed less fear of being blamed, they also reported less inclination to perform a CS. In a study on CS decision-making in Sweden, midwives expressed that FoL is not a concern because the medical and legal environment do not foster placing blame, which improves transparency and empowers midwife-led care. Similarly, a study in multiple European countries reported that participants attributed the low rates of VBAC in their countries (Ireland and Italy) to blame culture, in comparison to higher rates in Sweden (56). A qualitative study of three countries with high VBAC rates (Finland, Sweden, and the Netherlands) showed that providers in the Netherlands were concerned with their decline in VBAC rates due to increased risk of litigation and blame in case of complications (57).

3. Environmental drivers

The third principle encompasses the external factors that enable or block a behaviour, including the legal environment, organizational culture, medical bodies policies, guidance, media, and other larger environmental or structural factors that might influence the perceptions of the providers and their decisions (Table 2).

3.1 The legal environment

The legal environment and how it influences maternity care was the focus of 24 studies (13, 14, 21, 22, 34, 36, 40, 43-45, 47-49, 51, 52, 59, 62-64, 73, 75-78). While laws, legal norms and procedures differ by context, we identified common themes across different studies.

The first theme depicted the perceived randomness of legal processes due to the absence of specialized courts, or of a clear legal framework for medical complaints (14, 43), or in some

For peer review only - http://bmjopen.bmj.com/site/about/guidelines.xhtml

cases even a malpractice law (14, 47, 63). These gaps resulted in a perception of lack of fair and specialized legal processes and reinforced providers' perceptions of their vulnerability in case of adverse event, contributing to FoL.

In addition, reliance on expert witnesses in court to testify regarding fault or negligence is a driver for fear of litigation because of the perceived lack of rigour and criteria for who could be considered an expert to assess medical negligence or malpractice or the degree to which they understand the variability in medical opinions (16). In a study in Iran, providers expressed discontent with the lack of training or knowledge of expert witnesses whom they perceived as chosen based on personal relationships, and as having no interest in examining the causes of adverse events, but who rather relied on emotional criteria (43).

The second theme illustrates the lack of clear demarcation within legal systems between malpractice and unavoidable adverse events (44). Providers in some of the included studies perceived that judges, jurors, and expert witnesses assign fault based on empathy with the families' suffering a loss or disability following birth, rather than looking at whether or not this negative outcome was a result of negligence (63). This perception could be reinforced in contexts where limited social or financial support to families and children with disabilities existed, and hence the legal environment seeks to place blame as a way to help the family with the resulting settlement (12).

Once again, this legal environment results in providers considering CS a safer option. If sued after a VB, providers believe they will need to justify not having conducted a CS and whether a CS could have prevented the negative outcome (36, 70). On the other hand, performing a CS when not medically necessary is not recognized as malpractice (16, 36, 70). In addition, depending on the legal context, providers can be at risk of liability for fault even years after the birth. This adds to providers' long-term perceived risk of not performing a CS (43).

For peer review only - http://bmjopen.bmj.com/site/about/guidelines.xhtml

For peer review only

As more cases are settled in favour of families based on adverse outcomes, and more service users are aware of their right to sue if the birth does not go according to their expectations, the legal and financial incentives for litigation are mounting (62). In five studies providers claimed that the settlements themselves have become an incentive for more litigation, and for providers to avoid VB for FoL (14, 34, 43, 52, 67).

3.2 Malpractice insurance premiums

Malpractice insurance premiums and their relationship to FoL was discussed in nine studies (44, 47, 49, 53, 60, 62, 73, 74, 77). A study in the US, found that the likelihood for having a CS increased, and the likelihood for a VD or an instrumental VB decreased, in states where insurance premiums for malpractice exceeded 100,000 USD compared to states where insurance premiums were below 50,000 USD. Inversely, another study found that higher malpractice insurance premiums were not positively associated with recommending CS, but rather that providers who did not know how much they paid for malpractice insurance premiums were less likely to recommend CS, suggesting a possible lesser preoccupation with malpractice risk (62).

3.3 Medical and professional environment

Seven studies discussed medical and professional environments in which providers reported lack of or little protection afforded to them by medical bodies and authorities (18, 38, 40, 52, 60, 63, 65). Medical providers report feeling discouraged or 'demoralized' for having to face these risks without the backing of their colleagues and scientific authorities for their clinical decisions in societies that are becoming increasingly litigious (52). Three articles also included mention that absent or conflicting guidelines regarding CS decision making contributed to their FoL(38, 43, 60). However, administrators in a study in Nicaragua argued that the guidelines exist but are not followed (38).

For peer review only - http://bmjopen.bmj.com/site/about/guidelines.xhtml

3.4 Mass and social media

Media was mentioned as an influential actor in motivating FoL associated with MoB in five studies (14, 18, 33, 47, 51). The sensationalizing of malpractice claims incited fear and frustration among providers. A study in Turkey found that all included providers attested to practicing defensive medicine and being affected by recent high profile malpractice cases in media which resulted in large settlements (47). This media effect, whether traditional or social media, went beyond fearing the risk of litigation, to having a serious impact on providers' mental health, reputation and their approach to their profession (14).

Discussion

This scoping review gathered the evidence available on drivers of FoL when deciding for MoB from the perspective of behavioural theory. The findings highlight the complexity of FoL as a construct that contributes to rising rates of CS across the world. Our use of behavioural principles to develop a conceptual framework for examining drivers FoL at different levels, aided in understanding how they can interact to influence providers' behaviours and choices (Fig 2). This behavioural lens also lent itself to identifying cognitive influences and biases that, while not always explicitly mentioned in the literature, were interpreted through providers' accounts. Identifying such bias could inform the design of behavioural interventions aimed at influencing the driver of FOL.

This review exposed the underlying factors –and the domains they pertain to– which build FoL motivating providers' decision to perform a CS beyond medical need. While our focus was litigation, it is hard to extricate these fears from perceptions of relative safety of CS, fears of disrepute, and consequences of the process of litigation itself rather than its outcome. This more nuanced understanding of fear as a driver for CS is crucial to address unnecessary surgical births globally.

For peer review only - http://bmjopen.bmj.com/site/about/guidelines.xhtml

For peer review only

The review exposed the need for a consensus around measuring FoL in maternity care, given the diversity of proxies used such as malpractice premiums or experience of litigation. Most studies relied on providers self-reported subjective definition, understanding and perception of FoL, without a standardized scale or comparison of actual litigation risk and outcomes. Relying on self-report poses a challenge to reliably quantify and compare the magnitude or burden of FoL, particularly given the multitude of other factors driving the increasing CS rates and which may be beyond the providers' control (87, 88). Using more complex measures such as a 'fear index' as composite measures of FoL as proposed by Fuglenes et al. (16) could provide some value if used in conjunction with other objective measures of litigation risk such as malpractice claims.

Healthcare providers have to rapidly integrate and interpret a complex range of social, psychological, clinical and emotional data relating to both the person they are caring for and the wider context they are working in, in continuously changing situations where often little time is available to make decisions. In maternity care, this is intensified by the fact that the care provider is undertaking this task for both mother and foetus during both pregnancy, labour, and birth, and balancing the sometimes-differing needs of both. Despite existing guidelines, fast decisions require intuitive thinking processes, that jump to the most likely interpretation of and solution to a problem based on patterns of expert knowledge rather than following sequential rules of rationality (89). This review suggests that, in terms of decision for MoB, emotional responses and primarily fear, can be driven by cognitive biases including rational and irrational beliefs regarding safety and risk. These beliefs can supersede training, guidelines or statistical evidence and are deeply entrenched in socio-cultural structures (12, 24). Crucially, the review confirms the influence of sociocultural and environmental factors which encompasses multiple stakeholders including women, families, health professionals,

For peer review only - http://bmjopen.bmj.com/site/about/guidelines.xhtml

healthcare organizations and legal systems. Hence multi-level approaches beyond knowledge-based intervention or training are required to address FoL (90).

This scoping review unveiled the diversity of drivers behind fear as experienced by providers, the domains to which these drivers belong as, the actual risk of litigation and how it manifests in medical practice. Further contextual studies are warranted for in-depth understanding of local dynamics. However, the range of contexts included in the review suggest that underlying concepts revealed in the data synthesis are potentially transferable between quite different contexts.

Our review also highlights the close relationship and interaction between FoL and shifts in norms, particularly through the role and power of media either mainstream or social. Our findings suggest that trial-by-media may be regarded as having a very real immediate influence while the consequences of any litigation process may take years and are ambiguous. The increasing tendency for sensationalist media coverage, and 'click-bait' reinforces this trend (91). Media may also feed into the public perception that CS is the safest and most modern option, and that women should have a choice of elective CS, while downplaying the need to inform people about the risks associated with this choice (as 'fear mongering'). Media pressures may also act to divorce the sense of entitlement to a right to choose from the personal responsibility to accept the consequences for such choices (92). This means that any adverse outcome, even if resulting from personal choices, is deemed to be the fault of someone else, and thus ripe for a litigation case. Further research into how these fears and factors operate and reinforce each other is needed.

Our focus on behavioural drivers for providers means we did not include perspectives of service users and their families, which may be quite different. Although accounts of providers are necessary to understand drivers of FoL, they do not represent an objective measure of the

For peer review only - http://bmjopen.bmj.com/site/about/guidelines.xhtml

strength or frequency of the phenomena described. For example, while 20 of the included studies discuss CDMR as an important driver of increasing CS rates, global rates of CDMR show great variability across regions (0.2%-42%), with upper middle income countries having 11 times the rate of CDMR compared to high income countries (93).

Strengths and limitations

To our knowledge, this is the first review to systematically explore global accounts of FoL and its behavioural drivers in detail providing a broad overview of the evidence on this multidisciplinary topic and identifying research gaps. The use of the WHO behavioural principles enabled us to classify behavioural drivers within specific domains offering clearer pathways for the design of interventions and policies.

Our review has some limitations. We might have overlooked evidence from grey-literature and other informal publications. However, we found and presented data from a range of high-, middle-, and low-income countries. Our classification of cognitive influences on FoL relied on commonly used behavioural constructs and terminologies in medical decision-making literature (12, 24). These constructs may have not been explicitly used by the authors of the studies included to classify their findings, or by participants to describe their own rationale for MoB decision-making. In order to avoid forcing false categories upon the data, we were very conservative in how we classified findings and explicitly sought data that disconfirmed prior assumptions. This was facilitated by the fact that a few distinct themes resonated in the articles across settings. On the other hand, local socio-cultural and environmental influences were often explicitly mentioned in the reviewed articles, and in some cases, were the focus of the study. Although our synthesis showed consistent themes across settings, any initiatives or strategies to address FoL need to be tailored to the specific local context to address the particularities of legislative and health systems and consider how behaviour change

For peer review only - http://bmjopen.bmj.com/site/about/guidelines.xhtml

interventions can be operationalised locally. Lastly, while we were interested in understanding the drivers of medically unnecessary CS, the reviewed literature often did not differentiate between necessary and unnecessary CS, or document the rates of unnecessary CS. Hence, we have approached our study of drivers of FoL in relation to "increasing use of CS" rather than "increasing rates of unnecessary CS".

Conclusion

FoL as a driver for rising CS rates is the result of a complex interaction between cognitive, social, and environment factors. Factors identified across countries include cognitive bias, social pressure from service users, and prevailing norms such as growing intolerance to complications and uncertainty, legal and medical practice environments, and experience with litigation. FoL may be generated less by the actual risk of litigation and more by how the providers perceive this risk and its potential consequences of the process itself independently of the actual legal outcome. Behavioural interventions addressing these drivers are likely to be crucial to address FoL as part of strategies aimed at reducing CS rates.

Acknowledgements

With thanks to Jane Gibbon, Sarah Cordey and Louise Hunt.

Contributorship statement

All authors included on a paper fulfil the criteria of authorship. SE, EA, SD and APB conceived and plan the review. SE and APB screened the studies. SE conducted data extraction and analyses, SE wrote the first draft of the manuscript with support from EA and APB. SD, JE, SM, BRS, MRT made substantial contributions to interpretation, and critically reviewed the manuscript for important intellectual content.

For peer review only - http://bmjopen.bmj.com/site/about/guidelines.xhtml

Competing interest

The authors have declared that no competing interests exist.

Funding

This scoping review was funded by the Behavioural Insights Unit at World Health Organization, the UNDP-UNFPA-UNICEF-WHO-World Bank Special Programme of Research, Development and Research Training in Human Reproduction (HRP), a cosponsored program executed by the World Health Organization (WHO) in the Department of Sexual and Reproductive Health and Research (SRH) and the Gates Grand Challenges 2020: Improving Access to and Use of Safe and Appropriate Cesarean Section (INV-023309). The review was conceived to inform the project Re-JUDGE (https://gcgh.grandchallenges.org/grant/re-judge-reducing-rates-non-medically-indicated-cesarean-sections-through-open-access-multi).

Data sharing statement

All data relevant to the study is included in the article or is available in the supplementary files.

Ethics approval statement

This is a scoping review. No ethics approval was needed.

Figures

Figure 1: Flowchart of process of study identification and inclusion.

Figure 2: Conceptual framework for the classification of drivers of fear of litigation under the three domains defined in the WHO principles.

For peer review only - http://bmjopen.bmj.com/site/about/guidelines.xhtml

Figure 3: Number of citations supporting each driver among 56 citations included in this scoping review.

For peer review only

For peer review only - http://bmjopen.bmj.com/site/about/guidelines.xhtml

References

1. Betran AP, Temmerman M, Kingdon C, Mohiddin A, Opiyo N, Torloni MR, et al. Interventions to reduce unnecessary caesarean sections in healthy women and babies. Lancet. 2018;392(10155):1358-68.
2. Betran AP, Ye J, Moller A-B, Souza JP, Zhang J. Trends and projections of caesarean section rates: global and regional estimates. BMJ Global Health. 2021;6(6):e005671.
3. Gibbons L, Belizan JM, Lauer JA, Betran AP, Merialdi M, Althabe F. Inequities in the use of cesarean section deliveries in the world. American journal of obstetrics and gynecology. 2012;206(4):331. e1-. e19.
4. Steer PJ, Modi N. Elective caesarean sections--risks to the infant. Lancet. 2009;374(9691):675-6.
5. Victora CG, Barros FC. Beware: unnecessary caesarean sections may be hazardous. Lancet. 2006;367(9525):1796-7.
6. Thirumoorthy T. Understanding Medical Negligence and Litigation–Basic for the Medical Professional. BMA News. 2011:12-3.
7. Grant K, White J, Martin J, Haines T. The costs of risk and fear: a qualitative study of risk conceptualisations in allied health resource allocation decision-making. Health, Risk & Society. 2019;21(7-8):373-89.
8. Domingues AP, Moura P, Vieira DN. Obstetric litigation: the importance of the quality of clinical files and its influence on expertise conclusions. Journal of obstetrics and gynaecology : the journal of the Institute of Obstetrics and Gynaecology. 2015;35(2):146-9.
9. Montilla P, Merzagora F, Scolaro E, Requejo J, Ricciardi W, Meli E, et al. Lessons from a multidisciplinary partnership involving women parliamentarians to address the overuse of caesarean section in Italy. BMJ Global Health. 2020;5(2):e002025.
10. Almannie R, Almuhaideb M, Alyami F, Alkhayyal A, Binsaleh S. The status of medical malpractice litigations in Saudi Arabia: Analysis of the annual report. Saudi journal of anaesthesia. 2021;15(2):97-100.
11. Lane J, Bhome R, Somani B. National trends and cost of litigation in UK National Health Service (NHS): a specialty-specific analysis from the past decade. Scottish Medical Journal. 2021;66(4):168-74.
12. Minkoff H. Fear of litigation and cesarean section rates. Semin Perinatol. 2012;36(5):390-4.
13. Kucuk M. Obstetrician perceptions of the causes of high cesarean delivery rates in Turkey. International journal of gynaecology and obstetrics: the official organ of the International Federation of Gynaecology and Obstetrics. 2017;138(1):100-6.
14. Rudey EL, Leal MDC, Rego G. Defensive medicine and cesarean sections in Brazil. Medicine. 2021;100(1):e24176.
15. Adinma J. Litigations and the Obstetrician in Clinical Practice. Ann Med Health Sci Res. 2016;6(2):74-9.
16. Fuglenes D, Oian P, Kristiansen IS. Obstetricians' choice of cesarean delivery in ambiguous cases: is it influenced by risk attitude or fear of complaints and litigation? Am J Obstet Gynecol. 2009;200(1):48 e1-8.
17. Ali AA, Hummeida ME, Elhassan YA, Nabag WO, Ahmed MA, Adam GK. Concept of defensive medicine and litigation among Sudanese doctors working in obstetrics and gynecology. BMC Med Ethics. 2016;17:12.

For peer review only

For peer review only - http://bmjopen.bmj.com/site/about/guidelines.xhtml

18. Asher E, Dvir S, Seidman DS, Greenberg-Dotan S, Kedem A, Sheizaf B, et al. Defensive medicine among obstetricians and gynecologists in tertiary hospitals. PloS one. 2013;8(3):e57108.
19. Brown HS, 3rd. Lawsuit activity, defensive medicine, and small area variation: the case of Cesarean sections revisited. Health economics, policy, and law. 2007;2(Pt 3):285-96.
20. Clark K. Litigation: a threat to obstetric practice? Journal of law and medicine. 2002;9(3):303-22.
21. Fineschi V, Arcangeli M, Di Fazio N, Del Fante Z, Fineschi B, Santoro P, et al. Defensive Medicine in the Management of Cesarean Delivery: A Survey among Italian Physicians. Healthcare (Basel, Switzerland). 2021;9(9).
22. Frakes M. Defensive medicine and obstetric practices. Journal of Empirical Legal Studies. 2012;9(3):457-81.
23. AlDakhil LO. Obstetric and gynecologic malpractice claims in Saudi Arabia: Incidence and cause. Journal of forensic and legal medicine. 2016;40:8-11.
24. Blumenthal-Barby JS, Krieger H. Cognitive biases and heuristics in medical decision making: a critical review using a systematic search strategy. Medical Decision Making. 2015;35(4):539-57.
25. Technical Note from the WHO Technical Advisory Group (TAG) on behavioural insights and science for health. Principles and steps for applying a behavioural perspective to public health: World Health Organization; 2021 [Available from: https://cdn.who.int/media/docs/default-source/documents/bi-tag-technical-note1_principles-and-steps.pdf?sfvrsn=efdefb39_5&download=true.
26. Peters MD, Godfrey CM, Khalil H, McInerney P, Parker D, Soares CB. Guidance for conducting systematic scoping reviews. JBI Evidence Implementation. 2015;13(3):141-6.
27. Tricco AC, Lillie E, Zarin W, O'Brien KK, Colquhoun H, Levac D, et al. PRISMA extension for scoping reviews (PRISMA-ScR): checklist and explanation. Annals of internal medicine. 2018;169(7):467-73.
28. Erlingsson C, Brysiewicz P. A hands-on guide to doing content analysis. Afr J Emerg Med. 2017;7(3):93-9.
29. Graneheim UH, Lindgren BM, Lundman B. Methodological challenges in qualitative content analysis: A discussion paper. Nurse Educ Today. 2017;56:29-34.
30. Bandura A. The evolution of social cognitive theory. Great minds in management. 2005:9-35.
31. Schunk DH, DiBenedetto MK. Motivation and social cognitive theory. Contemporary Educational Psychology. 2020;60:101832.
32. Aminu M, Utz B, Halim A, van den Broek N. Reasons for performing a caesarean section in public hospitals in rural Bangladesh. BMC Pregnancy Childbirth. 2014;14(1):130.
33. Behague DP, Victora CG, Barros FC. Consumer demand for caesarean sections in Brazil: informed decision making, patient choice, or social inequality? A population based birth cohort study linking ethnographic and epidemiological methods. BMJ (Clinical research ed). 2002;324(7343):942-5.
34. Hellerstein S, Feldman S, Duan T. Survey of Obstetric Care and Cesarean Delivery Rates in Shanghai, China. Birth (Berkeley, Calif). 2016;43(3):193-9.
35. Makhija B, Verma D, Mustafa A. A root cause analysis of increasing caesarean section rates in a tertiary care private hospital in North India. International Journal of Reproduction, Contraception, Obstetrics and Gynecology. 2019;8(11):4531.

For peer review only - http://bmjopen.bmj.com/site/about/guidelines.xhtml

36. Bagheri A, Masoudi Alavi N, Abbaszadeh F. Iranian obstetricians' views about the factors that influence pregnant women's choice of delivery method: a qualitative study. Women Birth. 2013;26(1):e45-9.
37. Dias MA, Deslandes SF. [Cesarean sections: risk perception and indication by attending obstetricians in a public maternity hospital in Rio de Janeiro]. Cadernos de saude publica. 2004;20(1):109-16.
38. Colomar M, Cafferata ML, Aleman A, Castellano G, Elorrio EG, Althabe F, et al. Mode of childbirth in low-risk pregnancies: Nicaraguan physicians' viewpoints. Maternal and child health journal. 2014;18(10):2382-92.
39. Doraiswamy S, Billah SM, Karim F, Siraj MS, Buckingham A, Kingdon C. Physician–patient communication in decision-making about Caesarean sections in eight district hospitals in Bangladesh: a mixed-method study. Reproductive health. 2021;18(1):1-14.
40. Firoozi M, Tara F, Ahanchian MR, Latifnejad Roudsari R. Health Care System Barriers to Vaginal Birth after Cesarean Section: A Qualitative Study. Iranian journal of nursing and midwifery research. 2020;25(3):202-11.
41. Goldenberg T, Foster J, Andes KL. Driving birth: Cesarean sections and the medicalization of birth in Gran Asunción, Paraguay. Revista Salud Pública del Paraguay. 2016;6(1):8-15.
42. Peel A, Bhartia A, Spicer N, Gautham M. 'If I do 10–15 normal deliveries in a month I hardly ever sleep at home.' A qualitative study of health providers' reasons for high rates of caesarean deliveries in private sector maternity care in Delhi, India. BMC Pregnancy and Childbirth. 2018;18(1):470.
43. Yazdizadeh B, Nedjat S, Mohammad K, Rashidian A, Changizi N, Majdzadeh R. Cesarean section rate in Iran, multidimensional approaches for behavioral change of providers: a qualitative study. BMC Health Serv Res. 2011;11(1):159.
44. Akpinar F, Kilic F, Ozturk N, Coskun B, Aksar M, Erkaya S. The effect of medical and social conditions on the mode of delivery: a prospective questionnaire-based study applied to 404 Turkish obstetricians. Archives of gynecology and obstetrics. 2021;303(5):1167-74.
45. Chen BK, Yang C-Y. Increased Perception of Malpractice Liability and the Practice of Defensive Medicine. Journal of Empirical Legal Studies. 2014;11(3):446-76.
46. Koigi-Kamau R, Leting PK, Kiarie JN. Perceptions and practices of vaginal birth after Caesarean section among privately practicing obstetricians in Kenya. East African medical journal. 2005;82(12):631-6.
47. Kucuk M. Defensive medicine among obstetricians and gynaecologists in Turkey. Journal of obstetrics and gynaecology : the journal of the Institute of Obstetrics and Gynaecology. 2018;38(2):200-5.
48. Samadi S, Gholizadeh N, Shoar N, Shoar S. Attitudes of Obstetricians toward Cesarean Delivery in Challenging Cases. J Obstet Gynaecol India. 2013;63(5):301-5.
49. Perrotta C, Romero M, Sguassero Y, Straw C, Gialdini C, Righetti N, et al. Caesarean birth in public maternities in Argentina: a formative research study on the views of obstetricians, midwives and trainees. BMJ open. 2022;12(1):e053419.
50. Zhao P, Cai Z, Huang A, Liu C, Li H, Yang S, et al. Why is the labor epidural rate low and cesarean delivery rate high? A survey of Chinese perinatal care providers. PloS one. 2021;16(5):e0251345.
51. Savage W, Francome C. British consultants' attitudes to caesareans. Journal of obstetrics and gynaecology : the journal of the Institute of Obstetrics and Gynaecology. 2007;27(4):354-9.

For peer review only - http://bmjopen.bmj.com/site/about/guidelines.xhtml

52. Bermudez-Tamayo C, Fernandez Ruiz E, Pastor Moreno G, Maroto-Navarro G, Garcia-Mochon L, Perez-Ramos FJ, et al. Barriers and enablers in the implementation of a program to reduce cesarean deliveries. Reprod Health. 2017;14(1):106.
53. Chaillet N, Dube E, Dugas M, Francoeur D, Dube J, Gagnon S, et al. Identifying barriers and facilitators towards implementing guidelines to reduce caesarean section rates in Quebec. Bulletin of the World Health Organization. 2007;85(10):791-7.
54. Kamal P, Dixon-Woods M, Kurinczuk JJ, Oppenheimer C, Squire P, Waugh J. Factors influencing repeat caesarean section: qualitative exploratory study of obstetricians' and midwives' accounts. BJOG : an international journal of obstetrics and gynaecology. 2005;112(8):1054-60.
55. Karlstrom A, Engstrom-Olofsson R, Nystedt A, Thomas J, Hildingsson I. Swedish caregivers' attitudes towards caesarean section on maternal request. Women Birth. 2009;22(2):57-63.
56. Lundgren I, Healy P, Carroll M, Begley C, Matterne A, Gross MM, et al. Clinicians' views of factors of importance for improving the rate of VBAC (vaginal birth after caesarean section): a study from countries with low VBAC rates. BMC Pregnancy Childbirth. 2016;16(1):350.
57. Lundgren I, van Limbeek E, Vehvilainen-Julkunen K, Nilsson C. Clinicians' views of factors of importance for improving the rate of VBAC (vaginal birth after caesarean section): a qualitative study from countries with high VBAC rates. BMC Pregnancy Childbirth. 2015;15(1):196.
58. Munro S, Kornelsen J, Corbett K, Wilcox E, Bansback N, Janssen P. Do Women Have a Choice? Care Providers' and Decision Makers' Perspectives on Barriers to Access of Health Services for Birth after a Previous Cesarean. Birth (Berkeley, Calif). 2017;44(2):153-60.
59. Panda S, Daly D, Begley C, Karlström A, Larsson B, Bäck L, et al. Factors influencing decision-making for caesarean section in Sweden – a qualitative study. BMC Pregnancy and Childbirth. 2018;18(1):377.
60. Barger MK, Dunn JT, Bearman S, DeLain M, Gates E. A survey of access to trial of labor in California hospitals in 2012. BMC Pregnancy Childbirth. 2013;13:83.
61. Bettes BA, Coleman VH, Zinberg S, Spong CY, Portnoy B, DeVoto E, et al. Cesarean delivery on maternal request: obstetrician-gynecologists' knowledge, perception, and practice patterns. Obstet Gynecol. 2007;109(1):57-66.
62. Cheng YW, Snowden JM, Handler SJ, Tager IB, Hubbard AE, Caughey AB. Litigation in obstetrics: does defensive medicine contribute to increases in cesarean delivery? The journal of maternal-fetal & neonatal medicine: the official journal of the European Association of Perinatal Medicine, the Federation of Asia and Oceania Perinatal Societies, the International Society of Perinatal Obstet. 2014;27(16):1668-75.
63. Cox KJ. Providers' perspectives on the vaginal birth after cesarean guidelines in Florida, United States: a qualitative study. BMC Pregnancy Childbirth. 2011;11:72.
64. Deutsch AB, Duncan K, Rajaram L, Salihu HM, Spellacy WN, Belogolovkin V. Cesarean or vaginal delivery for the breech fetus at the threshold of viability: results from a maternal-fetal medicine specialists survey. The journal of maternal-fetal & neonatal medicine: the official journal of the European Association of Perinatal Medicine, the Federation of Asia and Oceania Perinatal Societies, the International Society of Perinatal Obstet. 2011;24(3):475-9.
65. Dranove D, Watanabe Y. Influence and Deterrence: How Obstetricians Respond to Litigation against Themselves and Their Colleagues. American Law and Economics Review. 2010;12(1):69-94.

For peer review only

For peer review only - http://bmjopen.bmj.com/site/about/guidelines.xhtml

66. Durrance CP, Hankins S. Medical Malpractice Liability Exposure and OB/GYN Physician Delivery Decisions. Health services research. 2018;53(4):2633-50.
67. Gimm GW. The impact of malpractice liability claims on obstetrical practice patterns. Health services research. 2010;45(1):195-211.
68. Grant D, McInnes MM. Malpractice experience and the incidence of cesarean delivery: a physician-level longitudinal analysis. Inquiry : a journal of medical care organization, provision and financing. 2004;41(2):170-88.
69. Habiba M, Kaminski M, Da Fre M, Marsal K, Bleker O, Librero J, et al. Caesarean section on request: a comparison of obstetricians' attitudes in eight European countries. BJOG : an international journal of obstetrics and gynaecology. 2006;113(6):647-56.
70. Indraccolo U, Scutiero G, Matteo M, Indraccolo SR, Greco P. Cesarean section on maternal request: should it be formally prohibited in Italy? Annali dell'Istituto superiore di sanita. 2015;51(2):162-6.
71. Kwee A, Cohlen BJ, Kanhai HH, Bruinse HW, Visser GH. Caesarean section on request: a survey in The Netherlands. European journal of obstetrics, gynecology, and reproductive biology. 2004;113(2):186-90.
72. Wells CE. Vaginal birth after cesarean delivery: views from the private practitioner. Semin Perinatol. 2010;34(5):345-50.
73. Yang YT, Mello MM, Subramanian SV, Studdert DM. Relationship between malpractice litigation pressure and rates of cesarean section and vaginal birth after cesarean section. Med Care. 2009;47(2):234-42.
74. Zwecker P, Azoulay L, Abenhaim HA. Effect of fear of litigation on obstetric care: a nationwide analysis on obstetric practice. American journal of perinatology. 2011;28(4):277-84.
75. Mushinski D, Zahran S, Frazier A. Physician behaviour, malpractice risk and defensive medicine: an investigation of cesarean deliveries. Health economics, policy, and law. 2021:1-19.
76. Ionescu CA, Dimitriu M, Poenaru E, Banacu M, Furau GO, Navolan D, et al. Defensive caesarean section: A reality and a recommended health care improvement for Romanian obstetrics. Journal of evaluation in clinical practice. 2019;25(1):111-6.
77. Carrera AM, Sternke EA, Rivera-Viñas JI. A pilot study of the perceptions of actively practicing obstetricians in Puerto Rico: factors that influence decision making in cesarean delivery. Puerto Rico Health Sciences Journal. 2017;36(1).
78. Esposto AG. Tort reform and caesarean deliveries. Applied Economics Letters. 2012;19(12):1171-4.
79. Kuan CI. "Suffering twice": the gender politics of cesarean sections in Taiwan. Med Anthropol Q. 2014;28(3):399-418.
80. Ionescu C. Medical malpractice cases spark debate in Romania. Lancet. 2008;371(9611):462-3.
81. Gau ML, Chang CY, Tian SH, Lin KC. Effects of birth ball exercise on pain and self-efficacy during childbirth: a randomised controlled trial in Taiwan. Midwifery. 2011;27(6):e293-300.
82. Cotterill S, Powell R, Rhodes S, Brown B, Roberts J, Tang MY, et al. The impact of social norms interventions on clinical behaviour change among health workers: protocol for a systematic review and meta-analysis. Systematic Reviews. 2019;8(1):176.
83. Borsari B, Carey KB. Descriptive and injunctive norms in college drinking: a meta-analytic integration. J Stud Alcohol. 2003;64(3):331-41.
84. Khatri N, Brown GD, Hicks LL. From a blame culture to a just culture in health care. Health care management review. 2009;34(4):312-22.

For peer review only - http://bmjopen.bmj.com/site/about/guidelines.xhtml

85. Hoffman JR, Kanzaria HK. Intolerance of error and culture of blame drive medical excess. BMJ (Clinical research ed). 2014;349:g5702.
86. Kessler DP, Summerton N, Graham JR. Effects of the medical liability system in Australia, the UK, and the USA. Lancet. 2006;368(9531):240-6.
87. Long Q, Kingdon C, Yang F, Renecle MD, Jahanfar S, Bohren MA, et al. Prevalence of and reasons for women's, family members', and health professionals' preferences for cesarean section in China: A mixed-methods systematic review. PLoS medicine. 2018;15(10):e1002672.
88. Panda S, Begley C, Daly D. Clinicians' views of factors influencing decision-making for caesarean section: A systematic review and metasynthesis of qualitative, quantitative and mixed methods studies. PLoS One. 2018;13(7):e0200941.
89. Whelehan DF, Conlon KC, Ridgway PF. Medicine and heuristics: cognitive biases and medical decision-making. Irish Journal of Medical Science (1971 -). 2020;189(4):1477-84.
90. Organization WH. WHO recommendations non-clinical interventions to reduce unnecessary caesarean sections: World Health Organization; 2018.
91. O'Keeffe M, Nickel B, Dakin T, Maher CG, Albarqouni L, McCaffery K, et al. Journalists' views on media coverage of medical tests and overdiagnosis: a qualitative study. BMJ open. 2021;11(6):e043991.
92. Moretto G, Walsh E, Haggard P. Experience of agency and sense of responsibility. Consciousness and cognition. 2011;20(4):1847-54.
93. Begum T, Saif-Ur-Rahman KM, Yaqoot F, Stekelenburg J, Anuradha S, Biswas T, et al. Global incidence of caesarean deliveries on maternal request: a systematic review and meta-regression. BJOG : an international journal of obstetrics and gynaecology. 2021;128(5):798-806.

For peer review only

For peer review only - http://bmjopen.bmj.com/site/about/guidelines.xhtml

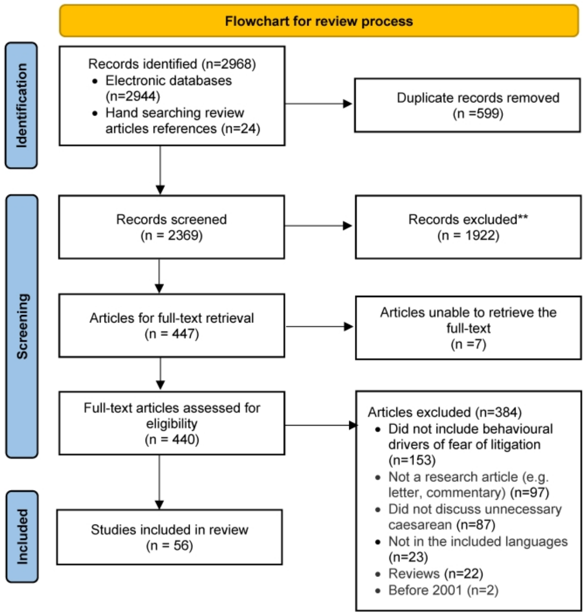

Fig 1: Flowchart of process of study identification and inclusion.

548x710mm (236 x 236 DPI)

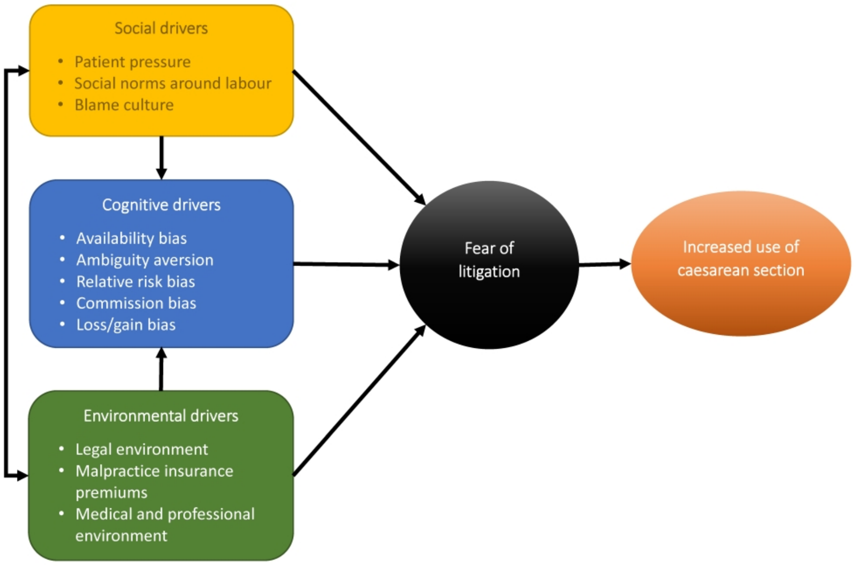

Fig 2: Conceptual framework for the classification of drivers of fear of litigation under the three domains defined in the WHO principles.

710x548mm (236 x 236 DPI)

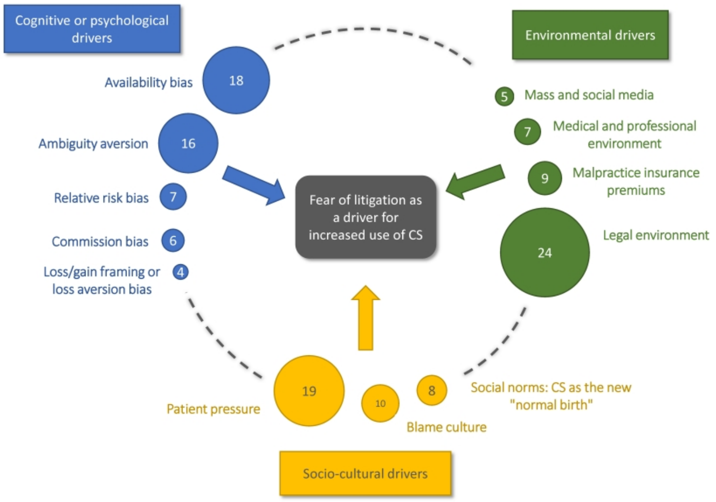

Fig 3: Number of citations supporting each driver among 56 citations included in this scoping review.

861x484mm (236 x 236 DPI)

Supplementary file 1

Search Strategy

PubMed

("OBSTETRICS/LEGISLATION AND JURISPRUDENCE"[MESH] OR "CESAREAN SECTION"[MESH] OR "CESAREAN SECTION"[TIAB] OR "CESAREAN SECTION"[TW] OR "CESAREAN SECTIONS"[TIAB] OR "CESAREAN SECTIONS"[TW] OR "CAESAREAN"[TW] OR "DELIVERY, ABDOMINAL" [TIAB] OR "ABDOMINAL DELIVERIES" [TIAB] OR "DELIVERIES, ABDOMINAL" [TIAB] OR "CAESAREAN SECTION" [TIAB] OR "CAESAREAN SECTIONS" [TIAB] OR "ABDOMINAL DELIVERY" [TIAB] OR "C-SECTION" [TIAB] OR "C SECTION" [TIAB] OR "C-SECTIONS" [TIAB] OR "POSTCESAREAN SECTION"[TIAB])

AND

(LEGIS OR "OBSTETRICS/LEGISLATION AND JURISPRUDENCE"[MESH] OR "JURISPRUDENCE"[MESH] OR "COURT DECISION*" [TIAB] OR "LAW" [TIAB] OR "LAW"[TW] OR "LAWS"[TW] OR "LEGAL*"[TW] OR "LAWY*" [TW] OR "LITIGATION"[TW] OR "LITIGATIONS"[TW] OR "LITIGAT*"[TW] OR "LEGAL CONSEQ*"[TIAB] OR "MEDICAL JURISPRUDENCE" [TW] OR "DEFENSIVE MEDICINE"[MESH] OR "DEFENSIVE PRACTICE*"[TIAB] OR "NEGOTIATING"[MESH] OR "NEGOTIAT*" [TIAB] OR "MEDIATION" [TIAB] OR "MEDIATING" [TIAB] OR "ARBITRATING" [TIAB] OR "ARBITRATION*" [TIAB] OR "CONFLICT RESOLUTION" [TIAB] OR "CONFLICT RESOLUTIONS" [TIAB] OR "RESOLUTION, CONFLICT" [TIAB] OR "MEDICO-LEGAL"[TIAB] OR "VIOLENCE"[TIAB] OR "CASE LAW"[TIAB] OR "LEGAL CASE*"[TIAB] OR "COURT CASE"[TIAB] OR "MALPRACTICE"[MESH] OR "MALPRACTICE"[TIAB] OR "NEGLIGENCE"[TIAB] OR "NEGLIGEN*"[TIAB] OR "MALFEASANCE"[TIAB] OR "MISCONDUCT"[TW] OR "PROFESSIONAL DISCIPLINE"[TW] OR "PATIENT ADVOCACY" [TW] OR "PATIENT RIGHTS"[TW] OR "LIABILITY"[TIAB])

AND

AND PUBYEAR > 2000

Scopus

(TITLE-ABS-KEY(("Caesarean section" OR "Cesarean Section" OR "Caesarean sections" OR "Cesarean Sections" OR "C-section" OR "C-sections" OR "Abdominal delivery" OR "Abdominal deliveries"))

For peer review only - http://bmjopen.bmj.com/site/about/guidelines.xhtml

AND

TITLE-ABS-KEY(("Jurisprudence" OR "Court Decision*" OR "Law" OR "law" OR "laws" OR "legal*" OR "lawy*" OR "litigation" OR "litigations" OR "litigat*" OR "legal conseq*" OR "Medical Jurisprudence" OR "Defensive Medicine" OR "Defensive Practice*" OR "Negotiating" OR "Negotiat*" OR "Mediation" OR "Mediating" OR "Arbitrating" OR "Arbitration" OR "Arbitrations" OR "Conflict Resolution" OR "Conflict Resolutions" OR "medico-legal" OR "Violence" OR "case law" OR "legal case*" OR "court case" OR "Malpractice" OR "malpractice" OR "Negligence" OR "negligen*" OR "malfeasance" OR "misconduct" OR "professional discipline" OR "patient advocacy" OR "patient rights")))

AND PUBYEAR > 2000

WHO Global Index Medicus

("Caesarean section" OR "Cesarean Section" OR "Caesarean sections" OR "Cesarean Sections" OR "C-section" OR "C-sections" OR "Abdominal delivery" OR "Abdominal deliveries")

AND

("Jurisprudence" OR "Court Decision*" OR "Law" OR "law" OR "laws" OR "legal*" OR "lawy*" OR "litigation" OR "litigations" OR "litigat*" OR "legal conseq*" OR "Medical Jurisprudence" OR "Defensive Medicine" OR "Defensive Practice*" OR "Negotiating" OR "Negotiat*" OR "Mediation" OR "Mediating" OR "Arbitrating" OR "Arbitration" OR "Arbitrations" OR "Conflict Resolution" OR "Conflict Resolutions" OR "medico-legal" OR "Violence" OR "case law" OR "legal case*" OR "court case" OR "Malpractice" OR "malpractice" OR "Negligence" OR "negligen*" OR "malfeasance" OR "misconduct" OR "professional discipline" OR "patient advocacy" OR "patient rights")

AND

PUBYEAR > 2000

Detailed inclusion and exclusion criteria

Inclusion criteria

- Focus on caesarean section (not broadly obstetrics).
- Include fear of litigation (broadly defined) in the findings
- Include analysis of empirical data
- Include peer-reviewed articles
- Descriptive or experimental design

For peer review only - http://bmjopen.bmj.com/site/about/guidelines.xhtml

  - Can include both quantitative designs/data:
    - *E.g.* cross sectional, cohort, non-randomized controlled trials; program evaluations; interrupted time-series; pre-post designs; pilot studies; etc
    - *N > 6*
  - Qualitative designs/data
    - In-depth interviews; focus groups; key-informant interviews
    - *N > 1*
  - And mixed methods
- Include drivers of fear of litigation (i.e. goes beyond mere mention of its existence).
  - These can include behavioural determinants or drivers.
  - Can include stakeholders or other actors influencing fear of litigation.
- Publication language: English, Spanish, Portuguese, French, or German
- Published between 2001 and 2021

Exclusion criteria

- Case-studies or case-series
- Studies that report on a study or program where main outcomes are NOT related to unnecessary caesarean sections (broadly discuss obstetric malpractice).
- Reports or articles that do not describe original research (e.g. Abstracts, communications, book chapters, news articles, etc)
- Studies with no mention of fear of litigation or its drivers (beyond its presence based on previous literature)
- Pure legal analysis that does not discuss healthcare decision-making
- Review papers with no substantial analysis of fear of litigation (but screened references for more studies)
- Publication language other than English, Spanish, Portuguese, German or French.
- Published before 2001

Variables collected (data-extraction form)

The following variables were extracted in an excel form specifically designed for this scoping review:

- Author, citation
- Year of the study
- Topic/domain
- Country or countries
- Study design
- Data type
- Sample size
- Type of participant
- Participant characteristics

For peer review only - http://bmjopen.bmj.com/site/about/guidelines.xhtml

- Behavioural drivers
- For each behavioural driver, how the behavioural drivers were assessed
- For each behavioural driver, rational or justification for effect on fear of litigation
- For each behavioural driver, other actors in relation with the behavioural driver
- Key limitations
- Key strengths
- Quotes
- Other notes

For peer review only

For peer review only - http://bmjopen.bmj.com/site/about/guidelines.xhtml

Supplementary file 2: Study details including behavioural drivers reported by each study.

Author, year	Country	Method/data type	Participants and sample size	Behavioural drivers for CS
Akpinar, 2021 (1)	Turkey	Survey<br><br>Quantitative data	404 Obstetricians and Gynaecological administrators and practitioners	Ambiguity aversion<br>Beliefs around safety<br>Legal environment<br>Limited coverage of liability insurance
Ali, 2016 (2)	Sudan	Survey<br><br>Quantitative data	117 OBGYN (42.7% consultants, 34.2% registrars and 23.1% specialists)	Availability bias<br>Blame Culture
Aminu et al., 2014 (3)	Bangladesh	Mixed methods (retrospective review of case notes and Key informant interviews with service providers)	18 OBGYN and case notes of 530 women who had CS in 5 Public Hospitals	Patient Pressure<br>Relative risk bias and Beliefs around safety
Asher, 2013 (4)	Israel	Survey<br><br>Quantitative data	117 board certified physicians and residents in OBGYN departments at tertiary hospitals	Availability bias<br>Medical and professional environment<br>Media
Bagheri, 2013 (5)	Iran	Qualitative data	18 OBGYN	Ambiguity aversion<br>Legal environment
Berger, 2012 (6)	United States	Survey<br><br>Quantitative data	Nurses at 243 civilian California birth hospitals	Availability bias<br>Medical and professional environment<br>Malpractice insurance premiums
Bastos-Dias, 2004 (7)	Brazil	Qualitative data	OBGYNs practicing in one public maternity in Rio de Janeiro, caring for women with low education	Beliefs around safety<br>Ambiguity aversion<br>Social norms
Behague, 2002 (8)	Brazil	Mixed methods	80 mothers from a birth cohort of 5304 women who gave birth in 1993 in any of the hospitals in Pelotas, Brazil. As well as 19 clinicians (six	Patient Pressure<br>Media

For peer review only - http://bmjopen.bmj.com/site/about/guidelines.xhtml

			OBGYN, six paediatricians, four general practitioners, and three nurses)	
Bermúdez-Tamayo, 2017 (9)	Spain	Qualitative data	14 OBGYN and 14 nurse-midwives (NMW) who work for Spain's National Healthcare System	Legal environment<br>Commission bias<br>Loss/gain framing or loss aversion bias<br>Social norms<br>Beliefs around safety
Bettes, 2007 (10)	United States	Survey<br><br>Quantitative data	699 OBGYN	Patient pressure
Brown, 2007 (11)	United States	Secondary analysis<br><br>Quantitative data	Hospital discharges data and Insurance Closed Claim File data. Analysis was conducted using a random sample, with replacement of 20,000 births out of the total birth population of 260,292	Availability bias
Carrera, 2017 (12)	United States (Puerto Rico)	Survey<br><br>Quantitative data	62 OBGYN	Patient pressure<br>Legal System<br>Availability bias<br>Malpractice insurance/premiums
Chaillet, Dube (13)	Canada	Qualitative data	10 Focus group discussions (FGDs) and 6 in-depth interviews (IDIs)	Patient pressure<br>Ambiguity aversion<br>Medical and professional environment
Chen and Yang (14)	Taiwan	Panel analysis<br><br>Quantitative data	Random subsamples of population of outpatient and inpatient claim in Taiwan (1997-2005) out of 1 million random individual medical claim histories	Legal environment
Cheng, 2014 (15)	United States	Survey<br><br>Quantitative data	1486 clinicians (general OBGYN, Maternal-Foetal Medicine specialists, family medicine physicians and clinical nurse midwives), who practice obstetrics in the US and who were registered as active members of the American Medical Association (AMA).	Availability bias<br>Malpractice insurance/premiums<br>Legal environment

For peer review only - http://bmjopen.bmj.com/site/about/guidelines.xhtml

Colomar, 2014 (16)	Nicaragua	Qualitative data	4 FGDs, and 17 IDIs with OBGYN and medical decision makers	Patient Pressure<br>Ambiguity aversion<br>Loss/gain framing or loss aversion bias<br>Medical and professional environment
Cox, 2011 (17)	United States	Semi-structured interviews<br><br>Qualitative data	11 obstetricians, 12 midwives, and a hospital administrator (n = 24)	Legal environment<br>Ambiguity aversion<br>Blame Culture
Deutch, 2011 (18)	United States	Survey<br><br>Quantitative data	510 OBGYN (Society for Maternal–Foetal Medicine (SMFM) members)	Legal environment
Doraiswany, 2021 (19)	Bangladesh	IDIs and Labour observations<br><br>Qualitative data	16 physicians and 32 women who delivered by CS	Patient pressure<br>Risk aversion bias (loss gain framing)
Dranove, 2010 (20)	United States	Quantitative record analysis	Using patient-level hospital discharge data and matching physician license of presiding OBGYN to data of closed medical malpractice claims. Examined data of 1539 unique physicians who carried out 98% of the included birth records.	Availability bias<br>Medical and professional environment
Durrance, 2018(21)	United States	Quantitative data	Around 2 million hospital deliveries linked to physician malpractice claim histories (around 2300 doctors) between 1994 and 2010	Availability bias
Esposto, 2012 (22)	United States	Quantitative data	Panel data of CS rates in three years (1987, 1990 and 1993) in 50 states	Legal System (tort reform)
Fineschi, 2021 (23)	Italy	Quantitative data	168 OBGYN	Legal system<br>Patient pressure<br>Relative risk bias and beliefs around safety
Firoozi et al, 2020 (24)	Iran	IDIs and an FGD<br><br>Qualitative data	26 IDIs with maternity care providers (OBGYN and Midwives) and mothers with prior CS	Legal environment<br>Ambiguity aversion<br>Blame culture

For peer review only - http://bmjopen.bmj.com/site/about/guidelines.xhtml

Frakes, 2012 (25)	United States	Secondary quantitative data	Using data on physician behaviour from the 1979–2005 National Hospital Discharge Surveys (NHDS),	Availability bias (Experience with litigation)<br>Legal environment (tort reform)
Fuglenes, 2009 (26)	Norway	Survey<br><br>Quantitative data	716 OBGYN	Perceived risk of litigation (Fear Index)
Gimm, 2010 (27)	United States	Secondary data<br><br>Panel Analysis	Secondary data from the 1992–2000 Florida Hospital Inpatient Discharge File, the Florida Medical Professional Liability Insurance Claims File, and the American Medical Association’s Master File on physician characteristics.	Availability bias (Experience with litigation)
Goldenberg, 2016 (28)	Paraguay	IDIs<br><br>Qualitative data	10 OBGYN	Commission bias<br>Loss/gain framing or loss aversion bias<br>Ambiguity aversion<br>Patient pressure
Grant, 2004 (29)	United States	Panel Analysis<br><br>Quantitative data	835 obstetricians who performed at least 10 births in each year and had all the information on physician characteristics	Availability bias
Habiba, 2006 (30)	Luxembourg, Netherlands and Sweden, France, Germany, Italy, Spain and UK	self-administered questionnaire<br><br>Quantitative data	One hundred and five units Neonatal Intensive Care Unit (NICU) associated maternity units, and 1530 obstetricians participated in the study in eight European countries.	Patient pressure
Hellerstein, 2016 (31)	China	Survey and qualitative observations<br><br>Mixed methods data	Descriptive structured survey of obstetric services with standardized interview with obstetricians and qualitative observation of obstetric services in six different Shanghai obstetric facilities	Patient (and family) pressures<br>Ambiguity aversion<br>Social norms regarding birth

For peer review only - http://bmjopen.bmj.com/site/about/guidelines.xhtml

For peer review only

.				
Indraccolo, 2015 (32)	Italy	Survey<br><br>Quantitative data	36 OBGYNs, 42 midwives, 22 lawyers and 25 patients	Patient pressure
Ionsecu, 2018 (33)	Romania	Survey<br><br>Quantitative data	73 OBGYNs	Patient pressure<br>Relative risk bias and beliefs around safety<br>Legal environment
Kamal, 2005 (34)	United Kingdom	IDIs<br><br>Qualitative data	Trainee and consultant obstetricians, and community and hospital-based midwives involved in the care of women in pregnancy and labour in Leicestershire,	Ambiguity aversion<br>Blame Culture
Karlstro¨m, 2009 (35)	Sweden	FGDs<br><br>Qualitative data	5 FGDs where 16 midwives and 9 obstetricians participated.	Ambiguity aversion<br>Patient pressure
Koigi-Kamau, 2005 (36)	Kenya	Survey<br><br>Quantitative data	64 obstetricians with individual private practices (i.e., independent of institutional policies) from the first major towns in Kenya – namely Nairobi, Mombasa, Kisumu, Nakuru and Eldoret.	Ambiguity aversion
Kuan (37)	Taiwan	Multiple Qualitative methods	Interviews with eight obstetricians, two nurses, a doula, a midwife, and 34 pregnant women along with document review, and ethnographic observations	Patient pressure<br>Ambiguity aversion
Kucuk (38)	Turkey	Survey<br><br>Quantitative data	100 obstetricians who responded to the survey.	Legal environment<br>Ambiguity aversion
Kucuk (39)	Turkey	Survey<br><br>Quantitative data	108 practicing OBGYNs	Availability bias<br>Malpractice insurance/premiums<br>Legal environment

For peer review only - http://bmjopen.bmj.com/site/about/guidelines.xhtml

Kwee, Cohlen (40)	Netherlands	Survey<br><br>Quantitative data	583 OBGYN (80% consultants and 20% were senior registrar	Patient pressure
Lundgren, 2016 (41)	Ireland, Italy, and Germany	FGDs<br><br>Qualitative methods	FGDs were held in Ireland, Italy and Germany. In total 71 clinicians participated in nine FGDs.	Social Norms regarding birth<br>Blame culture<br>Patient pressure
Lundgren, 2015 (42)	Finland, Sweden, and the Netherlands	IDIs (face to face and over the telephone)<br><br>FGFs<br><br>Qualitative methods	26 midwives and 18 obstetricians.	Legal System<br>Blame culture (absence of)<br>Media
Makhija et al., 2019 (43)	India	Mixed methods (birth data and IDIs)	20 consultant OBGYNs	Patient pressure
Munro, 2017 (44)	Canada	Qualitative methods	35 Family physicians, midwives, obstetricians, nurses, anaesthetists, and health service decision makers recruited from 3 rural and 2 urban Canadian communities	Availability bias<br>Ambiguity aversion
Mushiniski, 2021 (45)	United States	Quantitative data		Legal environment
Panda, Daly (46)	Sweden	FGDs<br><br>Qualitative methods	4 FGDs with OBGYNs and Midwives involved in birth decision making	Legal environment
Peel, Bhartia (47)	India	IDIs<br><br>Qualitative methods	14 OBGYN and providers involved in maternity care	Relative Risk bias and beliefs around safety<br>Commission bias
Perrotta, 2021 (48)	Argentina	Key informant interviews (KII) and Surveys<br><br>Mixed methods	680 surveys and 26 KIIs of heads of hospital departments, OBGYN, midwives and residents	Legal system<br>Relative Risk bias and beliefs around safety<br>Malpractice insurance/premiums and coverage

For peer review only - http://bmjopen.bmj.com/site/about/guidelines.xhtml

Rudey, 2021 (49)	Brazil	Survey<br><br>Quantitative data	403 General obstetricians who are members of premier Brazilian professional associations of gynaecology and obstetrics	Commission bias<br>Loss/gain framing or loss aversion bias<br>Ambiguity aversion<br>Availability bias<br>Legal environment<br>Media
Samadi, 2013 (50)	Iran	Survey<br><br>Quantitative data	75 OBGYN	Availability bias (Experience with litigation)<br>Legal environment
Savage, 2007 (51)	United Kingdom	Mixed methods	151 OBGYN and clinical directors in OBGYN listed by the royal college of OBGTN in British Isles	Experience with litigation (financial and time drain)<br>Legal environnement<br>Patient pressure<br>Media
Wells, 2010 (52)	United States	Survey<br><br>Quantitative data	458 practicing obstetricians	Availability bias (Experience with litigation)
Yang, 2009 (53)	United States	Quantitative data	52 million recorded births state-level longitudinal mixed-effects regression models to examine data from the Natality Detail File on births in the United States (1991–2003)	Malpractice insurance/premiums<br>Legal environment
Yazdizadeh, 2011 (54)	Iran	IDIs and document analysis<br><br>Qualitative data	26 providers from various public and private hospitals responding to the ministry's C-section reduction interventions. Participants included hospital director, obstetricians and midwives.	Availability bias (Experience with litigation)<br>Legal environment (judges' capability, unrealistic expectation from medical professionals, and large settlements)<br>Patient pressure
Zhao, 2021(55)	China	Survey<br><br>Quantitative data	606/1412 anesthesiologists , 495 obstetricians, 219 midwives and labor & delivery nurses, 1412 hospital administrators and 59 specialties unspecified.	Legal environment<br>Patient pressure<br>Relative risk bias and beliefs around safety

For peer review only

Zwecker, 2011 (56)	United States	Quantitative record data	890,266 records of women who delivered and malpractice premium data in 37 states in 2006	Malpractice insurance/premiums

For peer review only

For peer review only - http://bmjopen.bmj.com/site/about/guidelines.xhtml

References

1. Akpinar F, Kilic F, Ozturk N, Coskun B, Aksar M, Erkaya S. The effect of medical and social conditions on the mode of delivery: a prospective questionnaire-based study applied to 404 Turkish obstetricians. Archives of gynecology and obstetrics. 2021;303(5):1167-74.
2. Ali AA, Hummeida ME, Elhassan YA, Nabag WO, Ahmed MA, Adam GK. Concept of defensive medicine and litigation among Sudanese doctors working in obstetrics and gynecology. BMC Med Ethics. 2016;17:12.
3. Aminu M, Utz B, Halim A, van den Broek N. Reasons for performing a caesarean section in public hospitals in rural Bangladesh. BMC Pregnancy Childbirth. 2014;14(1):130.
4. Asher E, Dvir S, Seidman DS, Greenberg-Dotan S, Kedem A, Sheizaf B, et al. Defensive medicine among obstetricians and gynecologists in tertiary hospitals. PloS one. 2013;8(3):e57108.
5. Bagheri A, Masoudi Alavi N, Abbaszadeh F. Iranian obstetricians' views about the factors that influence pregnant women's choice of delivery method: a qualitative study. Women Birth. 2013;26(1):e45-9.
6. Barger MK, Dunn JT, Bearman S, DeLain M, Gates E. A survey of access to trial of labor in California hospitals in 2012. BMC Pregnancy Childbirth. 2013;13:83.
7. Dias MA, Deslandes SF. [Cesarean sections: risk perception and indication by attending obstetricians in a public maternity hospital in Rio de Janeiro]. Cadernos de saude publica. 2004;20(1):109-16.
8. Behague DP, Victora CG, Barros FC. Consumer demand for caesarean sections in Brazil: informed decision making, patient choice, or social inequality? A population based birth cohort study linking ethnographic and epidemiological methods. BMJ (Clinical research ed). 2002;324(7343):942-5.
9. Bermudez-Tamayo C, Fernandez Ruiz E, Pastor Moreno G, Maroto-Navarro G, Garcia-Mochon L, Perez-Ramos FJ, et al. Barriers and enablers in the implementation of a program to reduce cesarean deliveries. Reprod Health. 2017;14(1):106.
10. Bettes BA, Coleman VH, Zinberg S, Spong CY, Portnoy B, DeVoto E, et al. Cesarean delivery on maternal request: obstetrician-gynecologists' knowledge, perception, and practice patterns. Obstet Gynecol. 2007;109(1):57-66.
11. Brown HS, 3rd. Lawsuit activity, defensive medicine, and small area variation: the case of Cesarean sections revisited. Health economics, policy, and law. 2007;2(Pt 3):285-96.
12. Carrera AM, Sternke EA, Rivera-Viñas JI. A pilot study of the perceptions of actively practicing obstetricians in Puerto Rico: factors that influence decision making in cesarean delivery. Puerto Rico Health Sciences Journal. 2017;36(1).
13. Chaillet N, Dube E, Dugas M, Francoeur D, Dube J, Gagnon S, et al. Identifying barriers and facilitators towards implementing guidelines to reduce caesarean section rates in Quebec. Bulletin of the World Health Organization. 2007;85(10):791-7.
14. Chen BK, Yang C-Y. Increased Perception of Malpractice Liability and the Practice of Defensive Medicine. Journal of Empirical Legal Studies. 2014;11(3):446-76.
15. Cheng YW, Snowden JM, Handler SJ, Tager IB, Hubbard AE, Caughey AB. Litigation in obstetrics: does defensive medicine contribute to increases in cesarean delivery? The journal of maternal-fetal & neonatal medicine : the official journal of the European Association of Perinatal Medicine, the Federation of Asia and Oceania Perinatal Societies, the International Society of Perinatal Obstet. 2014;27(16):1668-75.
16. Colomar M, Cafferata ML, Aleman A, Castellano G, Elorrio EG, Althabe F, et al. Mode of childbirth in low-risk pregnancies: Nicaraguan physicians' viewpoints. Maternal and child health journal. 2014;18(10):2382-92.
17. Cox KJ. Providers' perspectives on the vaginal birth after cesarean guidelines in Florida, United States: a qualitative study. BMC Pregnancy Childbirth. 2011;11:72.

For peer review only - http://bmjopen.bmj.com/site/about/guidelines.xhtml

18. Deutsch AB, Duncan K, Rajaram L, Salihu HM, Spellacy WN, Belogolovkin V. Cesarean or vaginal delivery for the breech fetus at the threshold of viability: results from a maternal-fetal medicine specialists survey. The journal of maternal-fetal & neonatal medicine : the official journal of the European Association of Perinatal Medicine, the Federation of Asia and Oceania Perinatal Societies, the International Society of Perinatal Obstet. 2011;24(3):475-9.
19. Doraiswamy S, Billah SM, Karim F, Siraj MS, Buckingham A, Kingdon C. Physician–patient communication in decision-making about Caesarean sections in eight district hospitals in Bangladesh: a mixed-method study. Reproductive health. 2021;18(1):1-14.
20. Dranove D, Watanabe Y. Influence and Deterrence: How Obstetricians Respond to Litigation against Themselves and Their Colleagues. American Law and Economics Review. 2010;12(1):69-94.
21. Durrance CP, Hankins S. Medical Malpractice Liability Exposure and OB/GYN Physician Delivery Decisions. Health services research. 2018;53(4):2633-50.
22. Esposto AG. Tort reform and caesarean deliveries. Applied Economics Letters. 2012;19(12):1171-4.
23. Fineschi V, Arcangeli M, Di Fazio N, Del Fante Z, Fineschi B, Santoro P, et al. Defensive Medicine in the Management of Cesarean Delivery: A Survey among Italian Physicians. Healthcare (Basel, Switzerland). 2021;9(9).
24. Firoozi M, Tara F, Ahanchian MR, Latifnejad Roudsari R. Health Care System Barriers to Vaginal Birth after Cesarean Section: A Qualitative Study. Iranian journal of nursing and midwifery research. 2020;25(3):202-11.
25. Frakes M. Defensive medicine and obstetric practices. Journal of Empirical Legal Studies. 2012;9(3):457-81.
26. Fuglenes D, Oian P, Kristiansen IS. Obstetricians' choice of cesarean delivery in ambiguous cases: is it influenced by risk attitude or fear of complaints and litigation? Am J Obstet Gynecol. 2009;200(1):48 e1-8.
27. Gimm GW. The impact of malpractice liability claims on obstetrical practice patterns. Health services research. 2010;45(1):195-211.
28. Goldenberg T, Foster J, Andes KL. Driving birth: Cesarean sections and the medicalization of birth in Gran Asunción, Paraguay. Revista Salud Pública del Paraguay. 2016;6(1):8-15.
29. Grant D, McInnes MM. Malpractice experience and the incidence of cesarean delivery: a physician-level longitudinal analysis. Inquiry : a journal of medical care organization, provision and financing. 2004;41(2):170-88.
30. Habiba M, Kaminski M, Da Fre M, Marsal K, Bleker O, Librero J, et al. Caesarean section on request: a comparison of obstetricians' attitudes in eight European countries. BJOG : an international journal of obstetrics and gynaecology. 2006;113(6):647-56.
31. Hellerstein S, Feldman S, Duan T. Survey of Obstetric Care and Cesarean Delivery Rates in Shanghai, China. Birth (Berkeley, Calif). 2016;43(3):193-9.
32. Indraccolo U, Scutiero G, Matteo M, Indraccolo SR, Greco P. Cesarean section on maternal request: should it be formally prohibited in Italy? Annali dell'Istituto superiore di sanita. 2015;51(2):162-6.
33. Ionescu CA, Dimitriu M, Poenaru E, Banacu M, Furau GO, Navolan D, et al. Defensive caesarean section: A reality and a recommended health care improvement for Romanian obstetrics. Journal of evaluation in clinical practice. 2019;25(1):111-6.
34. Kamal P, Dixon-Woods M, Kurinczuk JJ, Oppenheimer C, Squire P, Waugh J. Factors influencing repeat caesarean section: qualitative exploratory study of obstetricians' and midwives' accounts. BJOG : an international journal of obstetrics and gynaecology. 2005;112(8):1054-60.
35. Karlstrom A, Engstrom-Olofsson R, Nystedt A, Thomas J, Hildingsson I. Swedish caregivers' attitudes towards caesarean section on maternal request. Women Birth. 2009;22(2):57-63.
36. Koigi-Kamau R, Leting PK, Kiarie JN. Perceptions and practices of vaginal birth after Caesarean section among privately practicing obstetricians in Kenya. East African medical journal. 2005;82(12):631-6.

For peer review only - http://bmjopen.bmj.com/site/about/guidelines.xhtml

37. Kuan CI. "Suffering twice": the gender politics of cesarean sections in Taiwan. Med Anthropol Q. 2014;28(3):399-418.
38. Kucuk M. Obstetrician perceptions of the causes of high cesarean delivery rates in Turkey. International journal of gynaecology and obstetrics: the official organ of the International Federation of Gynaecology and Obstetrics. 2017;138(1):100-6.
39. Kucuk M. Defensive medicine among obstetricians and gynaecologists in Turkey. Journal of obstetrics and gynaecology : the journal of the Institute of Obstetrics and Gynaecology. 2018;38(2):200-5.
40. Kwee A, Cohlen BJ, Kanhai HH, Bruinse HW, Visser GH. Caesarean section on request: a survey in The Netherlands. European journal of obstetrics, gynecology, and reproductive biology. 2004;113(2):186-90.
41. Lundgren I, Healy P, Carroll M, Begley C, Matterne A, Gross MM, et al. Clinicians' views of factors of importance for improving the rate of VBAC (vaginal birth after caesarean section): a study from countries with low VBAC rates. BMC Pregnancy Childbirth. 2016;16(1):350.
42. Lundgren I, van Limbeek E, Vehvilainen-Julkunen K, Nilsson C. Clinicians' views of factors of importance for improving the rate of VBAC (vaginal birth after caesarean section): a qualitative study from countries with high VBAC rates. BMC Pregnancy Childbirth. 2015;15(1):196.
43. Makhija B, Verma D, Mustafa A. A root cause analysis of increasing caesarean section rates in a tertiary care private hospital in North India. International Journal of Reproduction, Contraception, Obstetrics and Gynecology. 2019;8(11):4531.
44. Munro S, Kornelsen J, Corbett K, Wilcox E, Bansback N, Janssen P. Do Women Have a Choice? Care Providers' and Decision Makers' Perspectives on Barriers to Access of Health Services for Birth after a Previous Cesarean. Birth (Berkeley, Calif). 2017;44(2):153-60.
45. Mushinski D, Zahran S, Frazier A. Physician behaviour, malpractice risk and defensive medicine: an investigation of cesarean deliveries. Health economics, policy, and law. 2021:1-19.
46. Panda S, Daly D, Begley C, Karlström A, Larsson B, Bäck L, et al. Factors influencing decision-making for caesarean section in Sweden – a qualitative study. BMC Pregnancy and Childbirth. 2018;18(1):377.
47. Peel A, Bhartia A, Spicer N, Gautham M. 'If I do 10–15 normal deliveries in a month I hardly ever sleep at home.' A qualitative study of health providers' reasons for high rates of caesarean deliveries in private sector maternity care in Delhi, India. BMC Pregnancy and Childbirth. 2018;18(1):470.
48. Perrotta C, Romero M, Sguassero Y, Straw C, Gialdini C, Righetti N, et al. Caesarean birth in public maternities in Argentina: a formative research study on the views of obstetricians, midwives and trainees. BMJ open. 2022;12(1):e053419.
49. Rudey EL, Leal MDC, Rego G. Defensive medicine and cesarean sections in Brazil. Medicine. 2021;100(1):e24176.
50. Samadi S, Gholizadeh N, Shoar N, Shoar S. Attitudes of Obstetricians toward Cesarean Delivery in Challenging Cases. J Obstet Gynaecol India. 2013;63(5):301-5.
51. Savage W, Francome C. British consultants' attitudes to caesareans. Journal of obstetrics and gynaecology : the journal of the Institute of Obstetrics and Gynaecology. 2007;27(4):354-9.
52. Wells CE. Vaginal birth after cesarean delivery: views from the private practitioner. Semin Perinatol. 2010;34(5):345-50.
53. Yang YT, Mello MM, Subramanian SV, Studdert DM. Relationship between malpractice litigation pressure and rates of cesarean section and vaginal birth after cesarean section. Med Care. 2009;47(2):234-42.
54. Yazdizadeh B, Nedjat S, Mohammad K, Rashidian A, Changizi N, Majdzadeh R. Cesarean section rate in Iran, multidimensional approaches for behavioral change of providers: a qualitative study. BMC Health Serv Res. 2011;11(1):159.

For peer review only - http://bmjopen.bmj.com/site/about/guidelines.xhtml

55. Zhao P, Cai Z, Huang A, Liu C, Li H, Yang S, et al. Why is the labor epidural rate low and cesarean delivery rate high? A survey of Chinese perinatal care providers. PloS one. 2021;16(5):e0251345.
56. Zwecker P, Azoulay L, Abenhaim HA. Effect of fear of litigation on obstetric care: a nationwide analysis on obstetric practice. American journal of perinatology. 2011;28(4):277-84.

For peer review only

For peer review only - http://bmjopen.bmj.com/site/about/guidelines.xhtml

Preferred Reporting Items for Systematic reviews and Meta-Analyses extension for Scoping Reviews (PRISMA-ScR) Checklist

SECTION	ITEM	PRISMA-ScR CHECKLIST ITEM	REPORTED ON PAGE #
**TITLE**			
Title	1	Identify the report as a scoping review.	1
**ABSTRACT**			
Structured summary	2	Provide a structured summary that includes (as applicable): background, objectives, eligibility criteria, sources of evidence, charting methods, results, and conclusions that relate to the review questions and objectives.	2
**INTRODUCTION**			
Rationale	3	Describe the rationale for the review in the context of what is already known. Explain why the review questions/objectives lend themselves to a scoping review approach.	4-5
Objectives	4	Provide an explicit statement of the questions and objectives being addressed with reference to their key elements (e.g., population or participants, concepts, and context) or other relevant key elements used to conceptualize the review questions and/or objectives.	5
**METHODS**			
Protocol and registration	5	Indicate whether a review protocol exists; state if and where it can be accessed (e.g., a Web address); and if available, provide registration information, including the registration number.	NA
Eligibility criteria	6	Specify characteristics of the sources of evidence used as eligibility criteria (e.g., years considered, language, and publication status), and provide a rationale.	5-6; Supplementary file
Information sources*	7	Describe all information sources in the search (e.g., databases with dates of coverage and contact with authors to identify additional sources), as well as the date the most recent search was executed.	5-6
Search	8	Present the full electronic search strategy for at least 1 database, including any limits used, such that it could be repeated.	Supplementary file
Selection of sources of evidence†	9	State the process for selecting sources of evidence (i.e., screening and eligibility) included in the scoping review.	5-6
Data charting process‡	10	Describe the methods of charting data from the included sources of evidence (e.g., calibrated forms or forms that have been tested by the team before their use, and whether data charting was done independently or in duplicate) and any processes for obtaining and confirming data from investigators.	6; Fig 1
Data items	11	List and define all variables for which data were sought and any assumptions and simplifications made.	Supplementary file
Critical appraisal of individual	12	If done, provide a rationale for conducting a critical appraisal of included sources of evidence; describe	NA

St. Michael's
Inspired Care. Inspiring Science.

For peer review only - http://bmjopen.bmj.com/site/about/guidelines.xhtml

SECTION	ITEM	PRISMA-ScR CHECKLIST ITEM	REPORTED ON PAGE #
sources of evidence§		the methods used and how this information was used in any data synthesis (if appropriate).	
Synthesis of results	13	Describe the methods of handling and summarizing the data that were charted.	7
**RESULTS**			
Selection of sources of evidence	14	Give numbers of sources of evidence screened, assessed for eligibility, and included in the review, with reasons for exclusions at each stage, ideally using a flow diagram.	Fig 1
Characteristics of sources of evidence	15	For each source of evidence, present characteristics for which data were charted and provide the citations.	7; Table 1
Critical appraisal within sources of evidence	16	If done, present data on critical appraisal of included sources of evidence (see item 12).	NA
Results of individual sources of evidence	17	For each included source of evidence, present the relevant data that were charted that relate to the review questions and objectives.	8-15; Table 2
Synthesis of results	18	Summarize and/or present the charting results as they relate to the review questions and objectives.	8-15
**DISCUSSION**			
Summary of evidence	19	Summarize the main results (including an overview of concepts, themes, and types of evidence available), link to the review questions and objectives, and consider the relevance to key groups.	16
Limitations	20	Discuss the limitations of the scoping review process.	19
Conclusions	21	Provide a general interpretation of the results with respect to the review questions and objectives, as well as potential implications and/or next steps.	20
**FUNDING**			
Funding	22	Describe sources of funding for the included sources of evidence, as well as sources of funding for the scoping review. Describe the role of the funders of the scoping review.	21

JBI = Joanna Briggs Institute; PRISMA-ScR = Preferred Reporting Items for Systematic reviews and Meta-Analyses extension for Scoping Reviews.

* Where *sources of evidence* (see second footnote) are compiled from, such as bibliographic databases, social media platforms, and Web sites.

† A more inclusive/heterogeneous term used to account for the different types of evidence or data sources (e.g., quantitative and/or qualitative research, expert opinion, and policy documents) that may be eligible in a scoping review as opposed to only studies. This is not to be confused with *information sources* (see first footnote).

‡ The frameworks by Arksey and O'Malley (6) and Levac and colleagues (7) and the JBI guidance (4, 5) refer to the process of data extraction in a scoping review as data charting.

§ The process of systematically examining research evidence to assess its validity, results, and relevance before using it to inform a decision. This term is used for items 12 and 19 instead of "risk of bias" (which is more applicable to systematic reviews of interventions) to include and acknowledge the various sources of evidence that may be used in a scoping review (e.g., quantitative and/or qualitative research, expert opinion, and policy document).

From: Tricco AC, Lillie E, Zarin W, O'Brien KK, Colquhoun H, Levac D, et al. PRISMA Extension for Scoping Reviews (PRISMAScR): Checklist and Explanation. Ann Intern Med. 2018;169:467–473. doi: 10.7326/M18-0850.

For peer review only - http://bmjopen.bmj.com/site/about/guidelines.xhtml
